# Fast Heck–Cassar–Sonogashira (HCS) Reactions in Green Solvents

**DOI:** 10.1021/acs.orglett.0c01269

**Published:** 2020-04-28

**Authors:** L. Ferrazzano, G. Martelli, T. Fantoni, A. Daka, D. Corbisiero, A. Viola, A. Ricci, W. Cabri, A. Tolomelli

**Affiliations:** † Department of Chemistry “G. Ciamician”, Alma Mater Studiorum—University of Bologna, Via Selmi 2, 40126 Bologna, Italy; ‡ Fresenius Kabi iPSUM Srl, I&D, Via San Leonardo 23, 45010 Villadose (RO), Italy

## Abstract

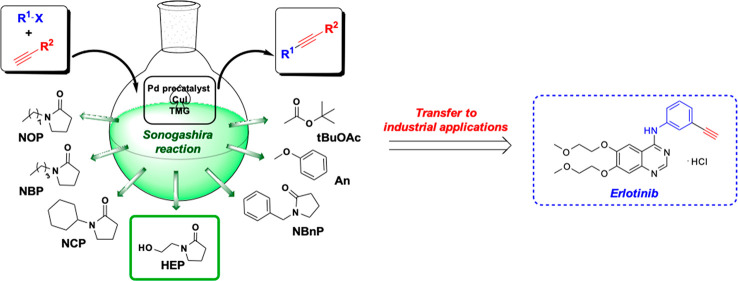

The replacement of toxic solvents with greener alternatives in Heck–Cassar–Sonogashira (HCS) cross-couplings was investigated. The fine-tuning of the HCS protocol allowed to achieve complete conversions and high speed under mild conditions. *N*-Hydroxyethylpyrrolidone (HEP) gave the best results. Moreover, the methodology was successfully applied to the synthesis of an intermediate of the anticancer drug Erlotinib, demonstrating the versatility of the new green protocol.

Palladium-catalyzed cross-coupling reactions currently represent privileged methodologies for the C–C bond formation.^1,2^ Among them, the reaction between the *sp*^2^ carbon of an aryl halide and the *sp* carbon of an alkyne allows the installation of a triple bond on the aromatic ring, opening access to subsequent transformations.

The reaction was independently reported in 1975 by Sonogashira^3^ as Pd(0)/Cu(I) catalyzed cross-coupling and by Heck^4^ and Cassar^5^ as a copper-free procedure. Since then, the Heck–Cassar–Sonogashira (HCS) reaction was successfully applied for industrial production. Several studies have investigated the influence of leaving groups, palladium ligands, cocatalyst, and bases.^6^

The greenness of industrial processes to preserve the environment and to ensure health and safety of workers has evolved from an ethic approach to an inescapable necessity.^7^ Solvents represent the main source of waste in chemical industrial processes, constituting, on average, 80–90% of the total process mass.^8^ Their selection is critical in Pd-catalyzed cross-couplings, because of the influence on the coordination sphere of the metals, the stability of the catalyst, the equilibrium, and the rate and selectivity of the reaction.^9^

In the last decades, almost 40% of the published HCS reactions were performed in *N*,*N*-dimethylformamide (DMF),^10^ which is well-known as a highly reprotoxic solvent, is classified as a substance of very high concern (SVHC), and is a potential source of *N*-dimethylnitrosamine.^11^ Other solvents also have been used, such as tetrahydrofuran (THF), dimethylsulfoxide (DMSO), 1,4-dioxane, toluene, dimethoxyethane (DME), and amines, even if not representing real greener alternatives.^9^ Alcohols and aqueous systems,^12^ ionic liquids,^13^ and bio-based solvents such as dimethylisosorbide,^14^ γ-valerolactone,^15^ and Cyrene^10^ also were investigated.

DMF has been successfully replaced in many processes by *N*-methylpyrrolidone (NMP), which displays a similar polarity profile. However, NMP has limitations, because of the potential development of toxic metabolites, such as oxidized derivatives and formaldehyde.^16^

Longer *N*-alkylpyrrolidones may offer novel opportunities, since their metabolites are less toxic than formaldehyde and related compounds typically deriving from *N*-Me oxidation in DMF and NMP. Their lower toxicity allowed their use as surfactants and their addition in cosmetic formulations.^17^

Among them, *N*-butylpyrrolidone (NBP) has been already successfully used in Heck and Suzuki cross-couplings,^18^ while less attention has been paid to pyrrolidones with longer alkyl chains (*N*-octylpyrrolidone (NOP), *N*-benzylpyrrolidone (NBnP), *N*-cyclohexylpyrrolidone (NCP)), and to *N*-hydroxyethylpyrrolidone (HEP). In addition, anisole and *tert*-butyl acetate (tBuOAc) have been included, since they are sustainable dipolar aprotic solvents.^19,20^

The target of this study is the identification of protocols for fast and efficient HCS reactions under mild conditions, using green solvents. We selected the model reaction between iodobenzene **1a** and phenylacetylene **2a**, in the presence of Pd(PPh_3_)_2_Cl_2_ and CuI at 30 °C to test the efficiency of new greener solvents, by screening several parameters (see Scheme 1 and Table 1).^21^ A high-performance liquid chromatography–ultraviolet (HPLC-UV) signal at 210 nm was used to follow the transformation of the reagents to diphenylacetylene **3a**.^22^ The reactions were stopped when no further evolution in time was observed. DMF and Cyrene experiments were performed as reference reactions and compared with literature data.^10^ Under the selected conditions, all of the solvents did not afford complete conversion (Table 1, entries 1–10). HEP gave promising results, allowing 96% conversion (Table 1, entry 4). The incomplete conversion in all the reactions reported above is mainly due to the competing side reaction of alkyne homocoupling.

**Scheme 1:**
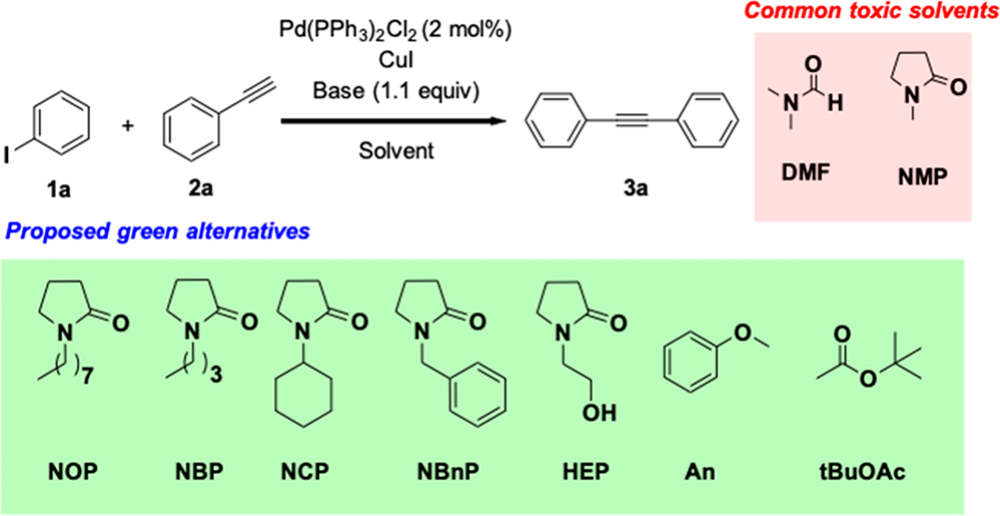
HCS Model Reaction in Green Solvents

**Table 1:** HCS Model Reaction Screening

	solvent	**2a** [equiv]	base	CuI [mol %]	time [h]	conversion [%] (yield [%])^a^
1	DMF	1.05	TEA	4	1	90
2	Cyrene	1.05	TEA	4	1	91
3	NMP	1.05	TEA	4	1	86
4	HEP	1.05	TEA	4	1	96 (90)
5	NBnP	1.05	TEA	4	1	83
6	NCP	1.05	TEA	4	1	66
7	NBP	1.05	TEA	4	1	65
8	NOP	1.05	TEA	4	1	72
9	An	1.05	TEA	4	1	86
10	*t*BuOAc	1.05	TEA	4	1	92
11	NOP	1.5	TEA	4	1	92
12	NOP	1.05	TMG	4	0.5	>99 (92)
13	NOP	1.05	TMG	1	0.5	>99 (93)
14	NBP	1.05	TMG	1	0.5	95 (90)
15	NBnP	1.05	TMG	1	0.5	>99 (90)
16	NCP	1.05	TMG	1	0.5	>99 (94)
17	HEP	1.05	TMG	1	0.5	>99 (97)^b^
18	An	1.5	TMG	1	0.5	>99 (94)
19	*t*BuOAc	1.5	TMG	1	0.5	>99 (95)
20	HEP	1.05	TEA	–	1	49
21	HEP	1.05	TMG	–	1	9

a Conversion monitored at HPLC-UV at 210 nm. The product was isolated only when conversion was >95%.

b This reaction was also performed in 10 mmol scale with similar results.

One of the worst performing solvents, NOP, was used to optimize the reaction conditions in further experiments. An excess of **2a** increased the conversion to 92% (Table 1, entry 11). Nevertheless, the strongest effect was observed when the reaction was performed by using *N*,*N*,*N*,*N*-tetramethyl guanidine (TMG) in place of the most commonly used TEA. Under these conditions, the reaction complete conversion was achieved within only 30 min, even in the presence of 1% copper co-catalyst (Table 1, entries 12 and 13). No excess of **2a** was required, since the acceleration of the HCS reaction won the competition with the homocoupling. These conditions were successfully applied to all of the other green solvents (Table 1, entries 14–19) affording **3a** in 90%–95% isolated yield. Copper-free conditions were also attempted but did not afford satisfactory results (Table 1, entries 20 and 21). HEP allowed an easy recovery of **3a** (97%), because of the complete migration of this solvent in water during the workup. This reaction was also performed on 10 mmol scale, with comparable results, in order to verify HEP recovery. Distillation of the HEP/water phase afforded the pyrrolidone in >90% yield. The E factor is comparable to the one achievable in DMF. However, HEP is a nontoxic solvent,^23^ manageable at high temperatures and easily removable by a simple workup as reported above. Furthermore, HEP can be potentially very inexpensive, being an intermediate in the green synthesis of *N*-vinylpyrrolidone from biogenic acids.^24^

The reaction was extended to substituted aryl iodides and acetylenes (see Scheme 2 and Table 2). For each couple of substrates, the mildest conditions to reach complete conversion were investigated, starting from the best conditions identified in the model reaction between **1a** and **2a**. Thus, all of the reactions were performed in HEP, using Pd(PPh_3_)_2_Cl_2_ (2 mol %) as a precatalyst, copper iodide (1 mmol %), and TMG (1.1 equiv) (see Scheme 2). The results are reported in Table 2.

**Scheme 2:**
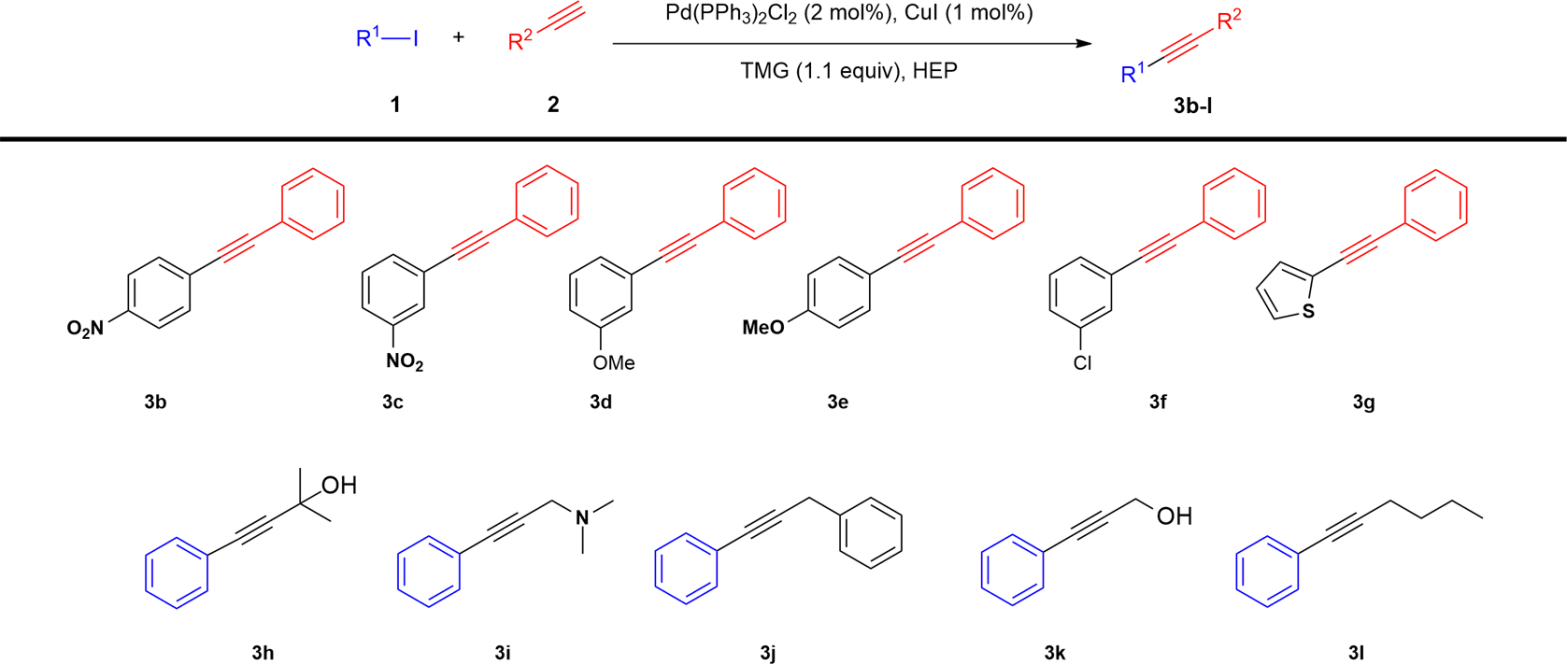
HCS Reaction on Substituted Reagents in HEP

**Table 2:** Screening of HSC Reaction Conditions with Substituted Reagents

entry	**1**	**2**	amount [equiv]	temperature, *T* [°C]	time [h]	conversion [%]^a^ (yield [%])	product
1	4-nitroiodobenzene, **1b**	phenylacetylene, **2a**	1.05	30	0.5	>99 (96)	**3b**
2	3-nitroiodobenzene, **1c**	phenylacetylene, **2a**	1.05	30	0.5	>99 (95)	**3c**
3	3-methoxyiodobenzene, **1d**	phenylacetylene, **2a**	1.05	30	0.5	>99 (98)	**3d**
4	4-methoxyiodobenzene, **1e**	phenylacetylene, **2a**	1.05	30	0.5	>99 (98)	**3e**
5	3-chloroiodobenzene, **1f**	phenylacetylene, **2a**	1.05	30	0.5	>99 (95)	**3f**
6	2-iodothiophene, **1g**	phenylacetylene, **2a**	1.05	30	0.5	>99 (98)	**3g**
7	iodobenzene, **1a**	2-methyl-3-butyn-2-ol, **2h**	1.05	30	1	>99 (94)	**3h**
8	iodobenzene, **1a**	3-dimethylamino-1-propyne, **2i**	1.5	30	1	>99 (96)	**3i**
9	iodobenzene, **1a**	3-phenyl-1-propyne, **2j**	1.5	30	0.5	>99 (98)	**3j**
10	iodobenzene, **1a**	propargyl alcohol, **2k**	1.5	50	0.5	>99 (95)	**3k**
11	iodobenzene, **1a**	1-hexyne, **2l**	1.5	50	1	>99 (95)	**3l**

a Conversion monitored at HPLC-UV at 210 nm.

The presence of electron-withdrawing and electron-donating groups and the nature of the aromatic ring of the iodide (**1b**–**1g**) did not affect reactivity, since all tested reagents displayed complete conversions to **3b**–**3g** at 30 °C in 30 min (Table 2, entries 1–6).

In contrast, the transformation of differently substituted acetylenes required to modify the reaction conditions, mainly as a consequence of a variable tendency to afford homodimerization. The cross-coupling of 2-methyl-3-butyn-2-ol **2h** with **1a** afforded complete conversion to **3h** under the standard conditions in 1 h (see Table 2, entry 7). In a similar way, 3-dimethylamino-1-propyne **2i** and 3-phenyl-1-propyne **2j** reacted with **1a** at 30 °C to give **3i** and **3j** in 1 h and 30 min, respectively (see Table 2, entries 8 and 9). In both cases, an excess of acetylene reagent (1.5 equiv) was required to reach >99% conversion.

Propargyl alcohol **2k** and 1-hexyne **2l** showed a lower reactivity and the increase of reaction temperature to 50 °C, together with an excess of reagent, was required. Under these conditions, products **3k** and **3l** were obtained in 30 min and 1 h, respectively (see Table 2, entries 10 and 11). Moving from iodides to aryl bromides, stronger reaction conditions were needed.

Using the best protocol reported in Table 1, entry 17, bromobenzene **4a** did not react (see Table 3, entry 1). Satisfactory conversion could be observed after 21 h at 60 °C with an excess of **2a** in the presence of copper (Table 3, entry 2). The copper-free protocol allowed complete conversion to be attained within 14 h (see Table 3, entry 3).

**Scheme 3:**
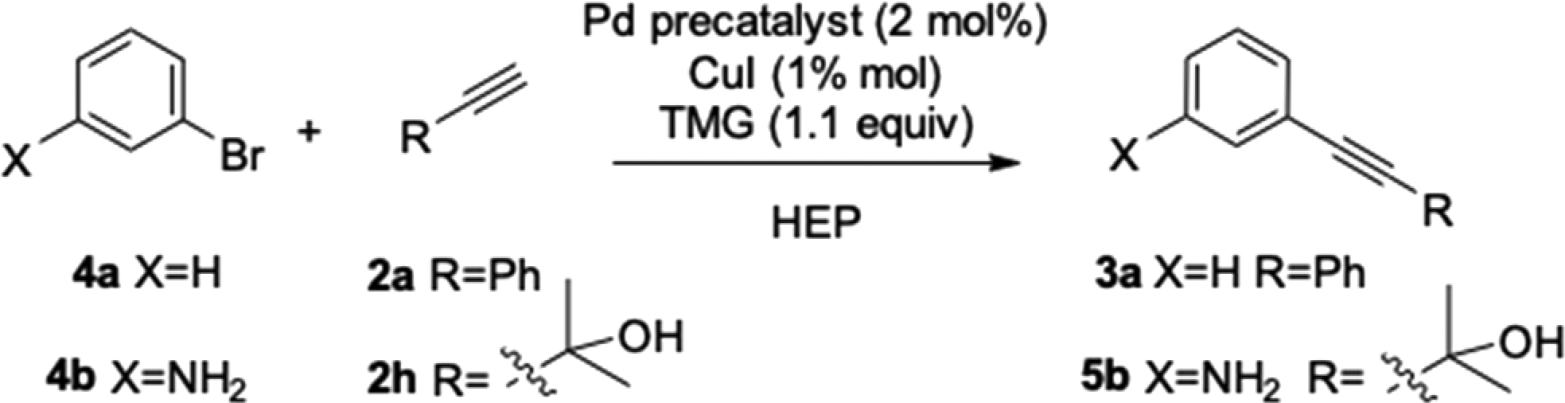
HCS Reaction on Aryl Bromides **4a** and **4b**

**Table 3:** Optimization of Reaction Conditions on Aryl Bromide Substrates

entry	aryl bromide	alkyne [equiv]	Pd precatalyst	L	CuI [mol %]	temperature, *T* [°C]	t [h]	product	conversion [%] (yield [%])^a^
1	**4a**	**2a** (1.05)	Pd(PPh_3_)_2_Cl_2_	–	1	30	21	**3a**	–
2	**4a**	**2a** (3)	Pd(PPh_3_)_2_Cl_2_	–	1	60	21	**3a**	91
3	**4a**	**2a** (3)	Pd(PPh_3_)_2_Cl_2_	–	–	60	14	**3a**	>99^b^ (93)
4	**4a**	**2a** (3)	Pd(ACN)_2_Cl_2_	Xphos	1	60	2	**3a**	>99 (95)
5	**4a**	**2a** (3)	Pd(ACN)_2_Cl_2_	Xphos	–	60	2	**3a**	>99 (95)
6	**4a**	**2a** (3)	Pd(DPPF)Cl_2_	–	1	60	7	**3a**	25
7	**4a**	**2a** (3)	Pd(DPPF)Cl_2_	–	–	60	7	**3a**	98 (95)
8	**4b**	**2h** (3)	Pd(PPh_3_)_2_Cl_2_	–	1	60	22	**5b**	50
9	**4b**	**2h** (3)	Pd(PPh_3_)_2_Cl_2_	–	–	60	22	**5b**	95 (80)^c^
10	**4b**	**2h** (3)	Pd(ACN)_2_Cl_2_	Xphos	1	80	22	**5b**	17
11	**4b**	**2h** (3)	Pd(ACN)_2_Cl_2_	Xphos	–	60	14	**5b**	>99 (85)^c^
12	**4b**	**2h** (3)	Pd(DPPF)Cl_2_	–	1	80	22	**5b**	86
13	**4b**	**2h** (3)	Pd(DPPF)Cl_2_	–	–	60	3	**5b**	>99 (86)^c^

a Conversion monitored at HPLC-UV at 210 nm. The product was isolated only when conversion was >95%.

b Conversion was 94% after 7 h.

c Yield was calculated after telescoping transformation to **6b**.

To increase the reaction speed, the inexpensive Pd(PPh_3_)_2_Cl_2_ had to be replaced by Pd(ACN)_2_Cl_2_/Xphos or Pd(DPPF)Cl_2_.

Since its first use in HCS reactions in 2003 by Gelman and Buchwald,^25^ Pd catalyst containing Xphos ligand has been reported to give extraordinary results in several applications. Complete conversion of **4a** into **3a** was obtained within 2 h with Pd(ACN)_2_Cl_2_/Xphos, with or without copper (Table 3, entries 4 and 5). The use of Pd(DPPF)Cl_2_^26^ did not produce comparable results, since 98% conversion was observed in the Heck-Cassar copper-free reaction only after 7 h (Table 3, entry 7), while the presence of the copper co-catalyst completely inhibited the reaction (Table 3, entry 6).^25^ In order to have a further demonstration of the general applicability of our procedure, we selected an industrially relevant process requiring a Sonogashira reaction step (Scheme 3).

As an example, the synthesis of an intermediate of the pharmacologically active molecule Erlotinib resulted in being suitable for our scope.

Erlotinib hydrochloride is an oral antitumor drug^27^ that acts by reversibly and selectively inhibiting epidermal growth factor receptor (EGFR) type 1 tyrosine kinase activity in many types of human cancers affecting lung, pancreas, ovary, kidney, stomach, liver, and breast tissue.

The industrial process for its production (Scheme 4),^28^ requires a Sonogashira reaction to convert 3-bromoaniline **4b** to 3-ethynylaniline **6b**. Thus, the reaction between **4b** and 2-methyl-3-butyn-2-ol **2h** in HEP was studied. As reported in Table 3, the Pd(ACN)_2_Cl_2_/Xphos catalytic system allowed to achieve complete conversion to the intermediate **5b** only after 14 h without CuI (Table 3, entry 11). The comparison of entries 5 and 11 in Table 3 shows a decreased efficiency of the Pd catalyst in the presence of the aniline fragment.

**Scheme 4:**
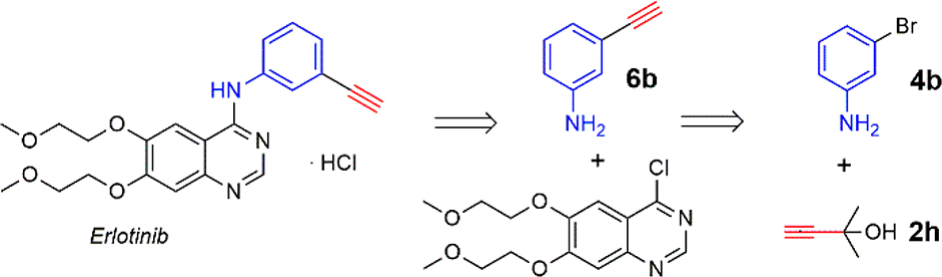
Retrosynthetic Approach to the Synthesis of Erlotinib

The best catalytic system for the reaction of **4b** resulted in being Pd(DPPF)Cl_2_ under copper-free HC conditions, which allowed complete conversion to be attained within 3 h (Table 3, entry 13). As already reported by Buckwald at high temperature, the copper co-catalyst favors the aryl alkyne oligomerization.^25^

Intermediate **5b** was not isolated and directly transformed under telescoping conditions with toluene/NaOH into **6b**.^29^

In summary, several green solvents have been tested to replace toxic DMF and *N*-methylpyrrolidone (NMP) in the HCS cross-coupling between aryl halides and substituted acetylenes.

*N*-hydroxyethyl pyrrolidone (HEP) has been shown to be the most suitable candidate, allowing one to find mild conditions for poorly reactive alkynes and aryl bromides. The versatility of the solvent is particularly important when complex molecules are synthesized via multistep procedures. The excellent results obtained in the synthesis of an intermediate of the drug Erlotinib encourage in the application of HEP on a large scale.

## Supporting Information Available

The Supporting Information is available free of charge on the ACS Publications Web site. (file pdf) The Supporting Information is available free of charge at https://pubs.acs.org/doi/10.1021/acs.orglett.0c01269.

General procedures; HPLC-UV chromatograms; characterization of known compounds; relative response factor calculation; the complete screening of conditions (Table 1S) (PDF)
