# Supplementary material for: Fast Heck–Cassar–Sonogashira (HCS) Reactions in Green Solvents
Source: Org Lett. 2020 Apr 28;22(10):3969–73. doi: 10.1021/acs.orglett.0c01269 (PMC8007125; doi:10.1021/acs.orglett.0c01269)
Supplement: Supplementary file 1 — ol0c01269_si_001.pdf [file ol0c01269_si_001.pdf]

SUPPORTING INFORMATION

Fast Heck–Cassar–Sonogashira (HCS) Reactions in Green Solvents

L. Ferrazzano,[†] G. Martelli,[*†] T. Fantoni,[†] A. Daka,[†] D. Corbisiero,[†] A. Viola,[‡] A. Ricci,[‡] W. Cabri,[†‡*] A. Tolomelli,[†]

† Department of Chemistry "G. Ciamician", Alma Mater Studiorum - University of Bologna, Via Selmi 2, 40126 Bologna, Italy.

‡ Fresenius Kabi iPSUM Srl, I&D, Via San Leonardo 23, 45010 Villadose (RO), Italy.

Table of contents:

1. **Introductory Section**

    1.1.General methods
    1.2 General procedures and yields:
    - *General procedure for the synthesis of **3a***
    - *General procedure for the synthesis of **3b-l***
    - *General procedure for the synthesis of **3a** and **5b** from **4a-b***
    - *Table of Isolated yields*
    - *Table with all the experiments for method's fine tuning*

2. **Experimental detail section**

    2.1 HPLC-UV chromatograms of Sonogashira reactions
    2.2 Characterization data for products **3a-l**
    2.3 HPLC-UV chromatograms of commercial reagents
    2.4 Relative response factor calculation
    - *RRF between iodobenzene and diphenylacetylene*
    - *RRF between bromobenzene and diphenylacetylene*

3. **References**

1. **Introductory Section**

1.1 General methods:

Commercial reagents (reagent grade, >99%) were used as received without additional purification. Solvents (*N,N*-dimethylformamide (DMF), Cyrene, *N*-Butylpyrrolidone (NBP), *N*-Benzylpyrrolidone (NBnP), *N*-Hydroxyethylpyrrolidone (HEP), *N*-Octylpyrrolidone (NOP), *N*-Cyclohexylpyrrolidone (NCP), anisole (An), *tert*-butyl acetate (tBuOAc) are commercially available and were used after degasification.
^{1}H NMR spectra were recorded with an Agilent-Technologies-Varian INOVA 400 MHz instrument 1H/19F/X 5 mm PFG ATB Broadband Probe, VT, single, double and triple resonance, z-axis pulsed-field gradients, serves broadband probe and customized variable temperature – 5mm Broadband probe.
HPLC-UV analysis were recorded with an Agilent 1260 InfinityLab instrument. Column: Zorbax® SB-C18; particle Size 5 µm; pore size 100 Å; length 250 mm, internal diameter: 4.6 mm. Mobile phase: H_2O/CH_3CN, 0.5 mL min^{-1}, gradient from 30 to 80% of CH_3CN in 8 min, 80% of CH_3CN from 8 to 22 min, from 80 to 10% from 22 to 24 min and 10% from 24 to 30 min; 30°C; injection volume: 20 µL.
GC-MS analysis were recorded with a Hewlett-Packard 5971 spectrometer with GC injection and EI ionization at 70 eV coupled with an Agilent Technologies MSD1100 single-quadrupole mass spectrometer, reported as: m/z (rel. intensity).

1.2 General procedures:

Starting aryl halides and acetylenes are commercially available; synthetized compounds **3a-n** and **5b** are known. Structures of all the obtained known compounds were assessed by ^{1}H NMR and HPLC-UV or GC-MS analysis resulting fully consistent with data reported in databases or literature. [1-11]

- *General procedure for the synthesis of* ***3a***

To an oven-dried 10 mL schlenk purged under N_2 atmosphere, $Pd(PPh_3)_2Cl_2$ (7.0 mg, 0.01 mmol, 2%) and CuI (from 1% to 4%, see entries in Table 1), were dissolved in 1 mL of the desired degassed solvent (*N,N*-dimethylformamide (DMF), Cyrene, *N*-Butylpyrrolidone (NBP), *N*-Benzylpyrrolidone (NBnP), *N*-Hydroxyethylpyrrolidone (HEP), *N*-Octylpyrrolidone (NOP), *N*-Cyclohexylpyrrolidone (NCP), anisole (An), *tert*-butyl acetate (tBuOAc), see Table 1). The other reagents were then added in the following order: base (triethylamine (TEA), *N,N,N,N*-tetramethyl guanidine (TMG), 0.55 mmol, 1.1 eq, see Table 1), iodobenzene **1a** (56 µL, 0.5 mmol, 1.0 eq) and phenylacetylene 2**a** (from 1.05 to 1.5 eq, see Table 1). The reaction mixture was heated to 30°C with an oil bath and maintained at this temperature under stirring. Samples for HPLC monitoring were taken at set time intervals (30 min, 60 min), after a mini quenching with H_2O and dilution with CH_3CN.
Conversions of limiting iodobenzene **1a** into diphenylacetylene **3a** were monitored at set time intervals (30 min, 60 min) through HPLC-UV analysis at 210 nm, after correction with the appropriate Relative Response Factor (see following section on Relative Response Factor calculation).
In reactions with conversions >99%, after 1 hour the mixture was quenched with H_2O (3 mL) and extracted with cyclohexane (3 x 5 mL). The collected organic phases were washed with brine, dried over anhydrous Na_2SO_4 and concentrated under reduced pressure to give a yellow oil, which was purified by flash chromatography (silica gel, 100% cyclohexane) to afford compound **3a** as a white solid with quantitative yields if the reactions were performed in HEP or with 90-96% isolated yields with other *N*-alkylpyrrolidones (see Table 1).

The synthesis of **3a** was optimized using the following conditions: $Pd(PPh_3)_2Cl_2$ (7.0 mg, 0.5 mmol, 2%), CuI (1.0 mg, 0.005 mmol, 1%), TMG (69 µL, 0.55 mmol, 1.1 eq), iodobenzene (56 µL, 0.5

mmol, 1.0 eq), phenylacetylene (58 µL, 0.525 mmol, 1.05 eq), HEP (1 mL), 30°C, 30 min (see Entry 17 Table 1). The same conditions were applied to a 10 mmol-scale reaction to verify the recovery of HEP. To this purpose the water/HEP phase was distilled under vacuum to afford 18.3 ml of HEP (91.5% recovery). The recovery process wasn't further optimized.

- *General procedure for the synthesis of **3b-l***

To an oven-dried 10 mL schlenk purged under N_2 atmosphere, $Pd(PPh_3)_2Cl_2$ (7.0 mg, 0.01 mmol, 2%) and CuI (1.0 mg, 0.005 mmol, 1%), were dissolved in 1 mL of degassed HEP. The other reagents were then added in the following order: TMG (69 µL, 0.55 mmol, 1.1 eq), aryl iodide (0.5 mmol, 1.0 eq, see entries in Table 2) and acetylene (from 1.05 to 1.5 eq, see entries in Table 2). The reaction mixture was heated to 30°C or 50°C (according to what reported in Table 2) with an oil bath and maintained at this temperature under stirring. Samples for HPLC monitoring were taken at set time intervals (30 min, 60 min), after a mini quenching with H_2O and dilution with CH_3CN.
After 1 hour, the mixture was quenched with H_2O (3 mL) and extracted with cyclohexane (3 x 5 mL). The collected organic phases were washed with brine, dried over anhydrous Na_2SO_4 and concentrated under reduced pressure to give a yellow oil, which was purified by flash chromatography (silica gel, 100% cyclohexane) to afford compounds **3b-l** (see Table 2).

- *General procedure for the synthesis of **3a** and **5b** from **4a-b***

To an oven-dried 10 mL schlenk purged under N_2 atmosphere, Pd catalyst (0.01 mmol, 2%, see entries in Table 3), CuI (1.0 mg, 0.005 mmol, 1%, if specified in Table 3) and the desired ligand (0.02 mmol, 4%, if specified in Table 3) were dissolved in 1 mL of degassed HEP. The other reagents were then added in the following order: TMG (69 µL, 0.55 mmol, 1.1 eq), aryl bromide **4a** or **4b** (0.5 mmol, 1.0 eq, see entries in Table 3) and acetylene **2a** or **2h** (1.5 mmol, 3.0 eq, see entries in Table 3). The reaction mixture was heated (see temperatures in Table 3) with an oil bath and maintained at this temperature under stirring. Samples for HPLC monitoring were taken at set time intervals (see entries in Table 3), after a mini quenching with H_2O and dilution with CH_3CN.
Conversions of limiting bromobenzene **4a** into diphenylacetylene **3a** were monitored at set time intervals through HPLC-UV analysis at 210 nm, after correction with the appropriate Relative Response Factor (see following section on Relative Response Factor calculation).
In reactions with conversions >99% (see times in Table 3), the mixture was quenched with H_2O (3 mL) and extracted with cyclohexane (3 x 5 mL). The collected organic phases were washed with brine, dried over anhydrous Na_2SO_4 and concentrated under reduced pressure to give a yellow oil, which was purified by flash chromatography (silica gel, 100% cyclohexane) to afford compound **3a**.

Conversions of limiting 3-bromoaniline **4b** into 1-amino-3-ethynyl-benzene **5b** were monitored at set time intervals through HPLC-UV analysis at 210 nm. For reactions with conversions >99% (see Table 3), the work-up for compound **5b** was performed in toluene. The collected organic toluene layers were concentrated under reduced pressure to the final volume of 5 mL and NaOH (0.5 mmol, 20 mg) was added and the mixture was refluxed for 3h with an oil bath. The reaction was cooled to rt, and washed with water. The organic phase was dried over anhydrous Na_2SO_4 in the presence of montmorrilonite. After filtration, the solution was concentrated under reduced pressure to give 1-amino-3-ethynyl-benzene **6b** as a yellowish oil. Full characterization of the final product is reported below.
The synthesis of **3a** was optimized using the following conditions: $Pd(ACN)_2Cl_2$ (2.6 mg, 0.01 mmol, 2%), Xphos (10 mg, 0.02 mmol, 4%), TMG (69 µL, 0.55 mmol, 1.1 eq), bromobenzene **4a** (52 µL, 0.5 mmol, 1.0 eq), phenylacetylene **2a** (165 µL, 1.5 mmol, 3.0 eq), HEP (1 mL), 60°C, 2 hours (see Entry 4 Table 3).
The synthesis of **5b** was optimized using the following conditions: $Pd(DPPF)Cl_2{\cdot}CH_2Cl_2$ (8.1 mg, 0.01 mmol, 2%), TMG (69 µL, 0.55 mmol, 1.1 eq), 3-bromoaniline **4b** (54 µL, 0.5 mmol, 1.0 eq), 2-

methyl-3-butyn-2-ol **2h** (145 µL, 1.5 mmol, 3.0 eq), HEP (1 mL), 60°C, 3 hours (see Entry 10 Table 3). Compound **5b** was not isolated and directly converted into **6b**.

Table S1. Yields of isolated products

Entry in main text	Aryl halogenide	Alkyne	Solvent	Product	Conv %[a]	Yield (%)	Yield (mg)
**From Table 1**							
4	**1a**	**2a**	HEP	**3a**	96	90	80
12	**1a**	**2a**	NOP	**3a**	>99	92	82
13	**1a**	**2a**	NOP	**3a**	>99	93	83
14	**1a**	**2a**	NBP	**3a**	95	90	80
15	**1a**	**2a**	NBnP	**3a**	>99	90	80
16	**1a**	**2a**	NCP	**3a**	>99	94	84
17	**1a**	**2a**	HEP	**3a**	>99	97	86
18	**1a**	**2a**	An	**3a**	>99	94	84
19	**1a**	**2a**	tBuOAc	**3a**	>99	95	85
**From Table 2**							
1	**1b**	**2a**	HEP	**3b**	>99	96	107
2	**1c**	**2a**	HEP	**3c**	>99	95	106
3	**1d**	**2a**	HEP	**3d**	>99	98	102
4	**1e**	**2a**	HEP	**3e**	>99	98	101
5	**1f**	**2a**	HEP	**3f**	>99	95	101
6	**1g**	**2a**	HEP	**3g**	>99	98	90
7	**1a**	**2h**	HEP	**3h**	>99	94	75
8	**1a**	**2i**	HEP	**3i**	>99	96	76
9	**1a**	**2j**	HEP	**3j**	>99	98	94
10	**1a**	**2k**	HEP	**3k**	>99	95	63
11	**1a**	**2l**	HEP	**3l**	>99	95	75
**From Table 3**							
3	**4a**	**2a**	HEP	**3a**	>99	93	83
4	**4a**	**2a**	HEP	**3a**	>99	95	85
5	**4a**	**2a**	HEP	**3a**	>99	95	85
7	**4a**	**2a**	HEP	**3a**	98	95	85
9	**4b**	**2h**	HEP	**5b**	95[b]	80[c]	47[c]
11	**4b**	**2h**	HEP	**5b**	>99[b]	85[c]	49[c]
13	**4b**	**2h**	HEP	**5b**	>99[b]	86[c]	50[c]

[a]Conversion monitored at HPLC-UV at 210 nm.
[b]Conversion into **5b** that was not isolated and directly converted into **6b**.
[c]The yields refer to isolated amounts of **6b**.

Complete screening of conditions for the reaction of 1a with 2a

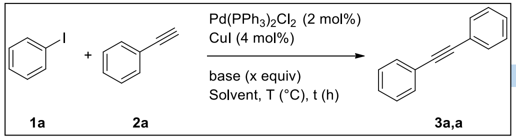

RRF = 3.05

Entries underlined in yellow are reported in table 1

	solvent (x M)	2a (equiv)	base (equiv)	Pd(PPh3)2Cl2 (%mol)	Co-catalyst (mol%)	T (°C)	Conversion (%)_30min	Conversion (%)_1h	Conversion (%)_2h
1	DMF (0.5 M)	1.05	Et$_3$N (1.1 eq)	2 mol	4	20	n.d.	n.d.	85
2	Cyrene (0.5 M)	1.05	Et$_3$N (1.1 eq)	2 mol	4	20	n.d.	n.d.	80
3	Cyrene (0.5 M)	1.05	Et$_3$N (3 eq)	2 mol	4	20	n.d.	n.d.	97
4	1-(2-Hydroxyethyl)-2-pyrrolidone (0.5 M)	1.05	Et$_3$N (1.1 eq)	2 mol	4	20	n.d.	n.d.	95
5	DMF (0.5 M)	1.05	Et$_3$N (1.1 eq)	2 mol	4	30	84	90	90
6	Cyrene (0.5 M)	1.05	Et$_3$N (1.1 eq)	2 mol	4	30	84	91	91
7	1-(2-Hydroxyethyl)-2-pyrrolidone (0.5 M)	3	Et$_3$N (1.1 eq)	2 mol	4	30	n.d.	100	
8	1-Benzyl-2-pyrrolidinone (0.5 M)	3	Et$_3$N (1.1 eq)	2 mol	4	30	n.d.	100	
9	1-(2-Hydroxyethyl)-2-pyrrolidone (0.5 M)	1.05	Et$_3$N (1.1 eq)	2 mol	4	30	86	96	
10	1-(2-Hydroxyethyl)-2-pyrrolidone (0.5 M)	1.05	TMG (1.1 eq)	2 mol	4	30	96	100	
11	1-(2-Hydroxyethyl)-2-pyrrolidone (0.5 M)	1.05	Et$_3$N (1.1 eq)	2 mol	-	30	n.d.	49	
12	1-(2-Hydroxyethyl)-2-pyrrolidone (0.5 M)	1.05	TMG (1.1 eq)	2 mol	-	30		9	
13	1-(2-Hydroxyethyl)-2-pyrrolidone (0.5 M)	1.05	Et$_3$N (1.1 eq)	2 mol	1	30	n.d.	88	
14	1-(2-Hydroxyethyl)-2-pyrrolidone (0.5 M)	1.05	TMG (1.1 eq)	2 mol	1	30	100		
15	1-(2-Hydroxyethyl)-2-pyrrolidone (0.5 M)	1.05	TMG (1.1 eq)	2 mol	1	30	100	(10 mmol scale)	
16	1-(2-Hydroxyethyl)-2-pyrrolidone (0.5 M)	1.05	TMG (1.1 eq)	1 mol	0.5	30	97	100	
17	1-(2-Hydroxyethyl)-2-pyrrolidone (0.5 M)	1.05	TMG (1.1 eq)	0.3 mol	0.15	30	82	88	93
18	1-(2-Hydroxyethyl)-2-pyrrolidone (0.5 M)	1.5	Et$_3$N (1.1 eq)	2 mol	4	30	100		
19	1-(2-Hydroxyethyl)-2-pyrrolidone (0.5 M)	2	Et$_3$N (1.1 eq)	2 mol	4	30	100		
20	1-Benzyl-2-pyrrolidinone (0.5 M)	1.05	Et$_3$N (1.1 eq)	2 mol	4	30	78	83	
21	1-Benzyl-2-pyrrolidinone (0.5 M)	1.5	Et$_3$N (1.1 eq)	2 mol	4	30	82	87	
22	1-Benzyl-2-pyrrolidinone (0.5 M)	1.5	TMG (1.1 eq)	2 mol	4	30	100		
23	1-Benzyl-2-pyrrolidinone (0.5 M)	1.05	TMG (1.1 eq)	2 mol	1	30	100		
24	1-Cyclohexyl-2-pyrrolidone (0.5 M)	1.05	Et$_3$N (1.1 eq)	2 mol	4	30	59	66	
25	1-Cyclohexyl-2-pyrrolidone (0.5 M)	1.5	Et$_3$N (1.1 eq)	2 mol	4	30	77	86	
26	1-Cyclohexyl-2-pyrrolidone (0.5 M)	1.5	TMG (1.1 eq)	2 mol	4	30	95	95	
27	1-Cyclohexyl-2-pyrrolidone (0.5 M)	1.05	TMG (1.1 eq)	2 mol	1	30	100		
28	1-butyl-2-pyrrolidone (0.5 M)	1.05	Et$_3$N (1.1 eq)	2 mol	4	30	55	65	
29	1-butyl-2-pyrrolidone (0.5 M)	1.05	TMG (1.1 eq)	2 mol	4	30	100		
30	1-butyl-2-pyrrolidone (0.5 M)	1.05	TMG (1.1 eq)	2 mol	1	30	92	94	
31	1-methyl-2-pyrrolidone (0.5 M)	1.05	Et$_3$N (1.1 eq)	2 mol	4	30	79	86	86
32	1-methyl-2-pyrrolidone (0.5 M)	1.05	TMG (1.1 eq)	2 mol	1	30	93	95	
33	1-methyl-2-pyrrolidone (0.5 M)	1.05	TMG (1.1 eq)	2 mol	4	30	98	98	
34	Anisole (0.5 M)	1.05	Et$_3$N (1.1 eq)	2 mol	4	30	81	86	
35	Anisole (0.5 M)	1.5	Et$_3$N (1.1 eq)	2 mol	4	30	88	92	
36	Anisole (0.5 M)	1.5	TMG (1.1 eq)	2 mol	4	30	100		
37	Anisole (0.5 M)	1.05	TMG (1.1 eq)	2 mol	1	30	90	90	
38	Anisole (0.5 M)	1.05	TMG (1.1 eq)	2 mol	4	30	97	97	
39	tBuOAc (0.5 M)	1.05	Et$_3$N (1.1 eq)	2 mol	4	30	92	92	
40	tBuOAc (0.5 M)	1.5	Et$_3$N (1.1 eq)	2 mol	4	30	90	100	
41	tBuOAc (0.5 M)	1.5	TMG (1.1 eq)	2 mol	4	30	100		
42	tBuOAc (0.5 M)	1.05	TMG (1.1 eq)	2 mol	1	30	65	91	
43	tBuOAc (0.5 M)	1.05	TMG (1.1 eq)	2 mol	4	30	97	97	
44	tBuOAc (0.5 M)	1.5	TMG (1.1 eq)	2 mol	1	30	100		
45	1-Octyl-2-pyrrolidone (0.5 M)	1.05	Et$_3$N (1.1 eq)	2 mol	4	30	56	72	
46	1-Octyl-2-pyrrolidone (0.5 M)	1.05	TMG (1.1 eq)	2 mol	4	30	100		
47	1-Octyl-2-pyrrolidone (0.5 M)	1.5	Et$_3$N (1.1 eq)	2 mol	4	30	84	92	
48	1-Octyl-2-pyrrolidone (0.5 M)	1.05	TMG (1.1 eq)	2 mol	4	30	100		
49	1-Octyl-2-pyrrolidone (0.5 M) + 5% H2O	1.05	Et$_3$N (1.1 eq)	2 mol	4	30	83	85	
50	1-Octyl-2-pyrrolidone (0.5 M)	1.05	Et$_3$N (3 eq)	2 mol	4	30	86	89	
51	1-Octyl-2-pyrrolidone (0.5 M)	1.05	TMG (1.1 eq)	2 mol	1	30	100		

2. Experimental detail section

2.1 HPLC-UV chromatograms of Sonogashira crude reactions:

- *Table 1 (reactions between phenylacetylene and iodobenzene, <u>relative percentages are reported in table 1</u>)*

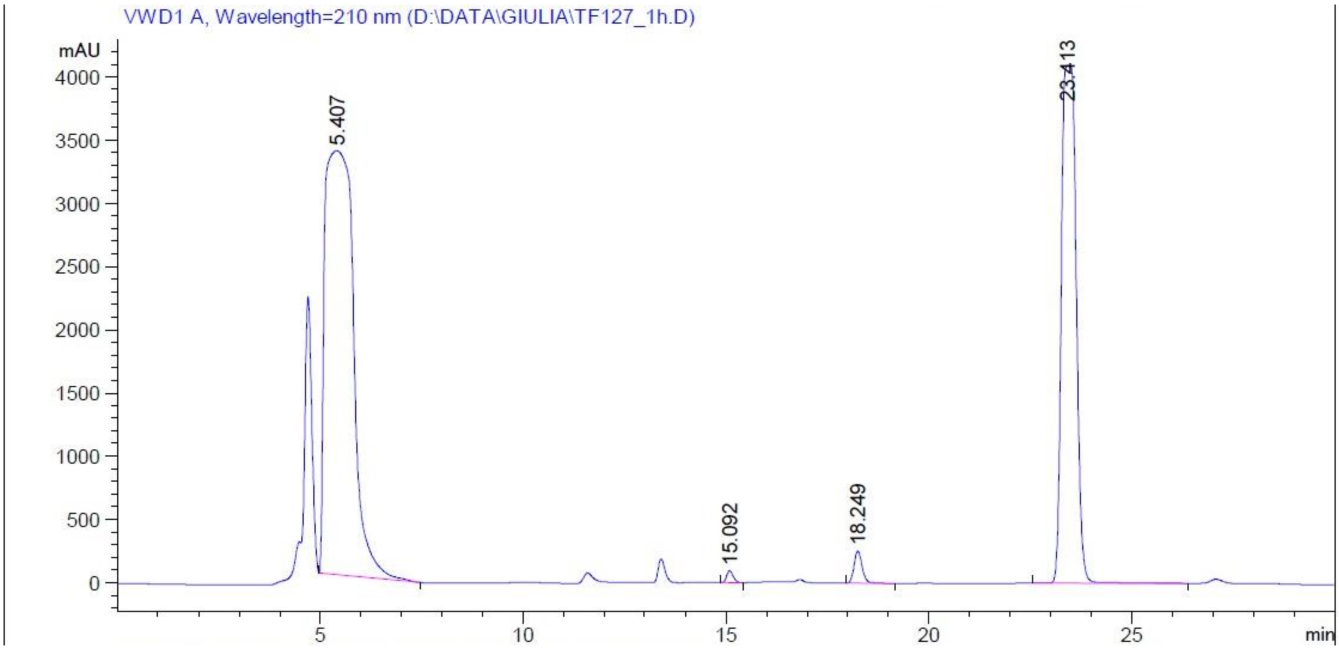

Figure S1: HPLC chromatogram of Sonogashira reaction in DMF (**entry 1 Table 1**) after 1 hour at 210 nm; peak at 5.407 min = DMF; peak at 15.092 min = residual phenylacetylene; peak at 18.249 min = residual iodobenzene; peak at 23.413 min = diphenylacetylene

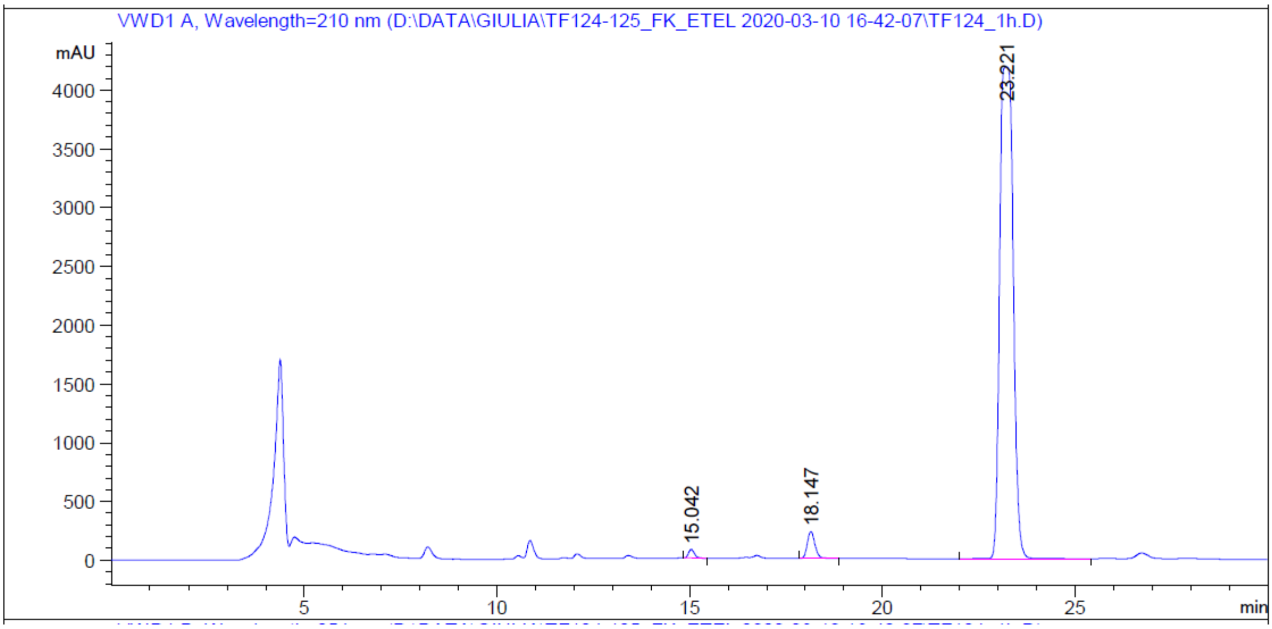

Figure S2: HPLC chromatogram of Sonogashira reaction in Cyrene (**entry 2 Table 1**) after 1 hour at 210 nm; peak at 15.042 min = residual phenylacetylene; peak at 18.147 min = residual iodobenzene, peak at 23.221 min = diphenylacetylene

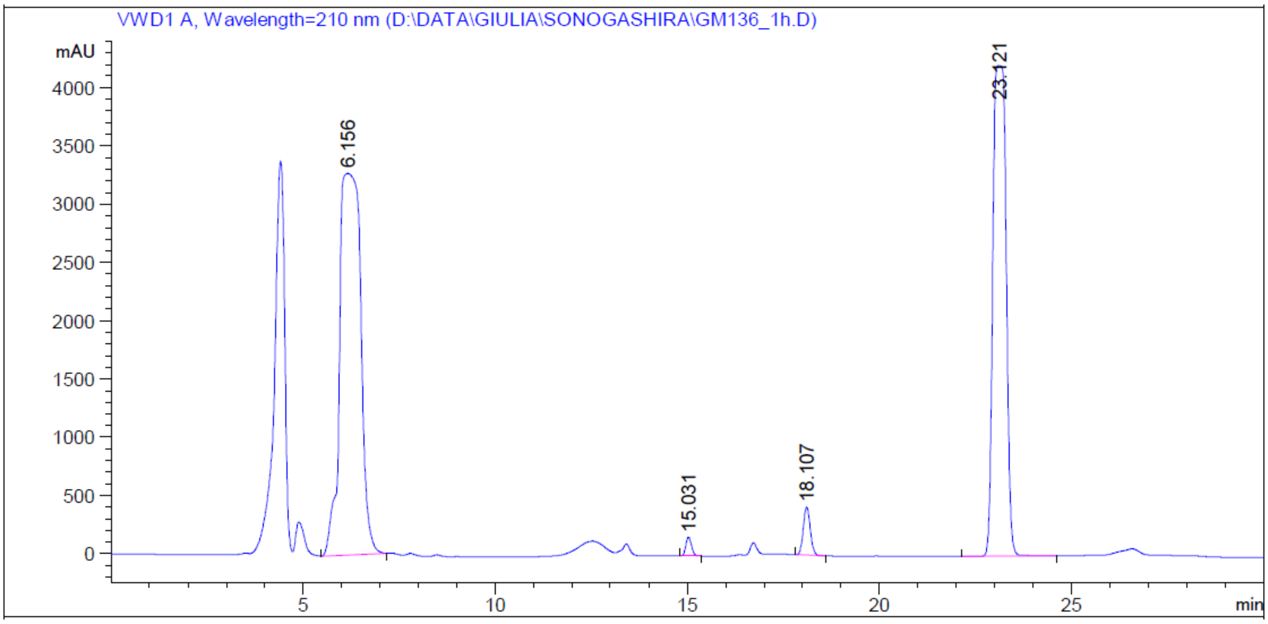

Figure S3: HPLC chromatogram of Sonogashira reaction in NMP (**entry 3 Table 1**) after 1 hour at 210 nm; peak at 6.156 min = NMP; peak at 15.031 min = residual phenylacetylene; peak at 18.107 min = residual iodobenzene; peak at 23.121 min = diphenylacetylene

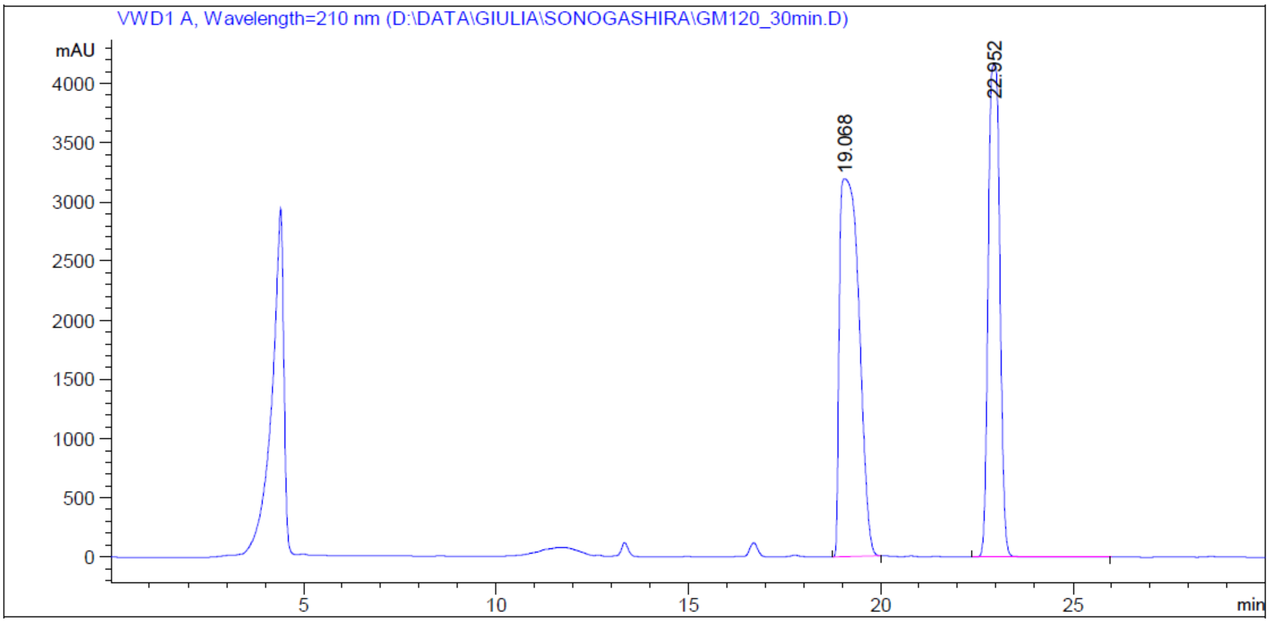

Figure S4: HPLC chromatogram of Sonogashira reaction in NOP with best conditions (**entry 13 Table 1**) after 30 minutes at 210 nm; peak at 19.068 min = NOP; peak at 22.952 min = diphenylacetylene

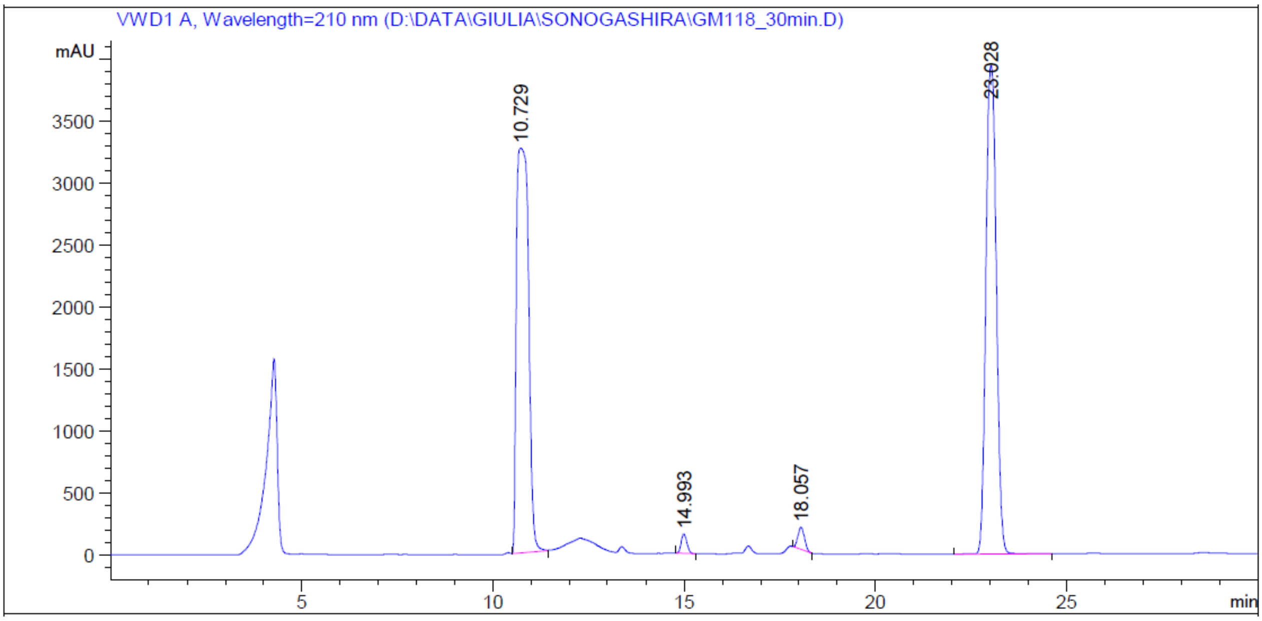

Figure S5: HPLC chromatogram of Sonogashira reaction in NBP with best conditions (**entry 14 Table 1**) after 30 minutes at 210 nm; peak at 10.729 min = NBP; peak at 14.993 min = residual phenylacetylene; peak at 18.057 min = residual iodobenzene, peak at 23.028 min = diphenylacetylene

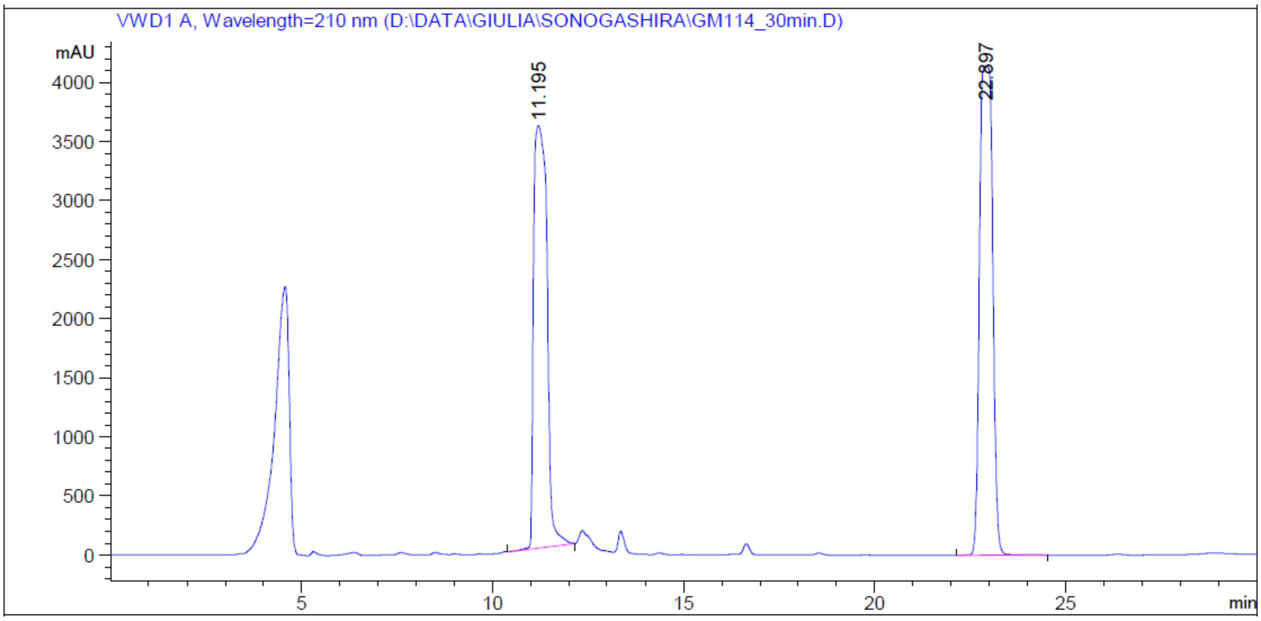

Figure S6: HPLC chromatogram of Sonogashira reaction in NBnP with best conditions (**entry 15 Table 1**) after 30 minutes at 210 nm; peak at 11.195 min = nBnP, peak at 22.897 min = diphenylacetylene

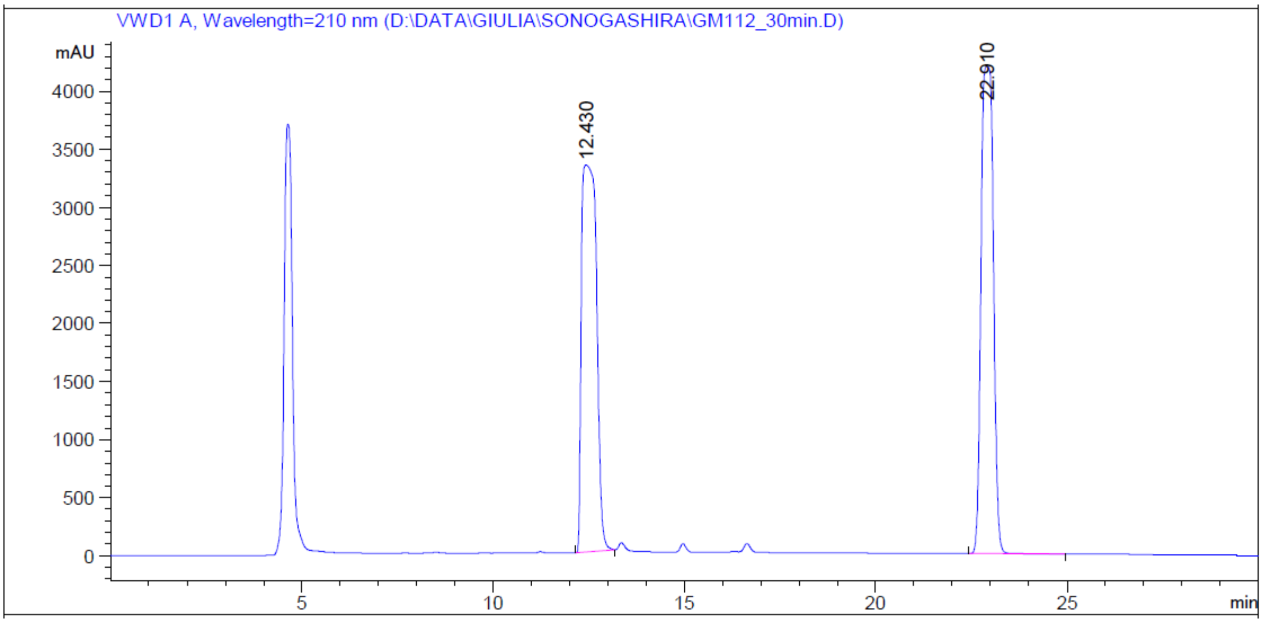

Figure S7: HPLC chromatogram of Sonogashira reaction in NCP with best conditions (**entry 16 Table 1**) after 30 minutes at 210 nm; peak at 12.430 min = NCP, peak at 22.910 min = diphenylacetylene

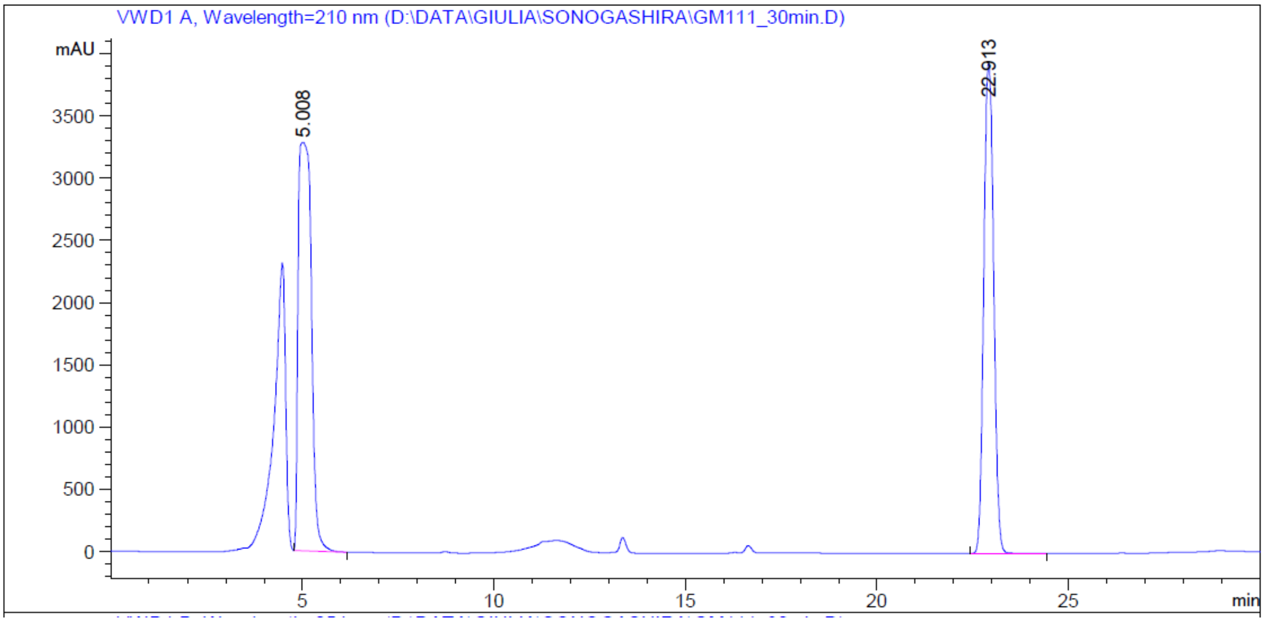

Figure S8: HPLC chromatogram of Sonogashira reaction in HEP with best conditions (**entry 17 Table 1**) after 1 hour at 210 nm; peak at 5.008 min = HEP; peak at 22.913 min = diphenylacetylene

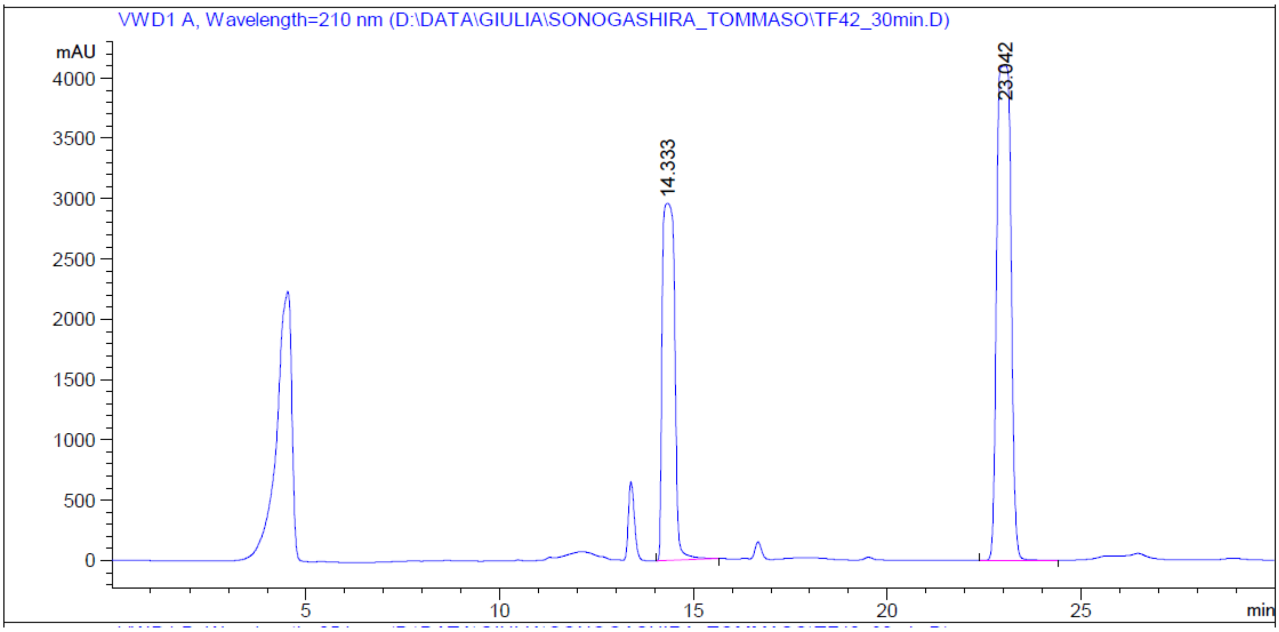

Figure S9: HPLC chromatogram of Sonogashira reaction in An with best conditions (**entry 18 Table 1**) after 30 minutes at 210 nm; peak at 14.333 min = An; peak at 23.042 min = diphenylacetylene

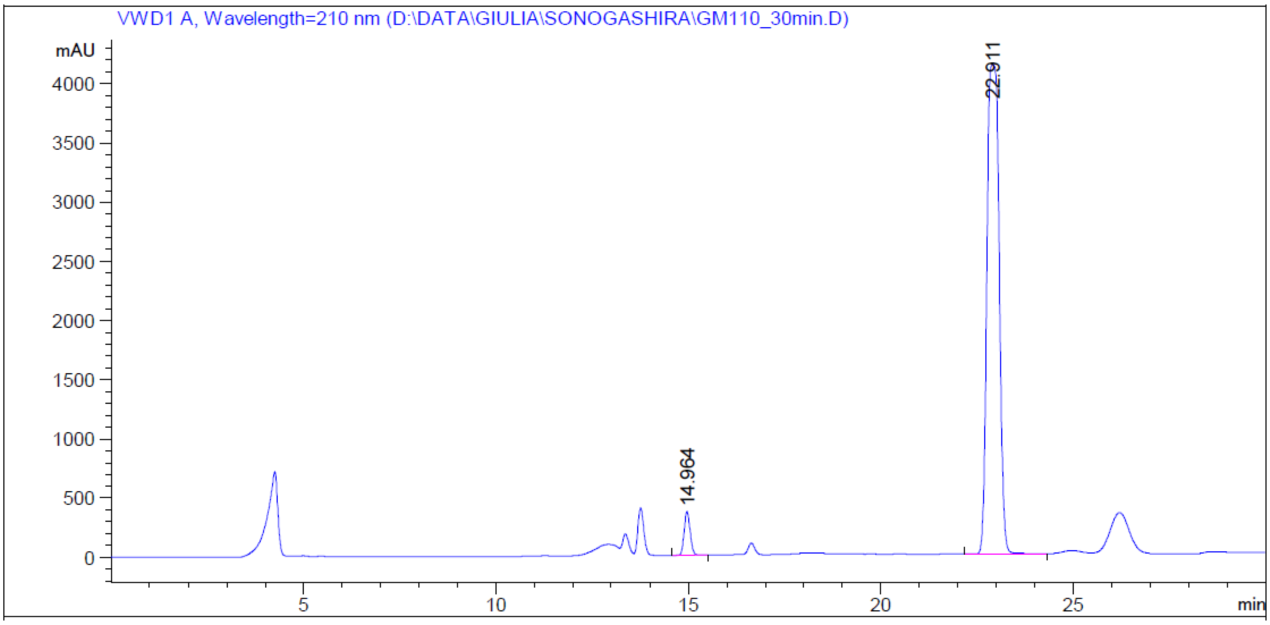

Figure S10: HPLC chromatogram of Sonogashira reaction in tBuOAc with best conditions (**entry 19 Table 1**) after 30 minutes at 210 nm; peak at 14.964 min = residual phenylacetylene; peak at 22.911 min = diphenylacetylene

- *Table 2 (screening of aryl iodides and acetylenes)*

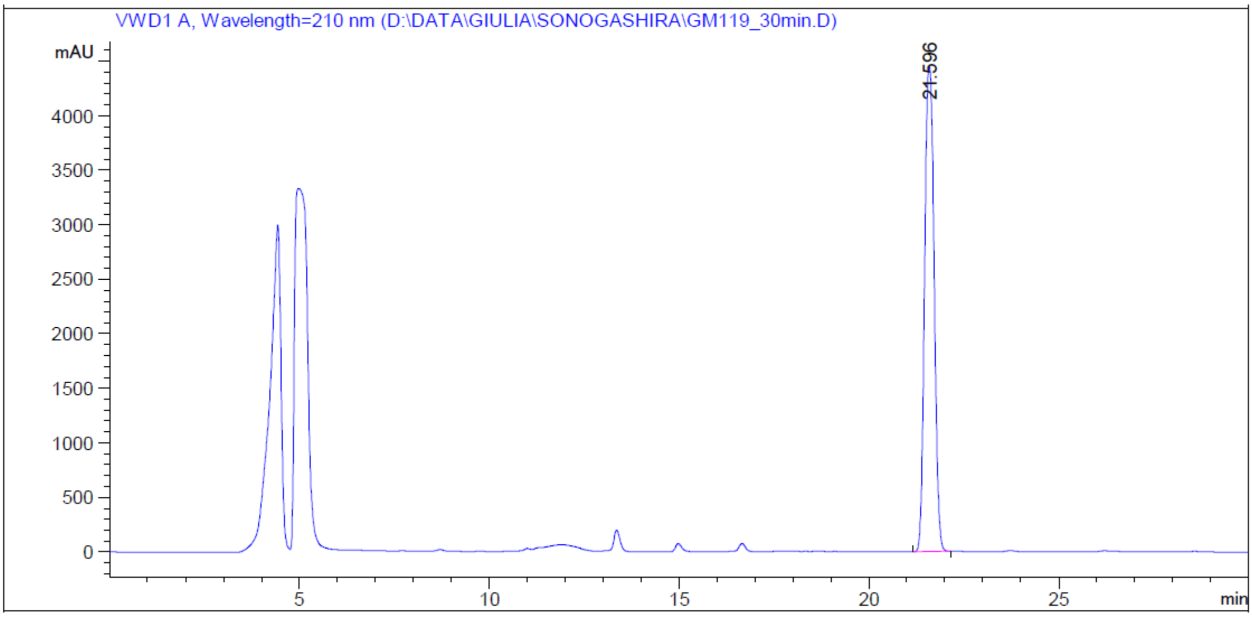

Figure S11: HPLC chromatogram of Sonogashira reaction in HEP between 4-nitroiodobenzene and phenylacetylene (**entry 1 Table 2**) after 30 minutes at 210 nm; peak at 21.596 min = product **3b**

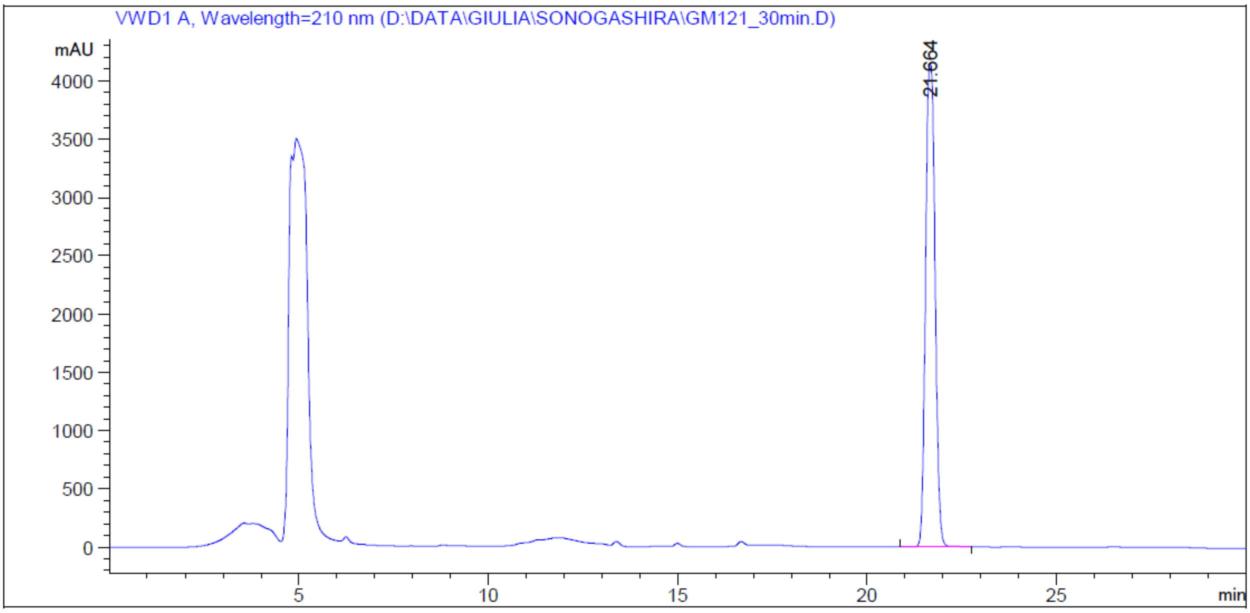

Figure S12: HPLC chromatogram of Sonogashira reaction in HEP between 3-nitroiodobenzene and phenylacetylene (**entry 2 Table 2**) after 30 minutes at 210 nm; peak at 21.664 min = product **3c**

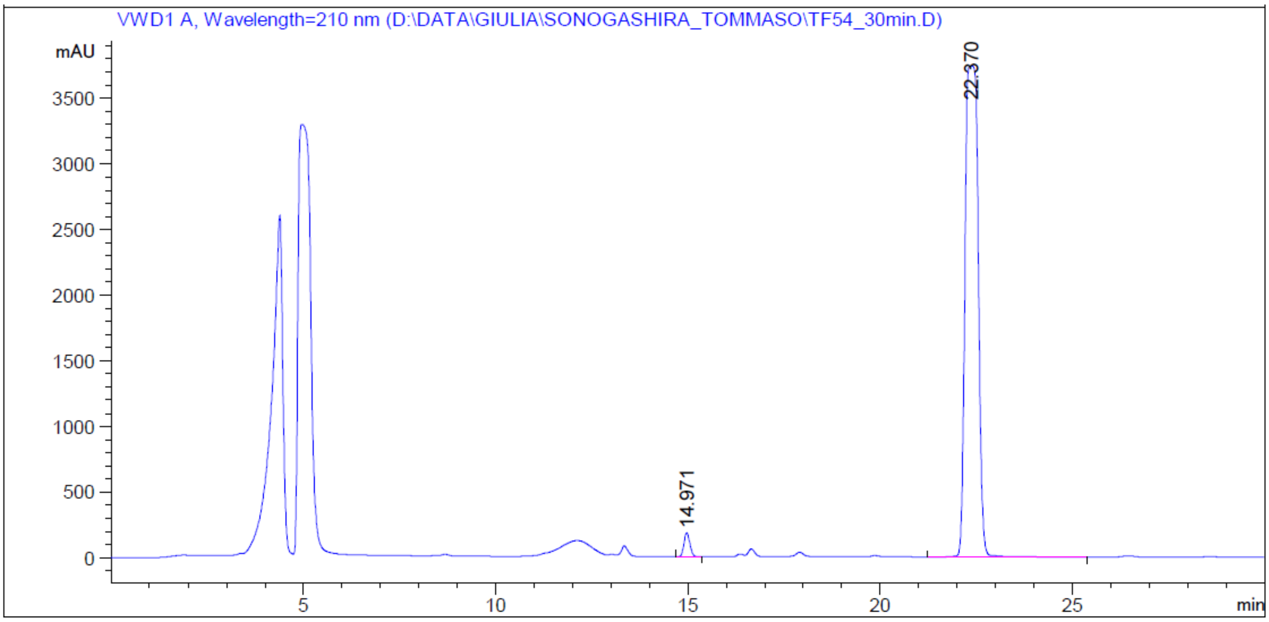

Figure S13: HPLC chromatogram of Sonogashira reaction in HEP between 3-methoxyiodobenzene and phenylacetylene (**entry 3 Table 2**) after 30 minutes at 210 nm; peak at 14.971 min = residual phenylacetylene; peak at 21.664 min = product **3d**

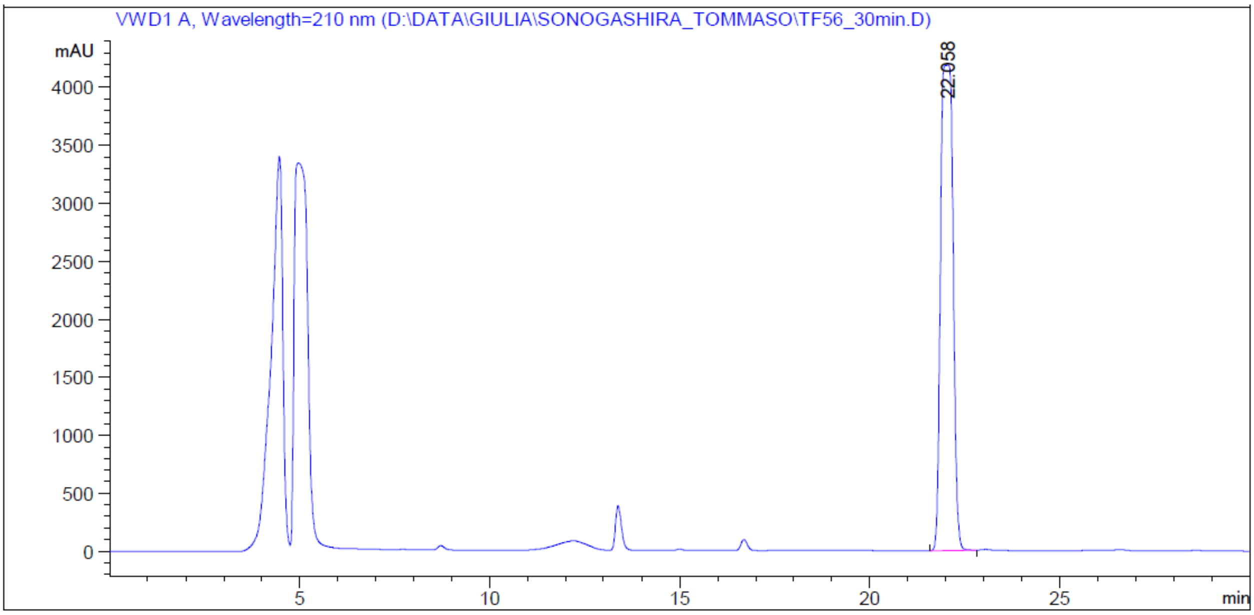

Figure S14: HPLC chromatogram of Sonogashira reaction in HEP between 4-methoxyiodobenzene and phenylacetylene (**entry 4 Table 2**) after 30 minutes at 210 nm; peak at 22.058 min = product **3e**

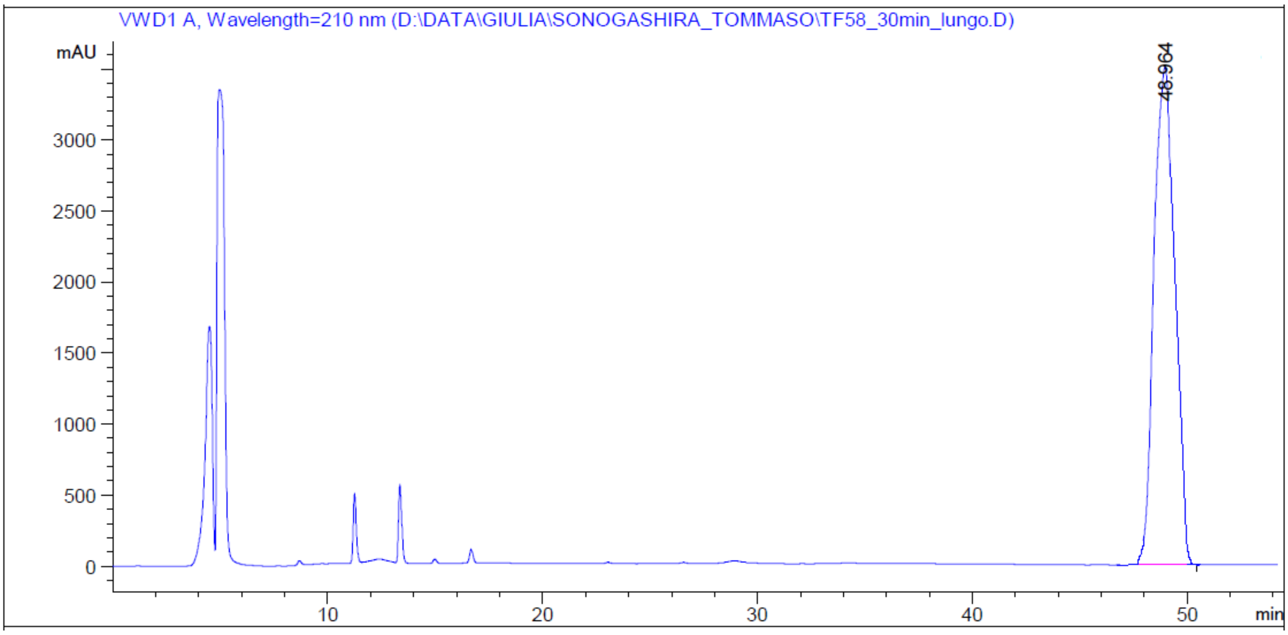

Figure S15: HPLC chromatogram of Sonogashira reaction in HEP between 3-chloroiodobenzene and phenylacetylene (**entry 5 Table 2**) after 30 minutes at 210 nm; peak at 48.964 min = product **3f**

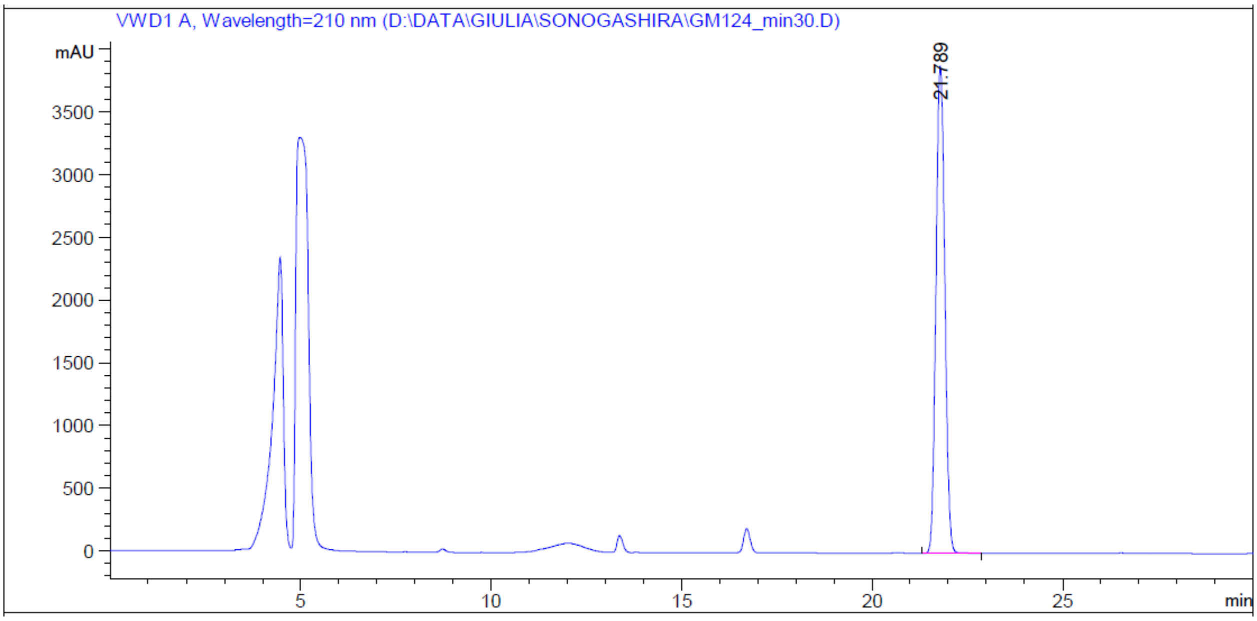

Figure S16: HPLC chromatogram of Sonogashira reaction in HEP between 2-iodothiophene and phenylacetylene (**entry 6 Table 2**) after 30 minutes at 210 nm; peak at 21.789 min = product **3g**

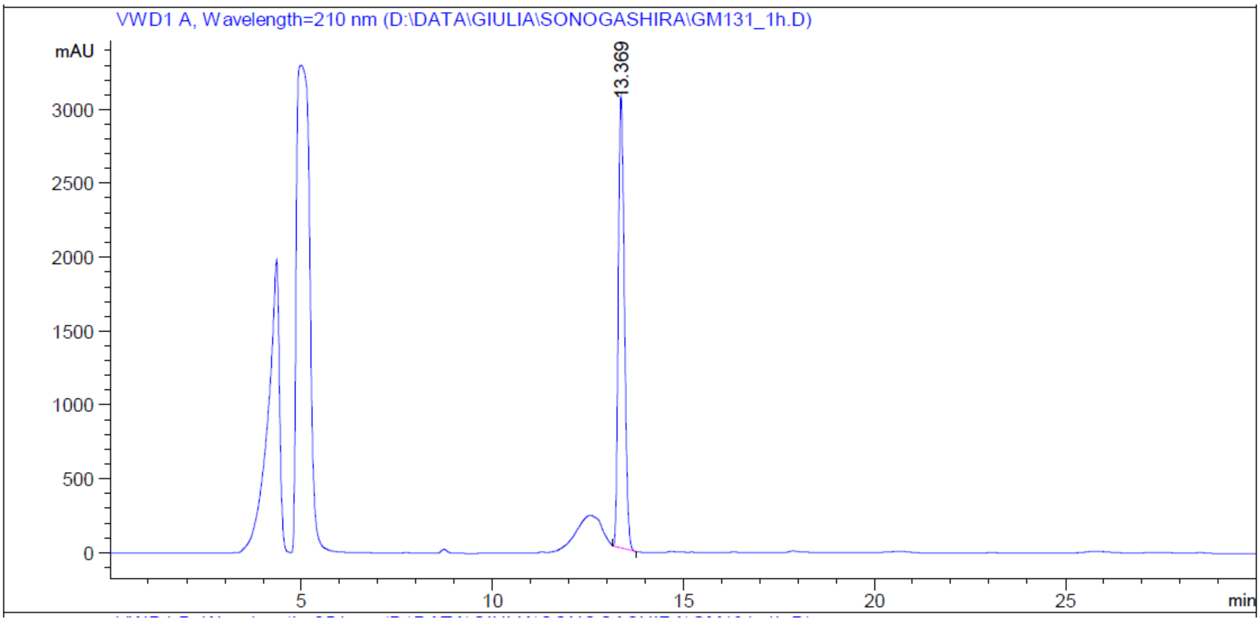

Figure S17: HPLC chromatogram of Sonogashira reaction in HEP between iodobenzene and 2-methyl-3-butyn-2-ol (**entry 7 Table 2**) after 1 hour at 210 nm; peak at 13.369 min = product **3h**

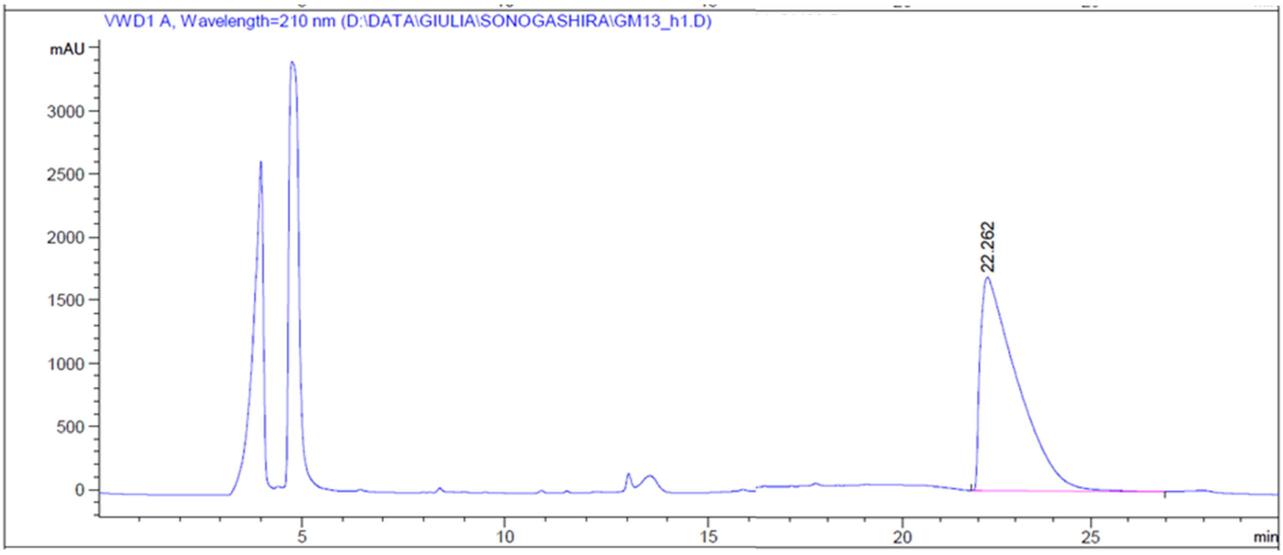

Figure S18: HPLC chromatogram of Sonogashira reaction in HEP between iodobenzene and 2-3-dimethylamino-1-propyne (**entry 8 Table 2**) after 1 hour at 210 nm; peak at 13.369 min = product **3i**

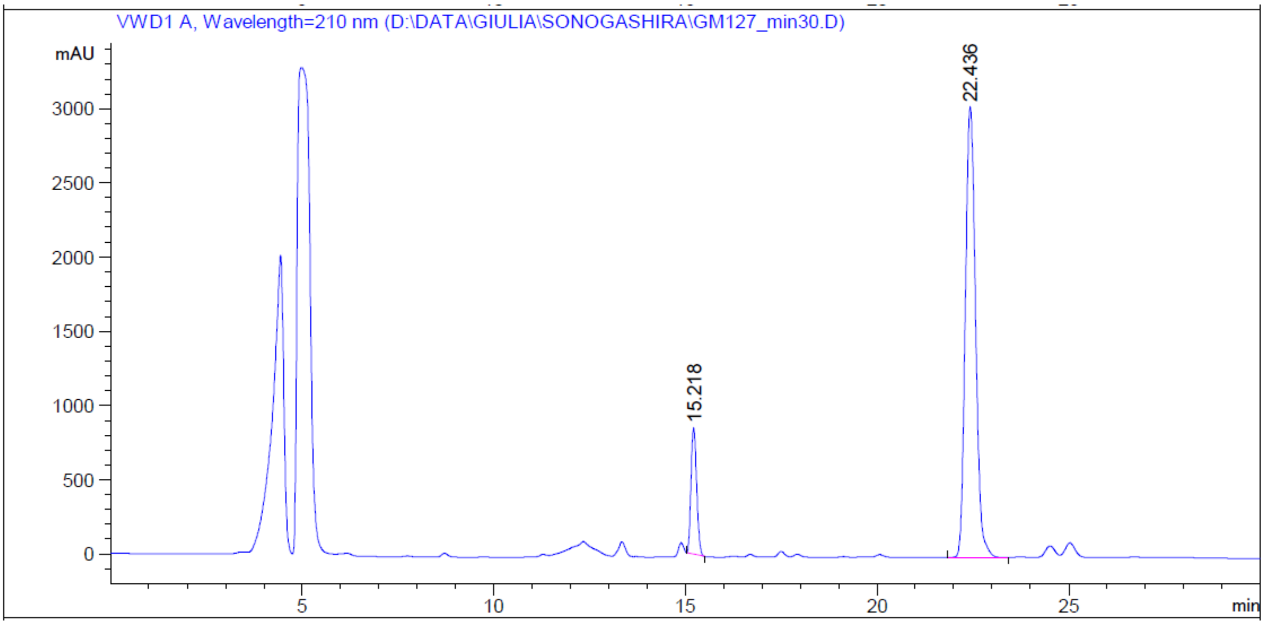

Figure S19: HPLC chromatogram of Sonogashira reaction in HEP between iodobenzene and 3-phenyl-1-propyne (**entry 9 Table 2**) after 30 min at 210 nm; peak at 15.216 min = residual 3-phenyl-1-propyne; peak at 22.436 min = product **3j**

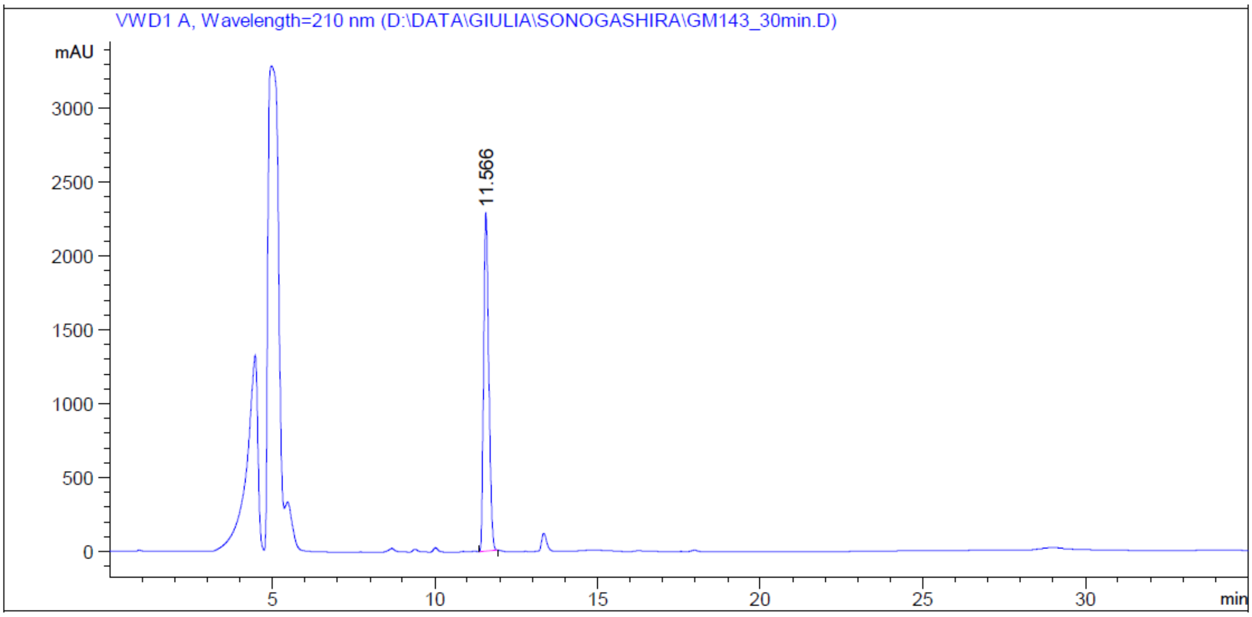

Figure S20: HPLC chromatogram of Sonogashira reaction in HEP between iodobenzene and propargyl alcohol (**entry 10 Table 2**) after 30 min at 210 nm; peak at 11.566 min = product **3k**

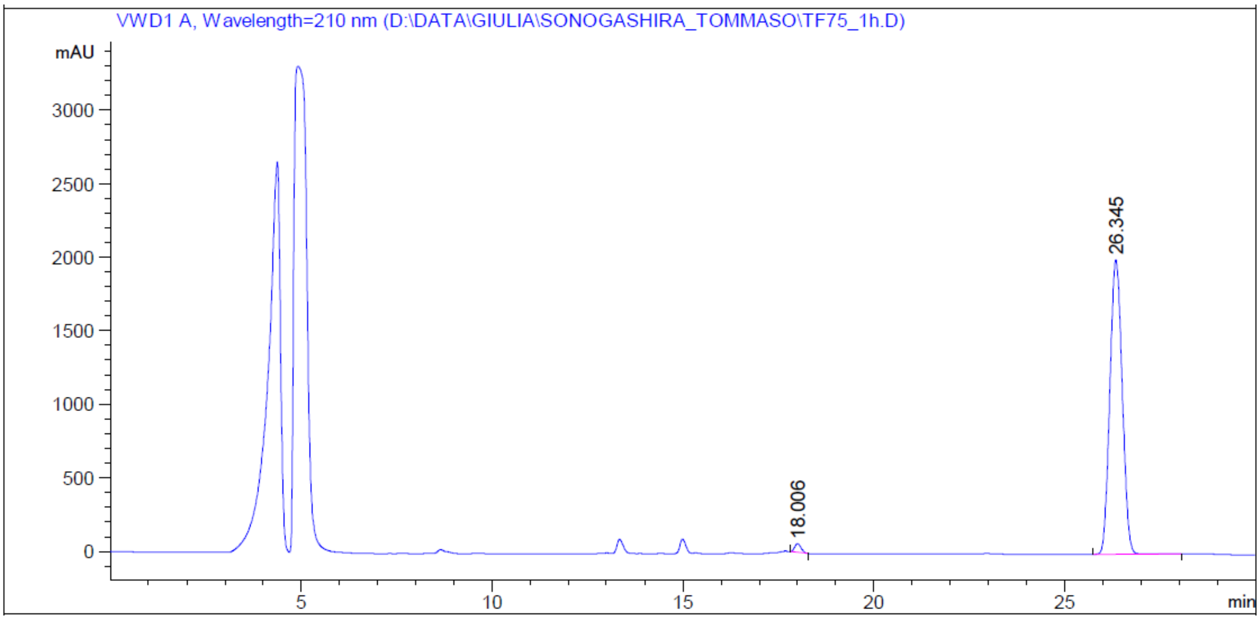

Figure S21: HPLC chromatogram of Sonogashira reaction in HEP between iodobenzene and 1-hexyne (**entry 11 Table 2**) after 1 hour at 210 nm; peak at 18.006 min = residual iodobenzene; peak at 26.345 min = product **3l**

- *Table 3 (reactions with bromoderivatives)*

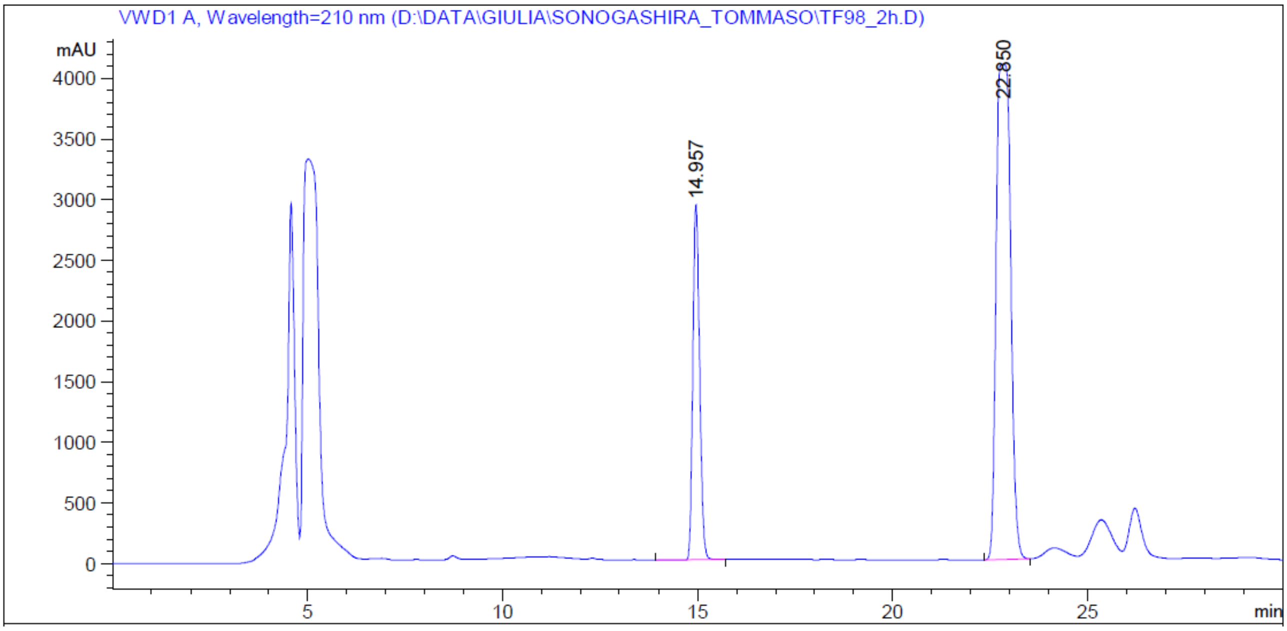

Figure S22: HPLC chromatogram of Sonogashira reaction in HEP between bromobenzene and phenylacetylene (**entry 4 Table 3**) after 2 hours at 210 nm; peak at 14.957 min = residual phenylacetylene; peak at 22.850 min = diphenylacetylene

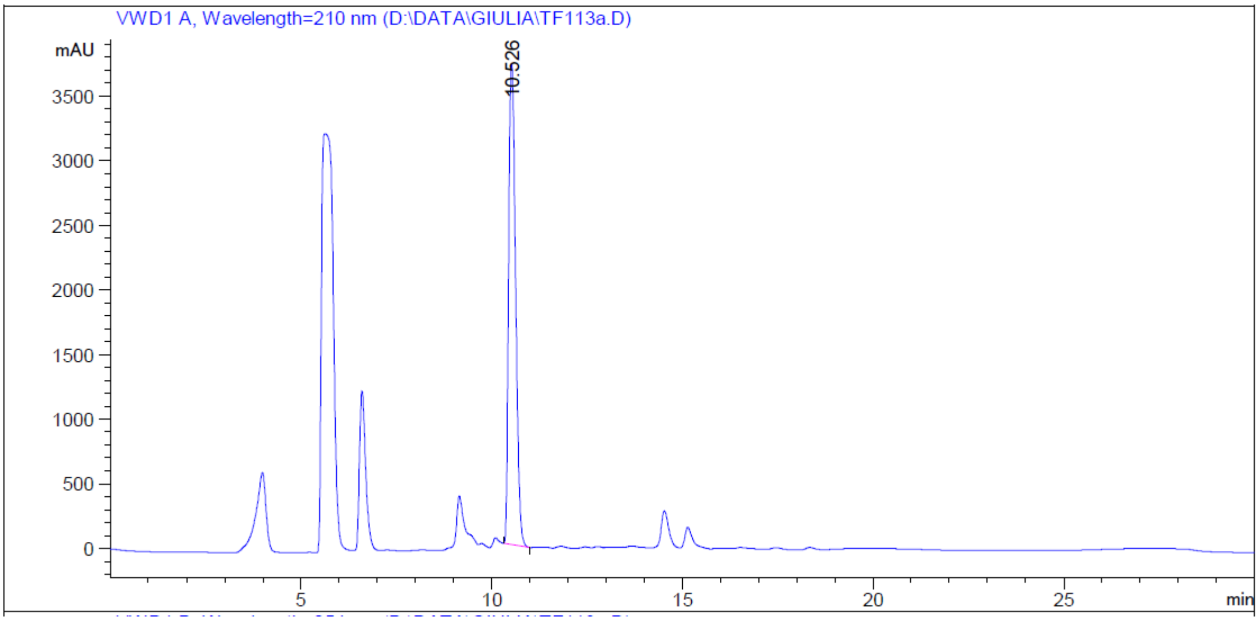

Figure S23: HPLC chromatogram of Sonogashira reaction in HEP between 3-bromoaniline and 2-methyl-3-butyn-2-ol (**entry 11 Table 3**) after 3 hours at 210 nm; peak at 10.526 min = product **5b**

2.2 Characterization data for products 3a-l

Diphenylacetylene 3a:

White solid (86 mg, 97% yield); ^{1}H and ^{13}C NMR in agreement with refs. [1], [3], [5]

^{1}H NMR (400 MHz, $CDCl_3$) δ (ppm) 7.55 (m, 4H), 7.35 (m, 6H); ^{13}C NMR (100 MHz, $CDCl_3$) δ (ppm) 131.6, 128.4, 128.2, 123.4, 89.6.

Anal. Calcd. for $C_{14}H_{10}$: C, 94.34; H, 5.66; found: C, 94.68; H, 5.65

GC-MS: rt m/z: 178 (100 %), 152 (13%), 126 (7%)

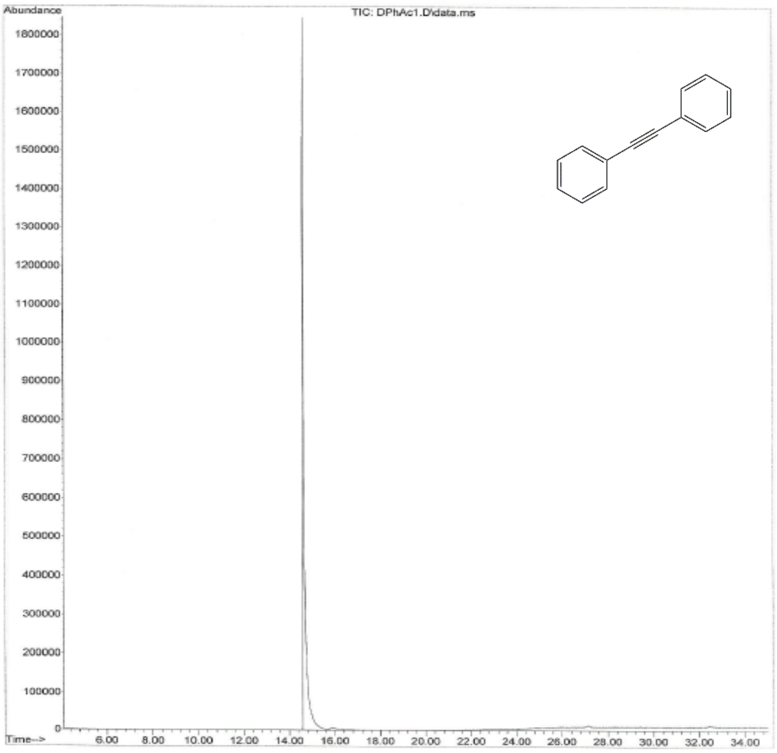

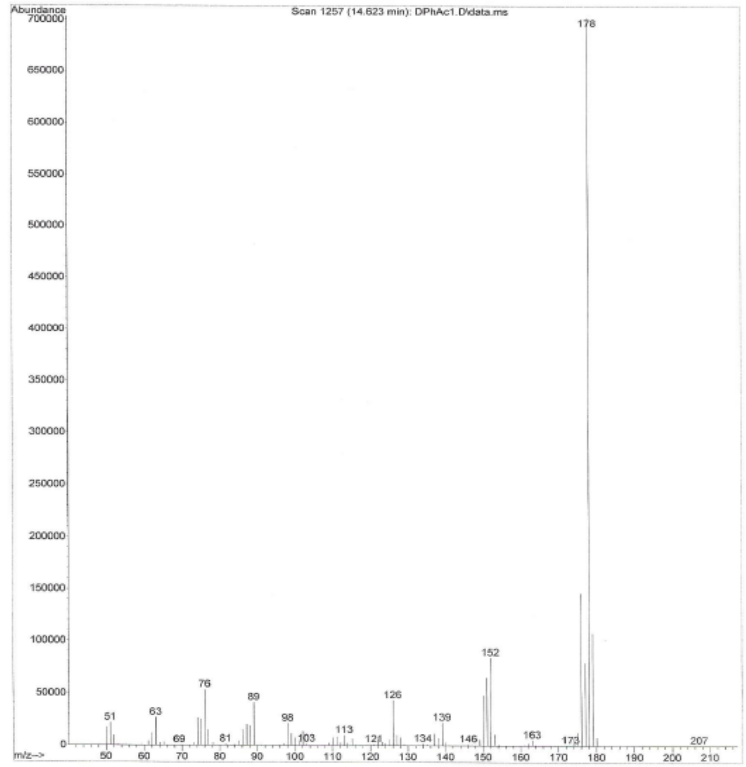

Figure S24: GC-MS spectra of product **3a**

1-nitro-4-(phenylethynyl)benzene 3b:

Yellow solid (107 mg, 96% yield); ^{1}H and ^{13}C NMR in agreement with refs. [1], [4]

^{1}H NMR (400 MHz, $CDCl_3$) δ (ppm) 8.22 (m, 2H), 7.68 (m, 2H), 7.56 (m, 2H), 7.39 (m, 3H); ^{13}C NMR (100 MHz, $CDCl_3$) δ (ppm) 146.8, 132.1, 131.8, 130.2, 129.3, 128.5, 123.5, 122.2, 94.7, 87.4.

Anal. Calcd. for $C_{14}H_9NO_2$: C, 75.33; H, 4.06; N, 6.27; found: C, 75.26; H, 4.06; N, 6.27

GC-MS: rt 19.4 min m/z: 223 (100%), 193 (33%), 176 (83%), 165 (40%), 151 (25%).

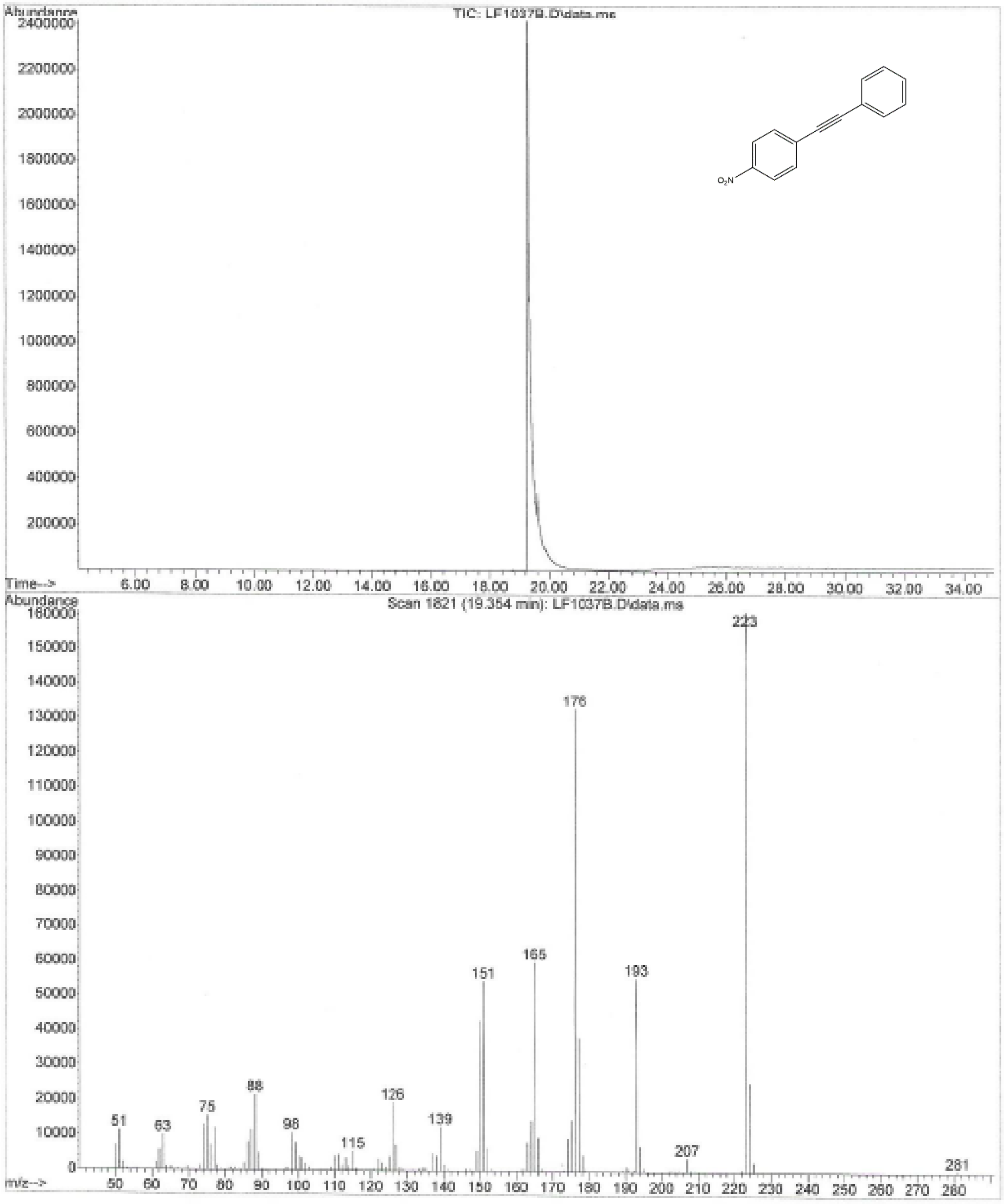

Figure S25: GC-MS spectra of product **3b**

1-nitro-3-(phenylethynyl)benzene 3c:
Yellow solid (106 mg, 95% yield); ^{1}H and ^{13}C NMR in agreement with ref. [10]
^{1}H NMR (400 MHz, $CDCl_3$) δ (ppm) 8.37 (s, 1H), 8.17 (d, 1H, J = 8.2 Hz), 7.82 (m, 1H), 7.56 (m, 3H), 7.39 (m, 3H); ^{13}C NMR (100 MHz, $CDCl_3$) δ (ppm) 147.9, 137.1, 131.6, 129.2, 129.0, 128.3, 126.2, 124.8, 122.6, 122.0, 91.7, 86.7.
Anal. Calcd. for $C_{14}H_9NO_2$: C, 75.33; H, 4.06; N, 6.27; found: C, 75.14; H, 4.06; N, 6.26
GC-MS: rt 19.1 min m/z: 223 (100%), 176 (91%), 165 (10%), 151 (33%).

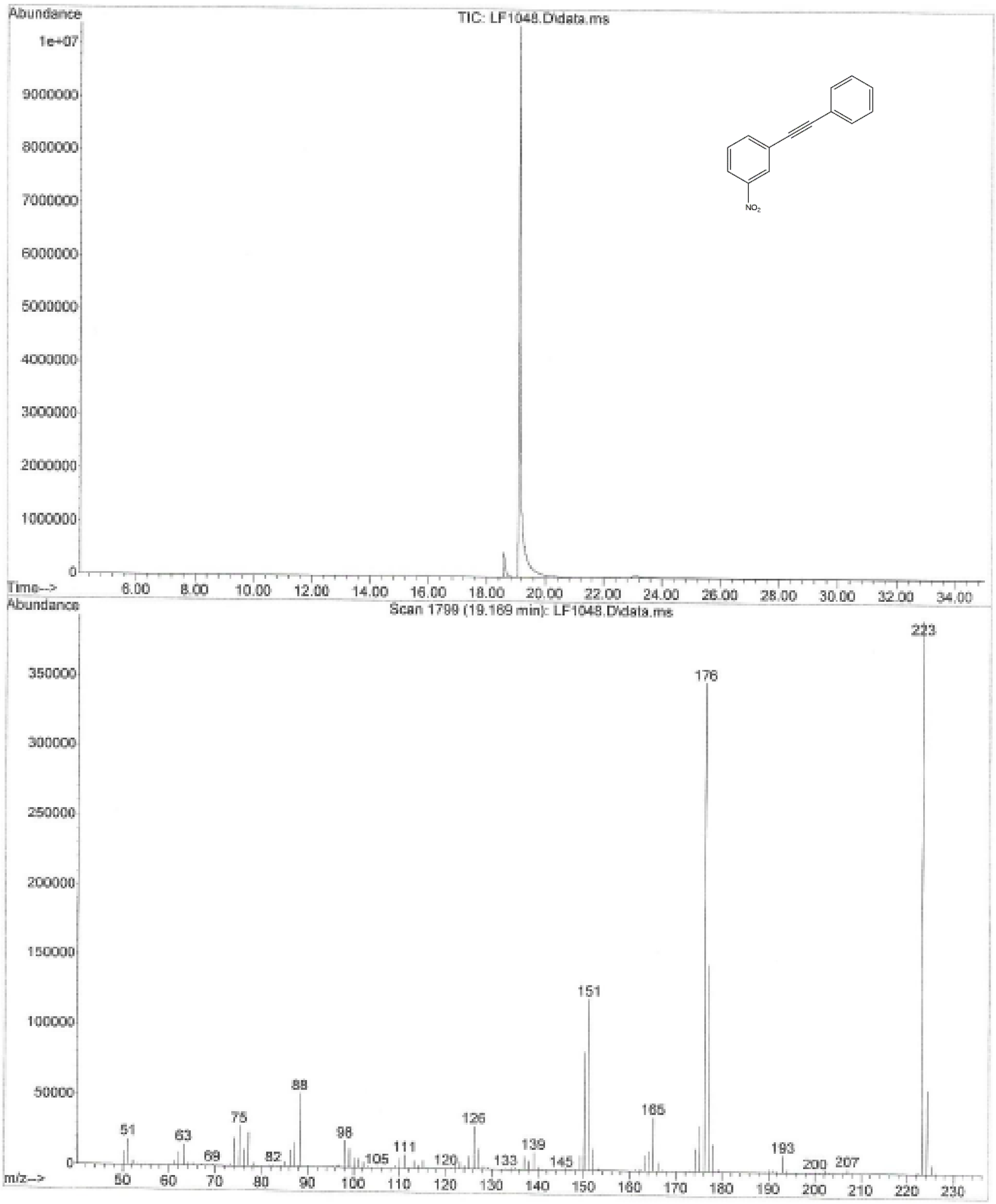

Figure S26: GC-MS spectra of product **3c**

1-methoxy-3-(phenylethynyl)benzene 3d:
White solid (102 mg, 98% yield); ^{1}H and ^{13}C NMR in agreement with ref. [1]
^{1}H NMR (400 MHz, $CDCl_3$) δ (ppm) 7.55 (m, 2H), 7.36 (m, 3H), 7.27 (m, 1H), 7.16 (m, 1H), 7.09 (m, 1H), 6.91 (m, 1H), 3.84 (s, 3H); ^{13}C NMR (100 MHz, $CDCl_3$) δ (ppm) 159.6, 131.4, 129.3, 128.4, 128.3, 124.4, 124.2, 123.0, 116.3, 114.9, 89.3, 89.1.
Anal. Calcd. for $C_{15}H_{12}O$: C, 86.51; H, 5.81; found: C, 86.72; H, 5.81
GC-MS: rt 17.5 min m/z: 208 (100), 178 (34%), 165 (40%)

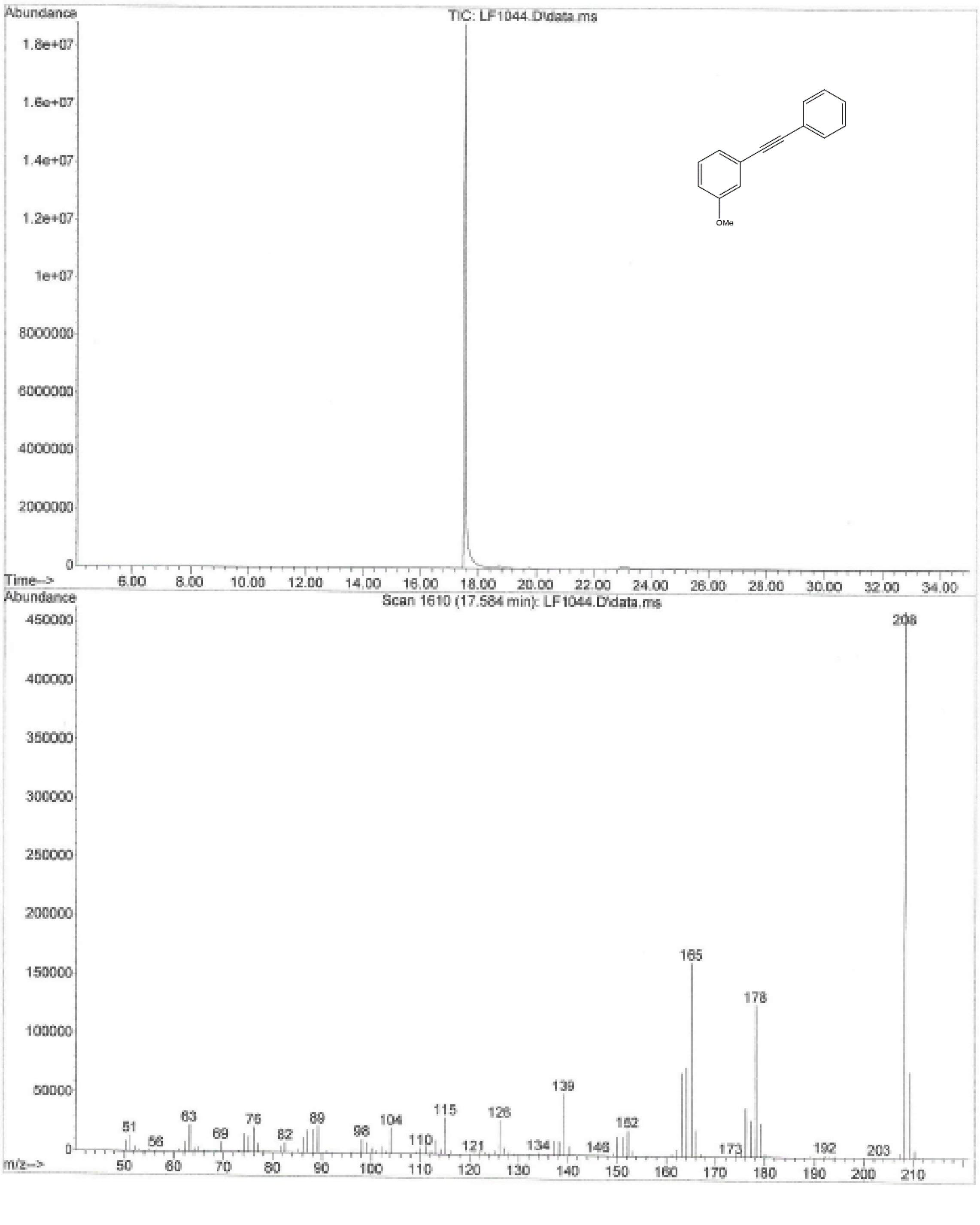

Figure S27: GC-MS spectra of product **3d**

1-methoxy-4-(phenylethynyl)benzene 3e:
White solid (101 mg, 98% yield); ^{1}H and ^{13}C NMR in agreement with refs. [3], [4], [5]
^{1}H NMR (400 MHz, $CDCl_3$) δ (ppm) 7.50 (m, 4H), 7.34 (m, 3H), 6.88 (d, J = 8.4 Hz, 2H), 3.83 (s, 3H); ^{13}C NMR (100 MHz, $CDCl_3$) δ (ppm) 159.6, 133.1, 131.7, 128.3, 127.8, 123.6, 115.2, 114.0, 89.8, 88.2, 55.3.
Anal. Calcd. for $C_{15}H_{12}O$: C, 86.51; H, 5.81; found: C, 86.72; H, 5.81
GC-MS: rt 17.8 min m/z: 208 (100%), 193 (55%), 165 (58%)

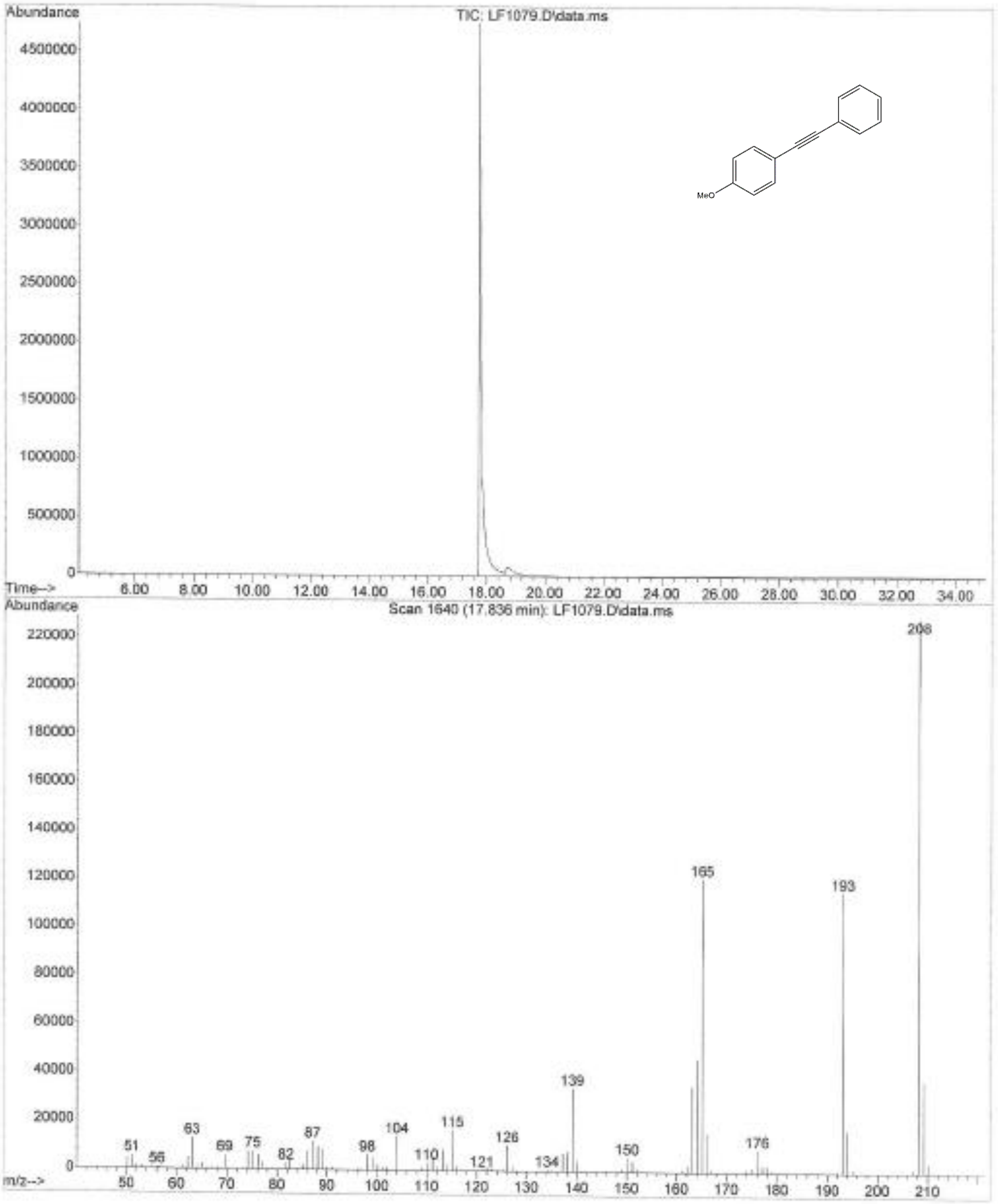

Figure S28: GC-MS spectra of product **3e**

1-chloro-3-(phenylethynyl)benzene 3f:
Colourless oil (101 mg, 95% yield); ^{1}H and ^{13}C NMR in agreement with refs. [1], [3]
^{1}H NMR (400 MHz, $CDCl_3$) δ (ppm) 7.55 (m, 3H), 7.42 (m, 1H), 7.37 (m, 3H), 7.30 (m, 2H); ^{13}C NMR (100 MHz, $CDCl_3$) δ (ppm) 134.2, 131.6, 131.3, 129.7, 129.5, 128.9, 128.6, 128.4, 125.0, 122.9, 91.0, 87.8.
Anal. Calcd. for $C_{14}H_9Cl$: C, 79.07; H, 4.27; found: C, 78.83; H, 4.27
GC-MS: rt 16.7 m/z: 212 (100), 176 (45%), 151 (17%)

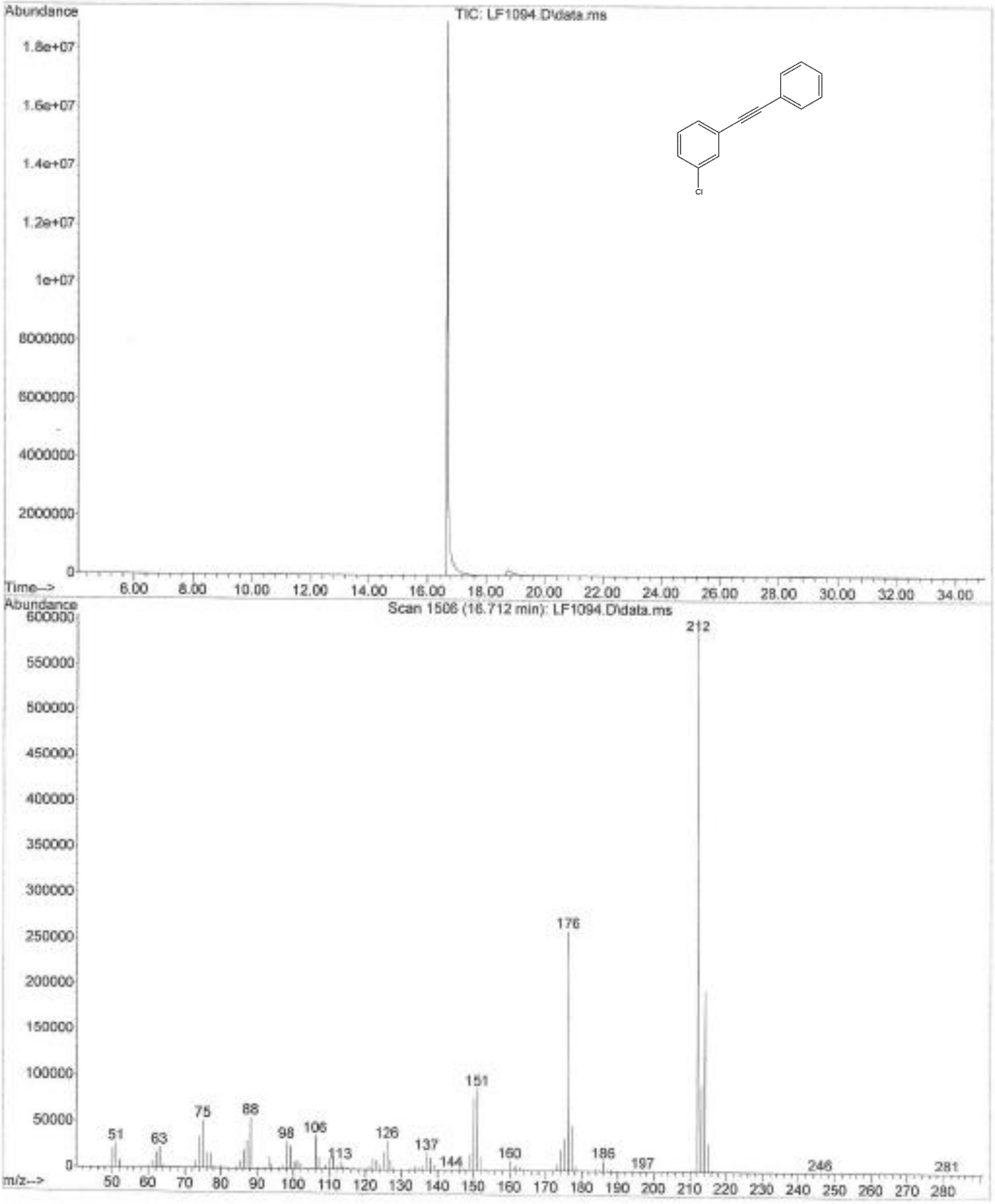

Figure S29: GC-MS spectra of product **3f**

2-(phenylethynyl)thiophene 3g:
White solid (90 mg, 98% yield); ^{1}H and ^{13}C NMR in agreement with refs. [1], [3]
^{1}H NMR (400 MHz, $CDCl_3$) δ (ppm) 7.54 (m, 2H), 7.37 (m, 3H), 7.31 (m, 2H), 7.02 (m, 1H); ^{13}C NMR (100 MHz, $CDCl_3$) δ (ppm) 132.0, 131.1, 128.4, 128.3, 127.2, 127.0, 123.1, 123.0, 93.5, 82.4.
Anal. Calcd. for $C_{12}H_8S$: C, 78.22; H, 4.38; found: C, 78.31; H, 4.39
GC-MS: rt 15.0 m/z: 184 (100%), 152 (13%), 139 (23%)

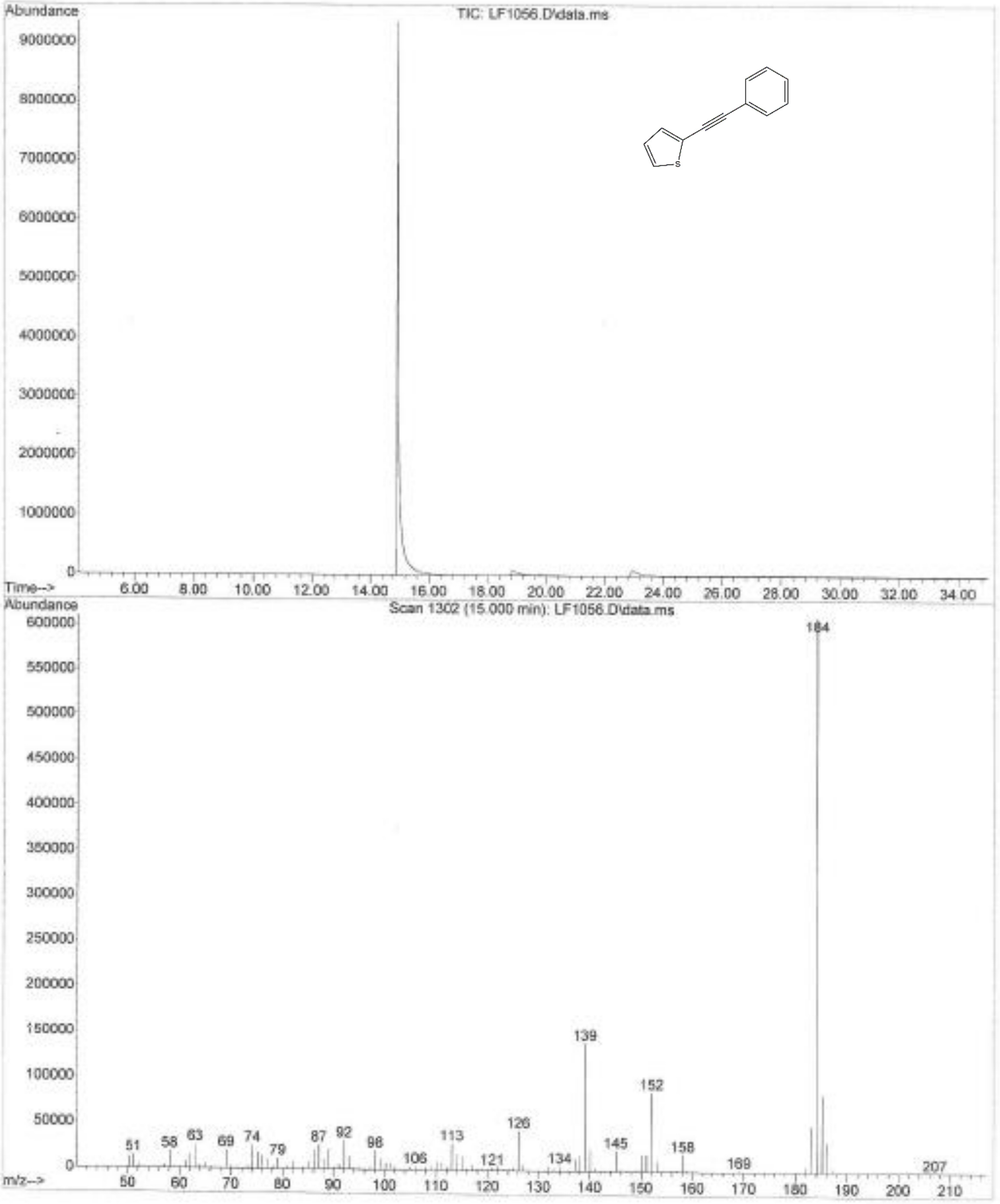

Figure S30: GC-MS spectra of product **3g**

2-methyl-4-phenylbut-3-yn-2-ol 3h:

Yellow oil (75 mg, 94% yield); ^{1}H and ^{13}C NMR in agreement with ref. [6]

^{1}H NMR (400 MHz, $CDCl_3$) δ (ppm) 7.40 (m, 2H), 7.30 (m, 3H), 2.01 (bs, 1H), 1.62 (s, 6H).); ^{13}C NMR (100 MHz, $CDCl_3$) δ (ppm) 131.6, 128.3, 128.2, 122.7, 93.9, 82.0, 65.6, 31.4.

Anal. Calcd. for $C_{11}H_{12}O$: C, 82.46; H, 7.55; found: C, 82.78; H, 7.54

GC-MS: rt 10.1, m/z: 160 (22%), 145 (100%), 129 (10%), 115 (20%)

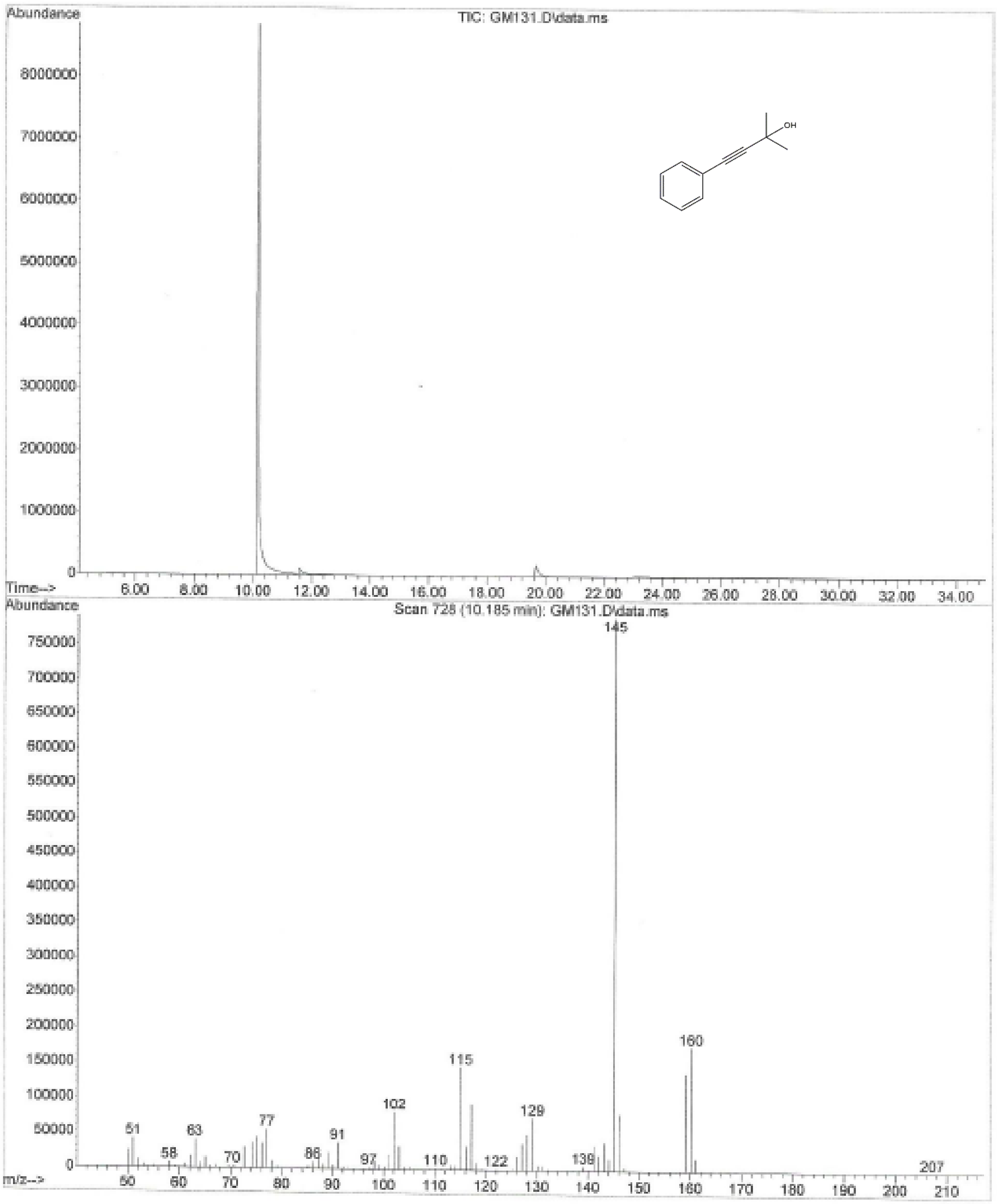

Figure S31: GC-MS spectra of product **3h**

N,N-dimethyl-3-phenylprop-2-yn-1-amine 3i:
Colourless liquid (76 mg, 96% yield); ^{1}H and ^{13}C NMR in agreement with ref. [4]
^{1}H NMR (400 MHz, $CDCl_3$) δ (ppm) 7.44 (m, 2H), 7.31 (m, 3H), 3.50 (s, 2H), 2.40 (s, 6H). ^{13}C NMR (100 MHz, $CDCl_3$) δ (ppm) 131.8, 128.3, 128.2, 128.1, 85.6, 84.3, 49.0, 44.3.
Anal. Calcd. for $C_{11}H_{13}N$: C, 82.97; H, 8.23; N, 8.80; found: C, 82.99; H, 8.22; N, 8.77;
GC-MS: rt 10.9 m/z: 159 (75%), 143 (9%), 115 (100%), 89 (15%), 82 (15%).

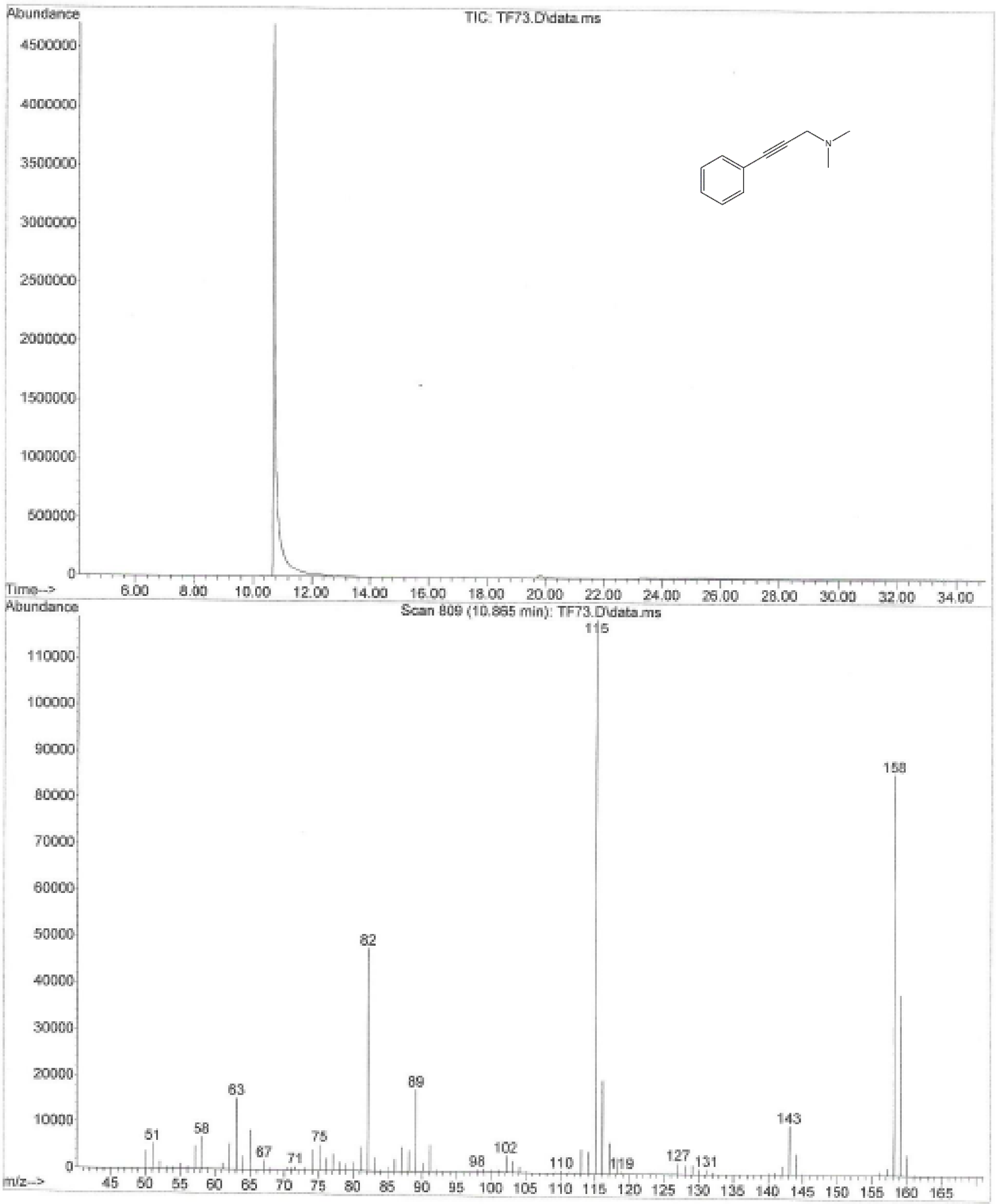

Figure S32: GC-MS spectra of product **3i**

Prop-1-yne-1,3-diyldibenzene 3j:
Yellow oil (94 mg, 98% yield); ^{1}H and ^{13}C NMR in agreement with ref. [5]
^{1}H NMR (400 MHz, $CDCl_3$) δ (ppm) 7.45 (m, 4H), 7.31 (m, 6H), 3.84 (s, 2H);^{13}C NMR (100 MHz, $CDCl_3$) δ (ppm) 136.8, 131.6, 128.7, 128.6, 128.4,128.2, 127.9, 126.4, 123.7, 88.0, 82.7, 25.4.
Anal. Calcd. for $C_{15}H_{12}$: C, 93.71; H, 6.29; found: C, 93.81; H, 6.27
GC-MS: rt 15.8 m/z: 192 (100%), 165 (30%), 115 (15%)

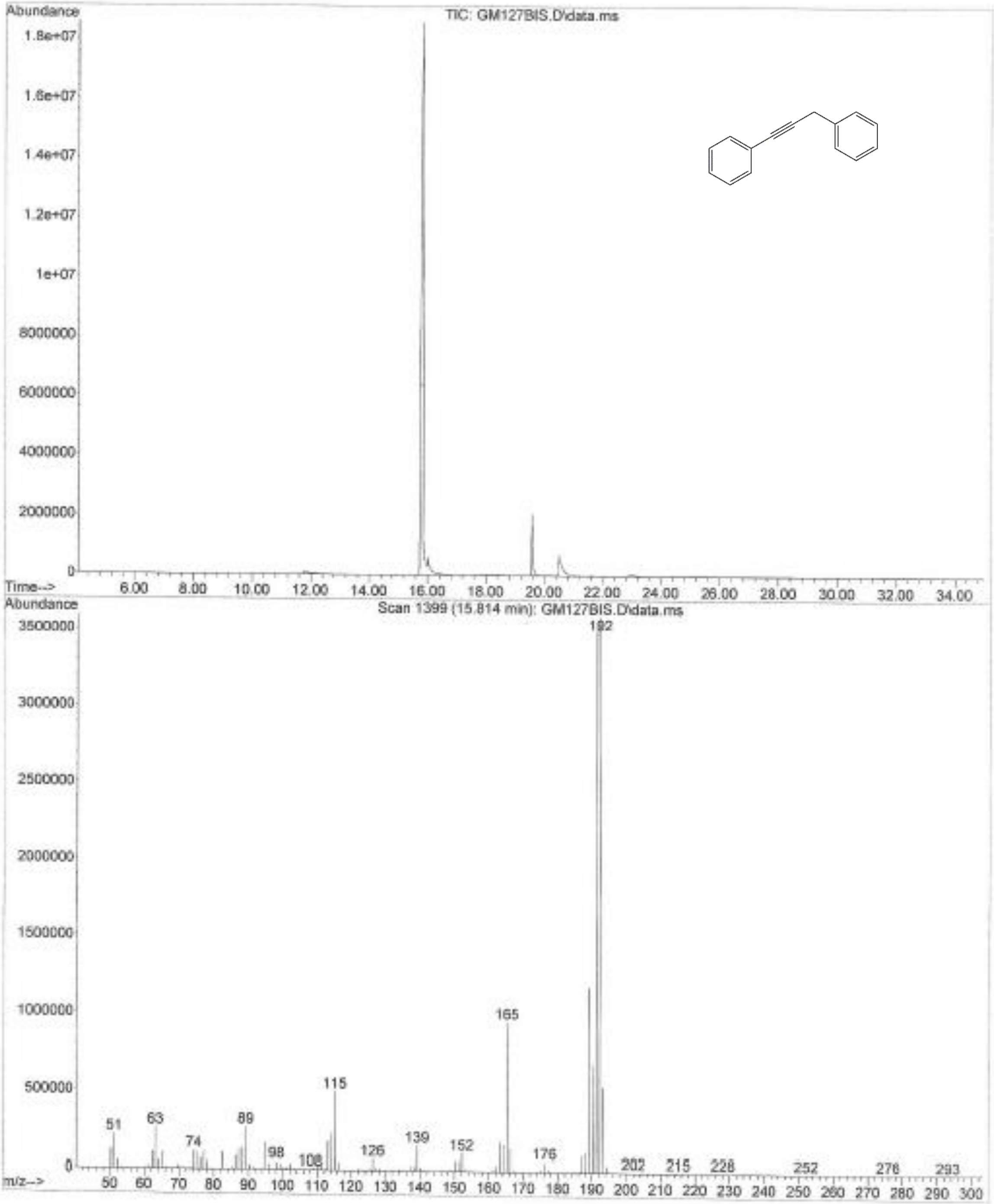

Figure S33: GC-MS spectra of product **3j**

3-phenylprop-2-yn-1-ol 3k:
Yellow oil (63 mg, 95% yield); ^{1}H and ^{13}C NMR in agreement with refs. [4], [8]
^{1}H NMR (400 MHz, $CDCl_3$) δ (ppm) 7.43 (m, 2H), 7.33 (m, 3H), 4.50 (s, 2H), 1.84 (bs, 1H); ^{13}C NMR (100 MHz, $CDCl_3$) δ (ppm) 131.8, 128.7, 128.4, 122.7, 87.4, 85.6, 51.4.
Anal. Calcd. for C_9H_8O: C, 81.79; H, 6.10; found: C, 81.97; H, 6.09
GC-MS: rt 10.4 m/z: 131 (100), 115 (20%), 103 (60%), 77 (53%),

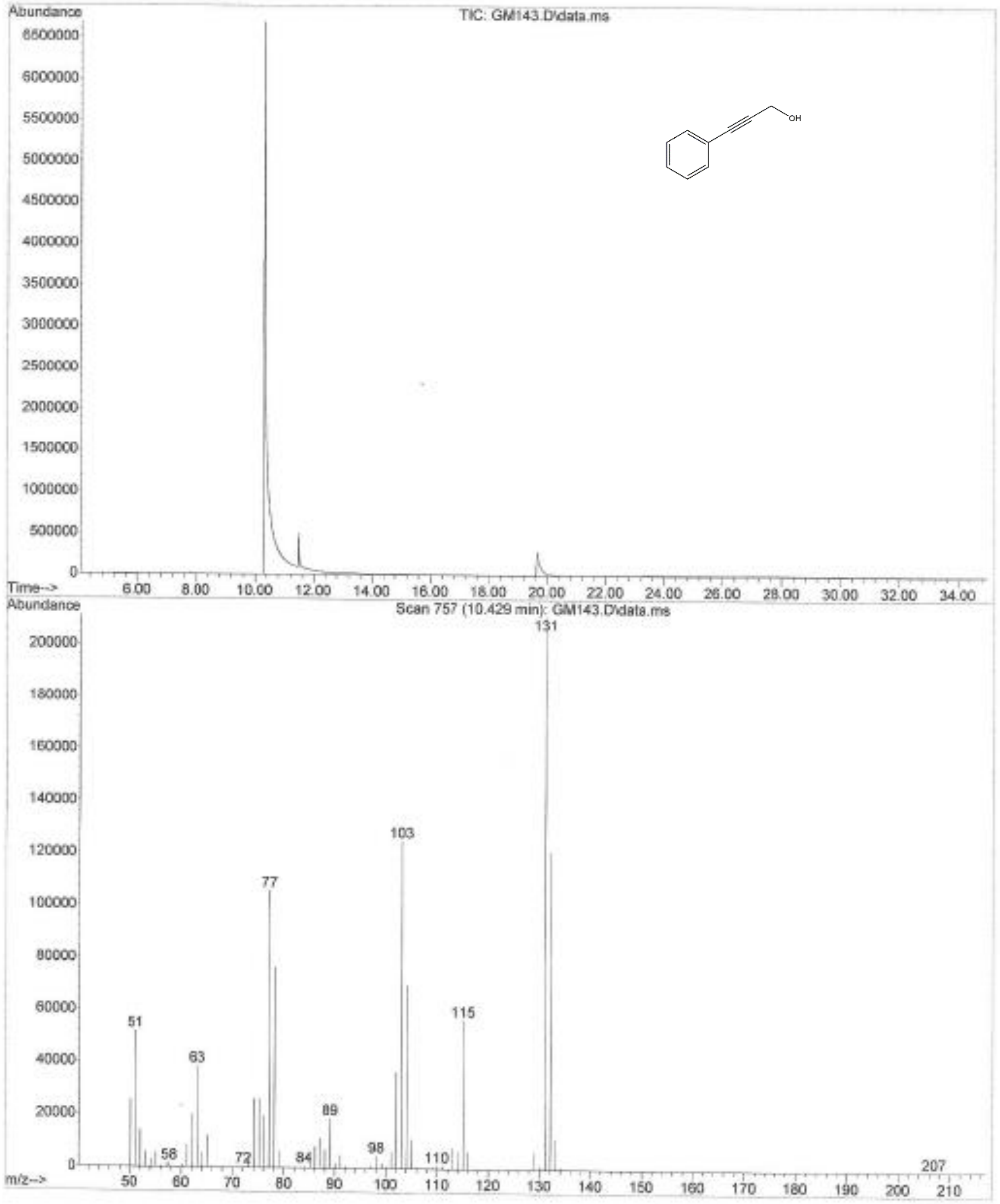

Figure S34: GC-MS spectra of product **3k**

Hex-1-yn-1-ylbenzene 3l:
Yellow oil (75 mg, 95% yield); ^{1}H and ^{13}C NMR in agreement with refs. [2], [9]
^{1}H NMR (400 MHz, $CDCl_3$) δ (ppm) 7.39 (m, 2H), 7.26 (m, 3H), 2.41 (t, *J* = 7.0 Hz, 2H), 1.59 (m, 2H), 1.49 (m, 2H), 0.95 (t, *J* = 7.3 Hz, 3H); ^{13}C NMR (100 MHz, $CDCl_3$) δ (ppm) 131.5, 128.2, 127.8, 123.0, 89.9, 80.6, 30.6, 22.2, 20.9, 13.4.
Anal. Calcd. for $C_{12}H_{14}$: C, 91.08; H, 8.92; found: C, 91.22; H, 8.92
GC-MS: rt m/z: 158 (26%), 143 (41%), 129 (80%), 115 (100%)

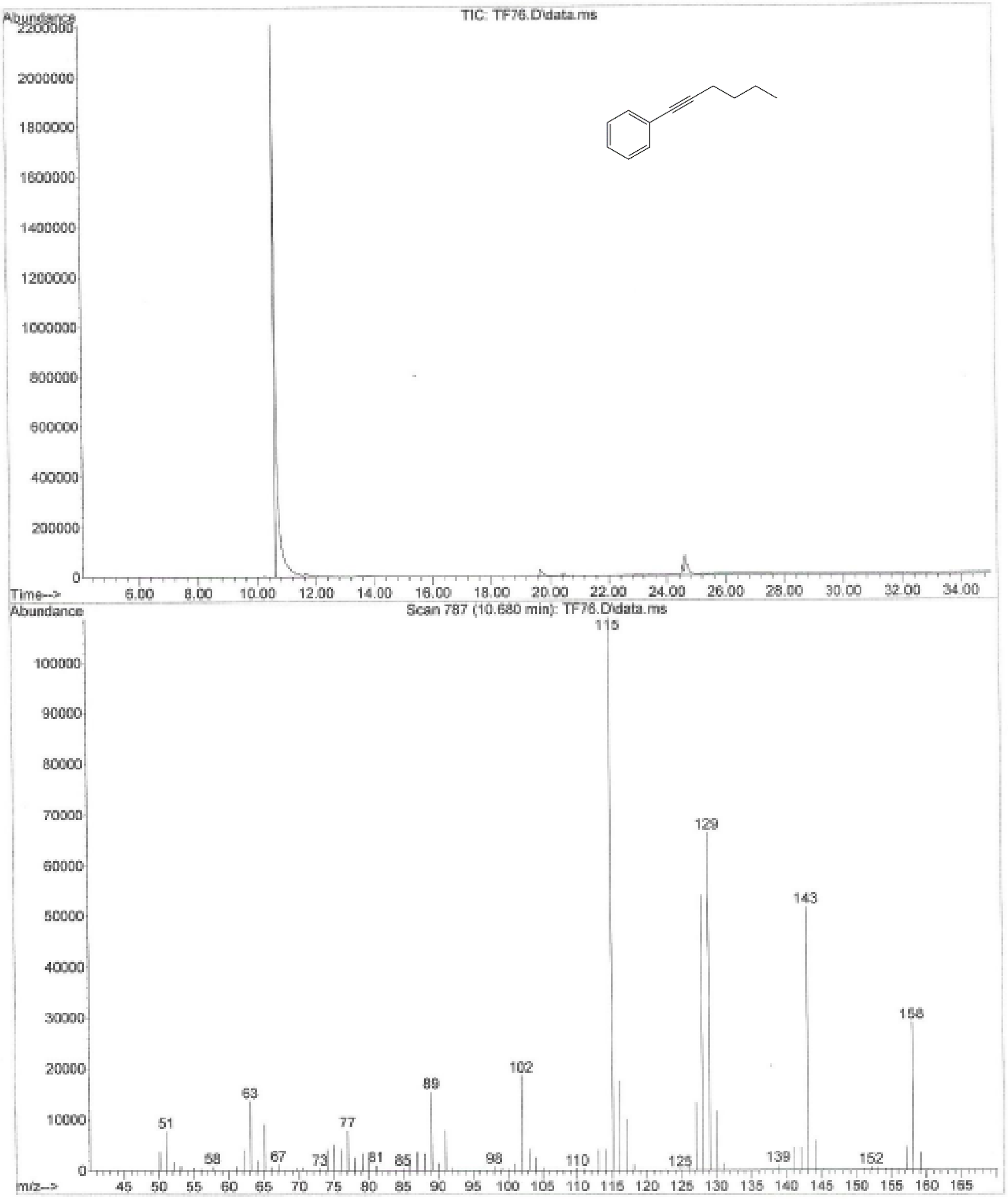

Figure S35: GC-MS spectra of product **3l**

4-(3-aminophenyl)-2-methylbut-3-yn-2-ol 5b:
GC-MS: rt 14.4 m/z: 175 (65%), 160 (100%), 144 (9%), 132 (14%), 118 (31%)
The crude extract was directly converted into **6b** (see next page)

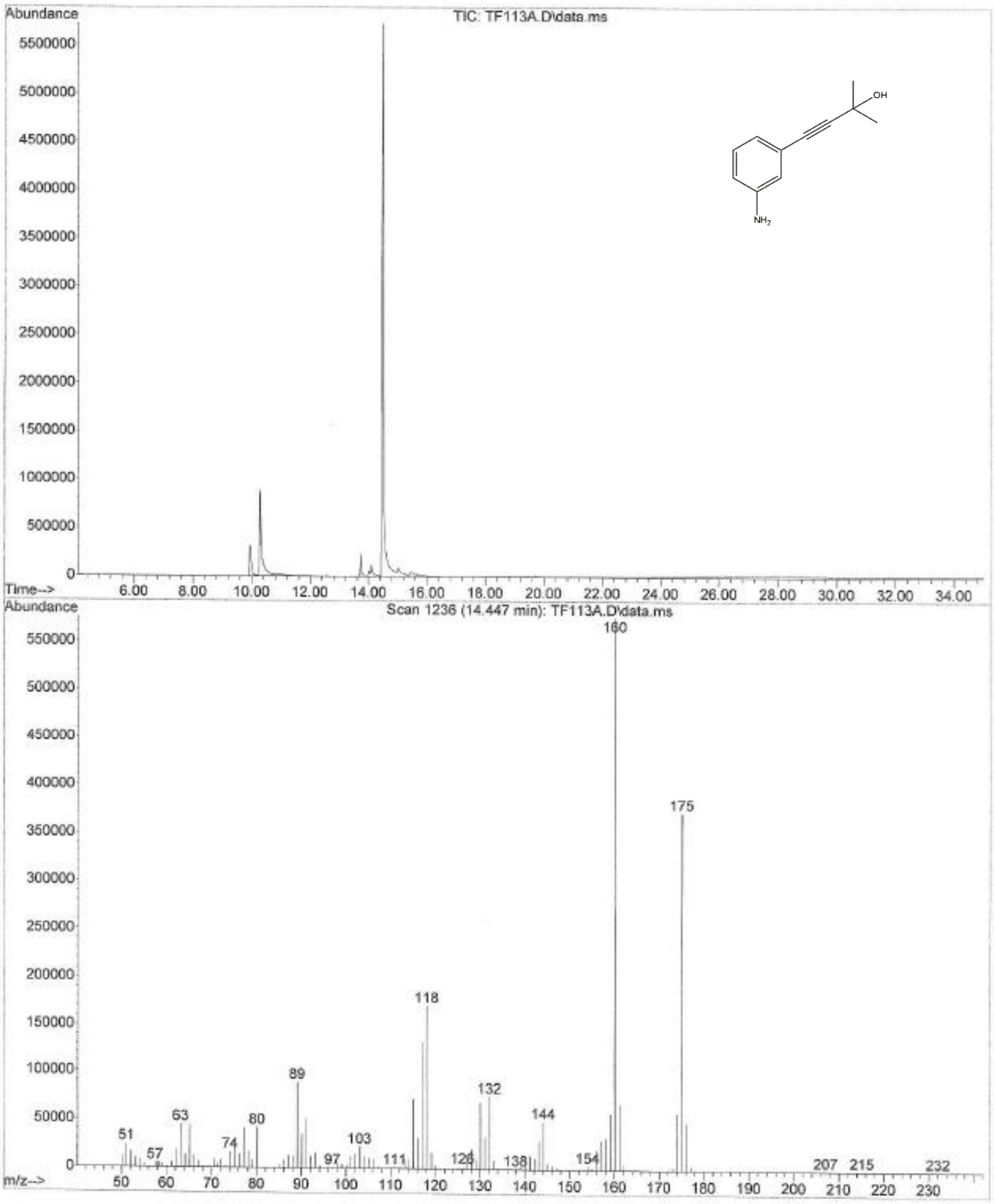

Figure S36: GC-MS spectra of crude product **5b** (peaks at 9.9 and 10.3 min correspond to homocoupling side products)

Characterization of the deprotected 1-amino-3-ethynyl-benzene **6b** (50 mg, 86%) is in agreement with ref. [11].

^{1}H NMR (400 MHz, DMSO-d_6) δ (ppm) 7.00 (m, 1H), 6.60 (m, 3H), 5.22 (bs, 2H), 3.97 (s, 1H); ^{13}C NMR (100 MHz, DMSO-d_6) δ (ppm) 148.8, 129.2, 122.0, 119.1, 116.5, 114.7, 84.4, 79.1.

Anal. Calcd. for C_8H_7N: C, 82.02; H, 6.02; N, 11.96; found: C, 82.19; H, 6.00; N, 11.96;

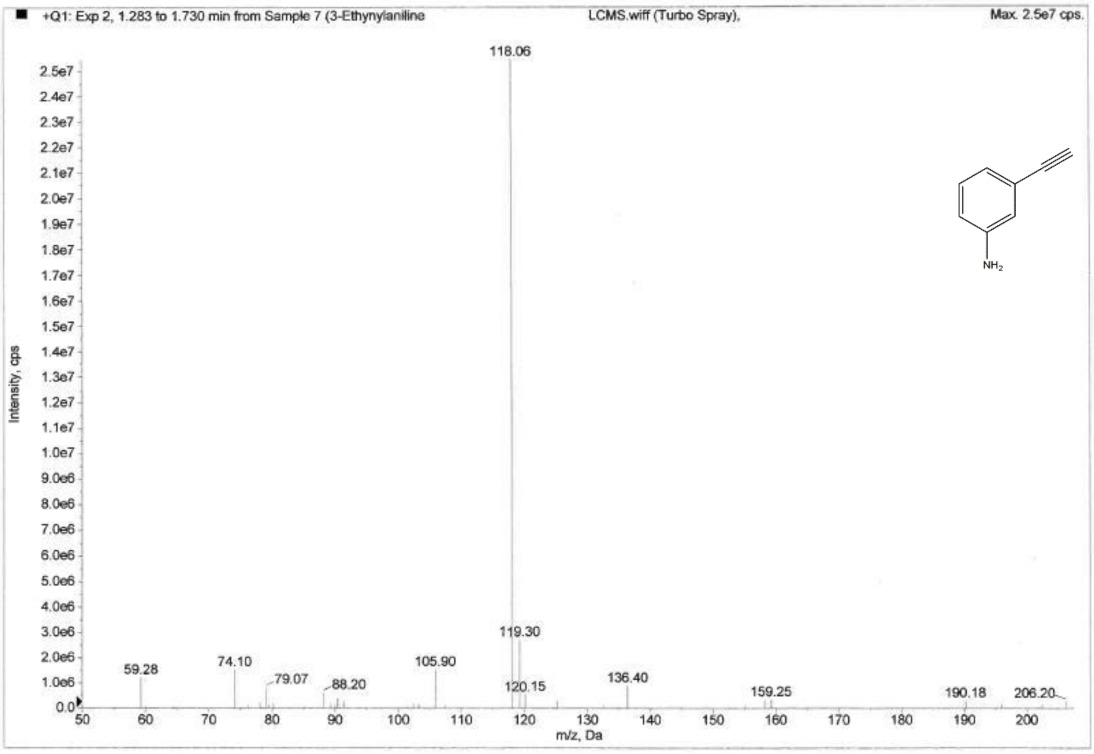

Figure S37: LC-MS spectra (ESI) of 1-amino-3-ethynyl-benzene **6b**

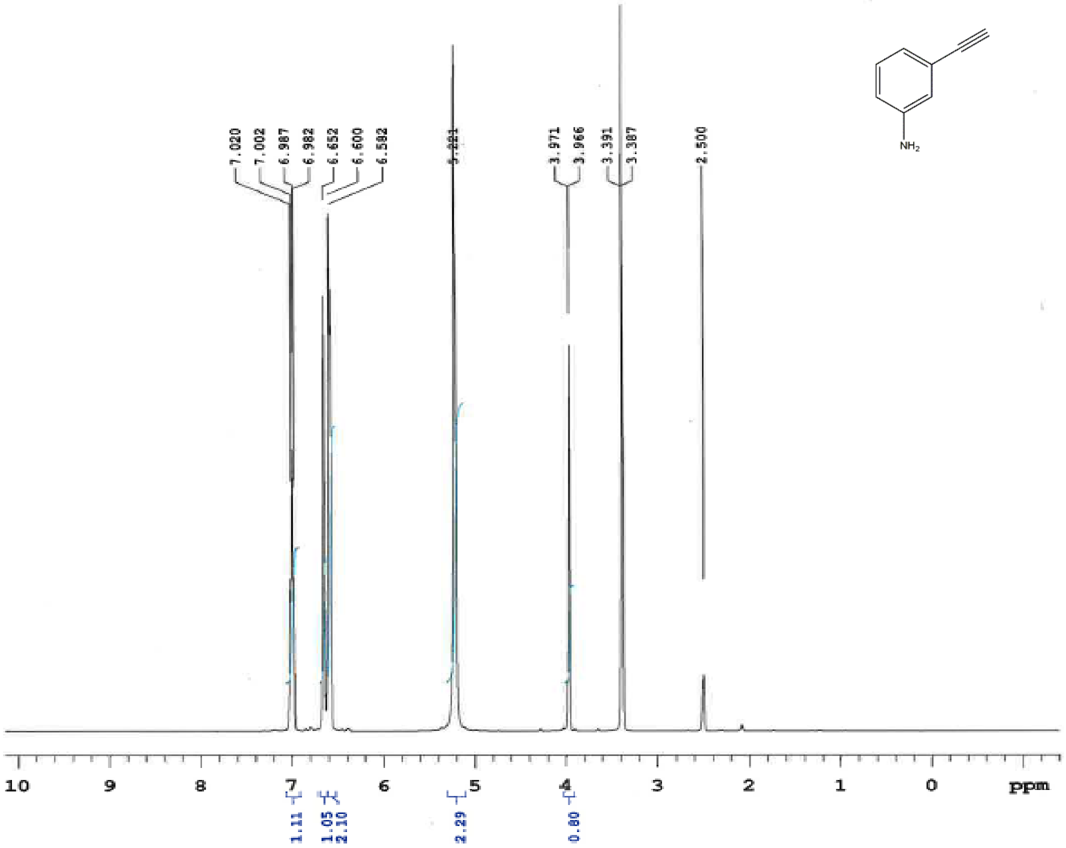

Figure S38: ^{1}H NMR spectrum (DMSO-d6, 400 MHz) of 1-amino-3-ethynyl-benzene **6b**

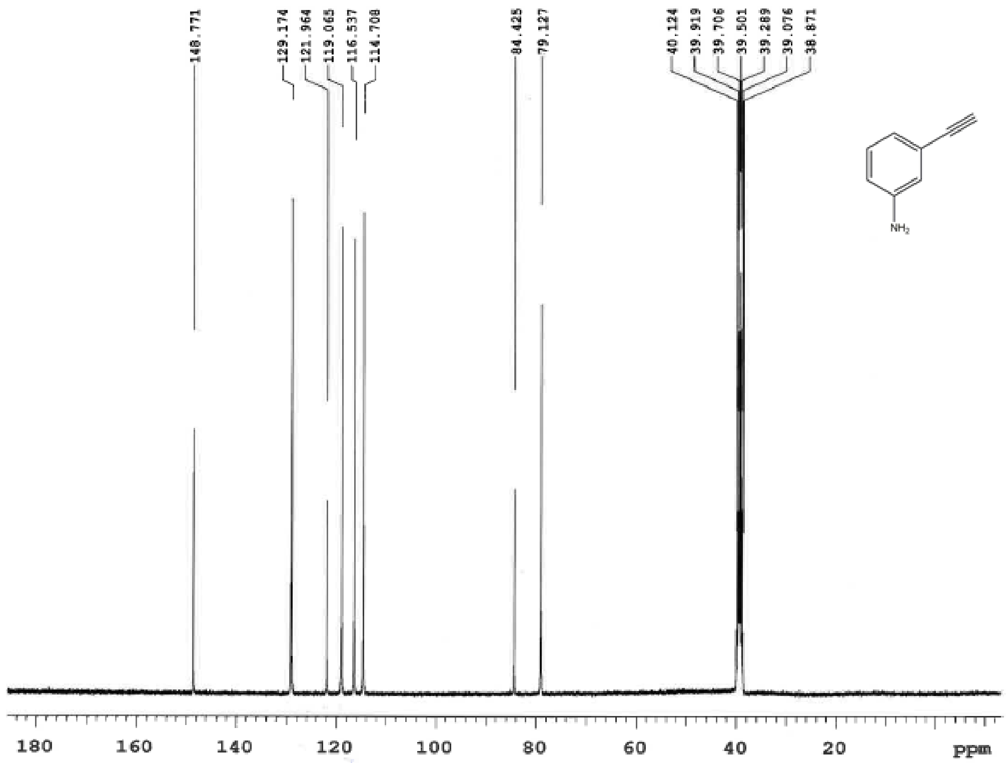

Figure S39: ^{13}C NMR spectrum (DMSO-d6, 400 MHz) of 1-amino-3-ethynyl-benzene **6b**

2.3 HPLC-UV chromatograms of commercial reagents:

- *Table 1:*

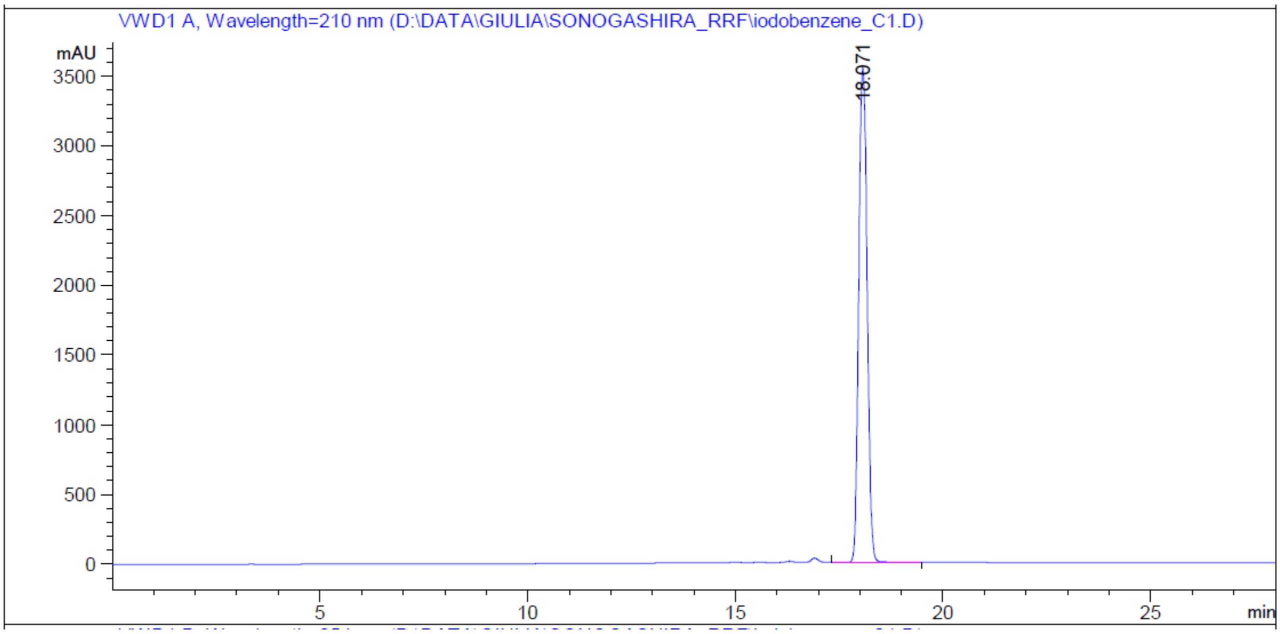

Figure S40: HPLC chromatogram of iodobenzene **1a** at 210 nm

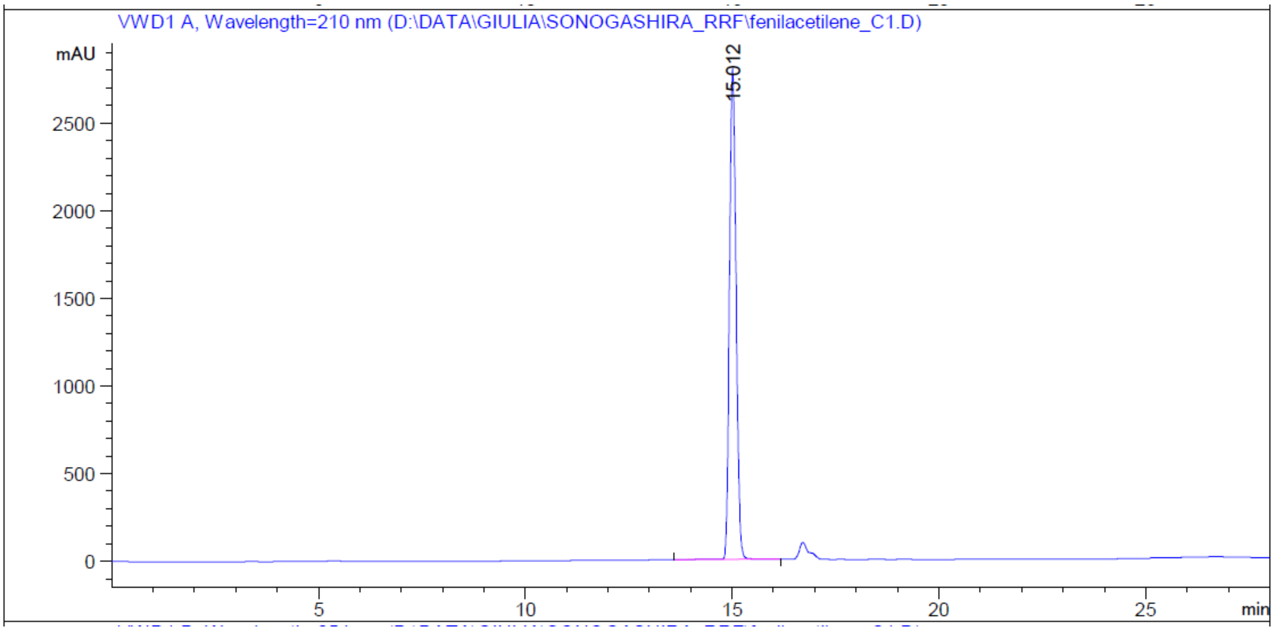

Figure S41: HPLC chromatogram of phenylacetylene **2a** at 210 nm

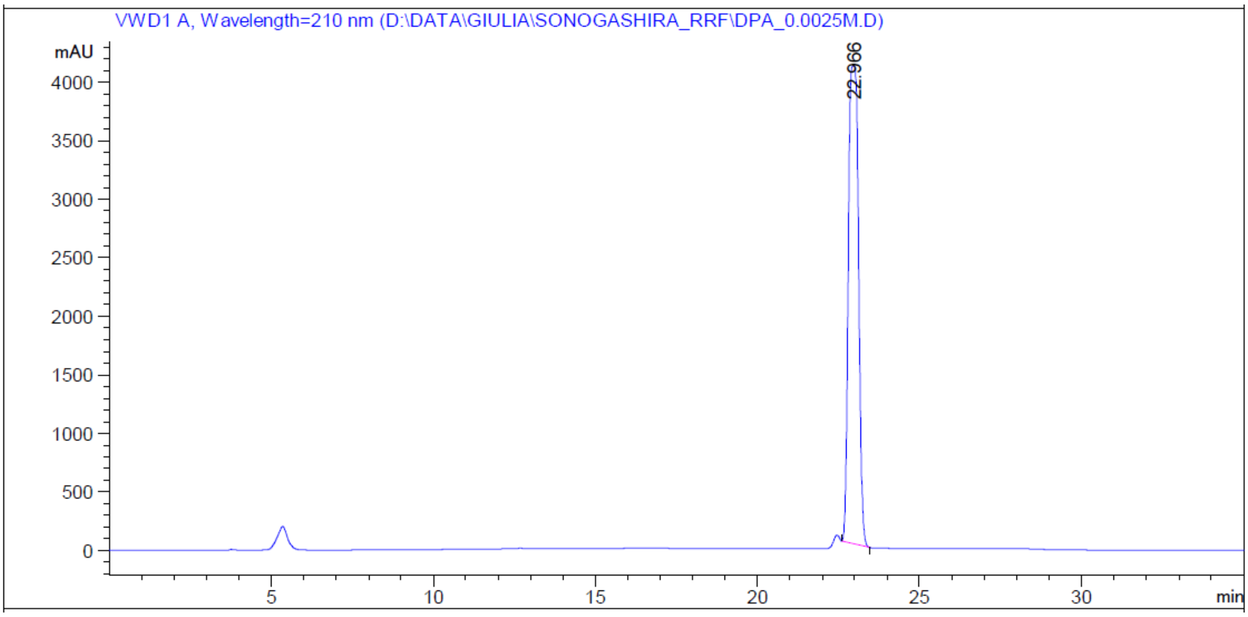

Figure S42: HPLC chromatogram of diphenylacetylene (product **3a**, reference compound) at 210 nm

- *Table 2:*

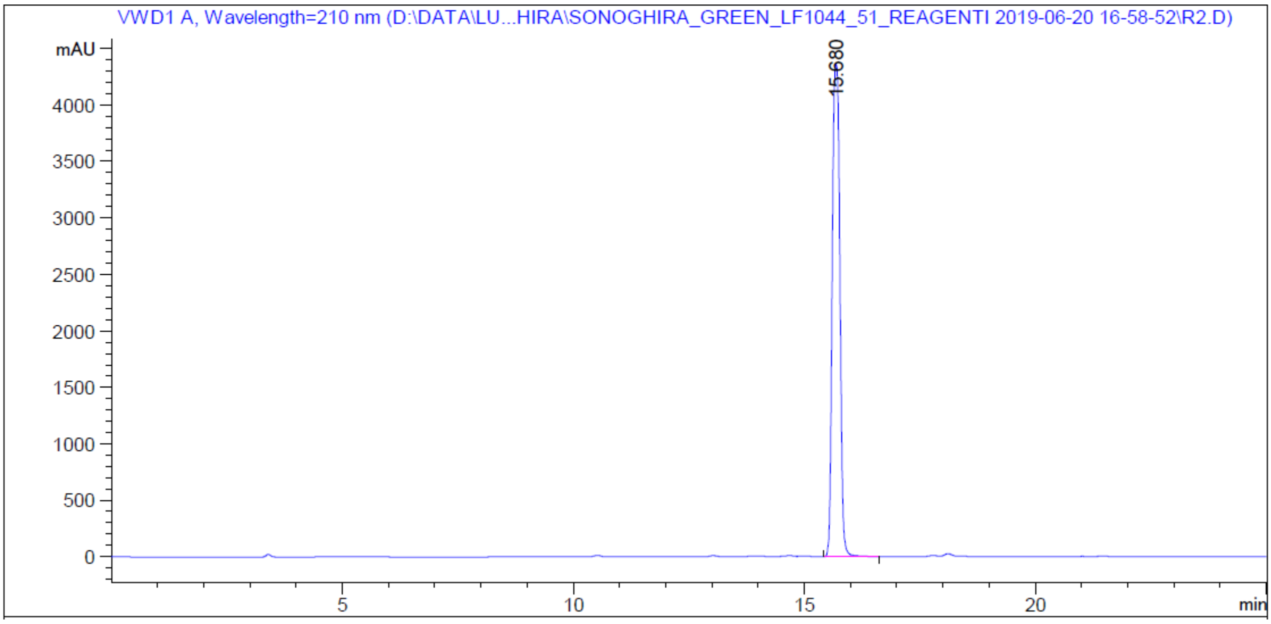

Figure S43: HPLC chromatogram of 4-nitroiodobenzene **1b** at 210 nm

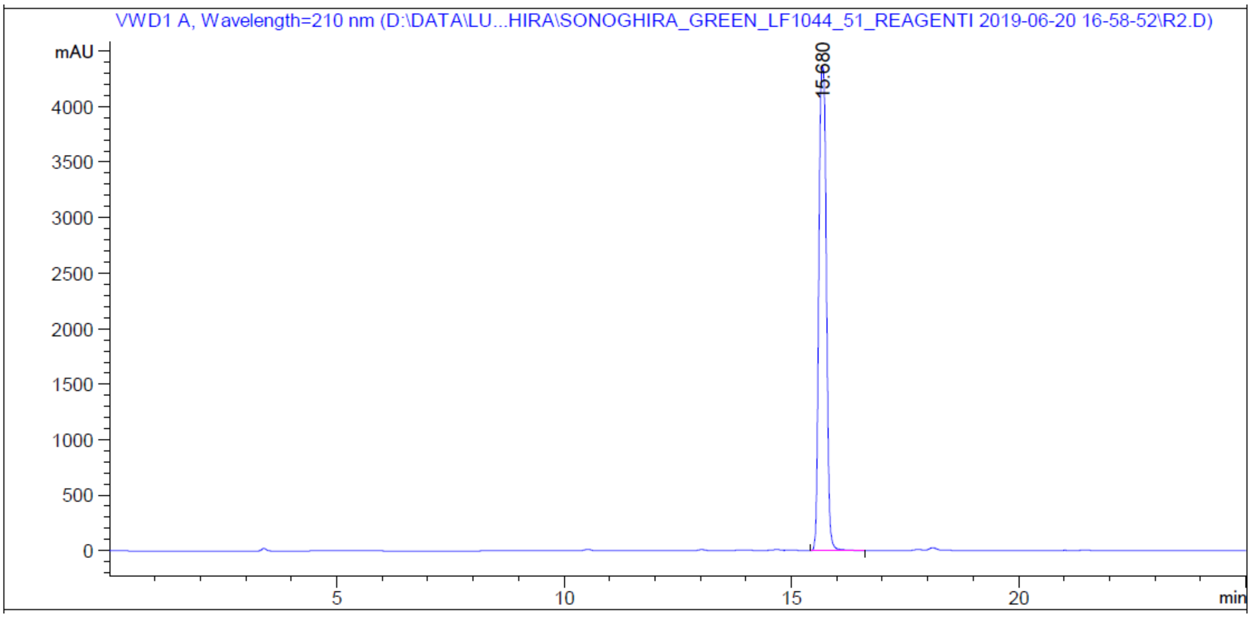

Figure S44: HPLC chromatogram of 3-nitroiodobenzene **1c** at 210 nm

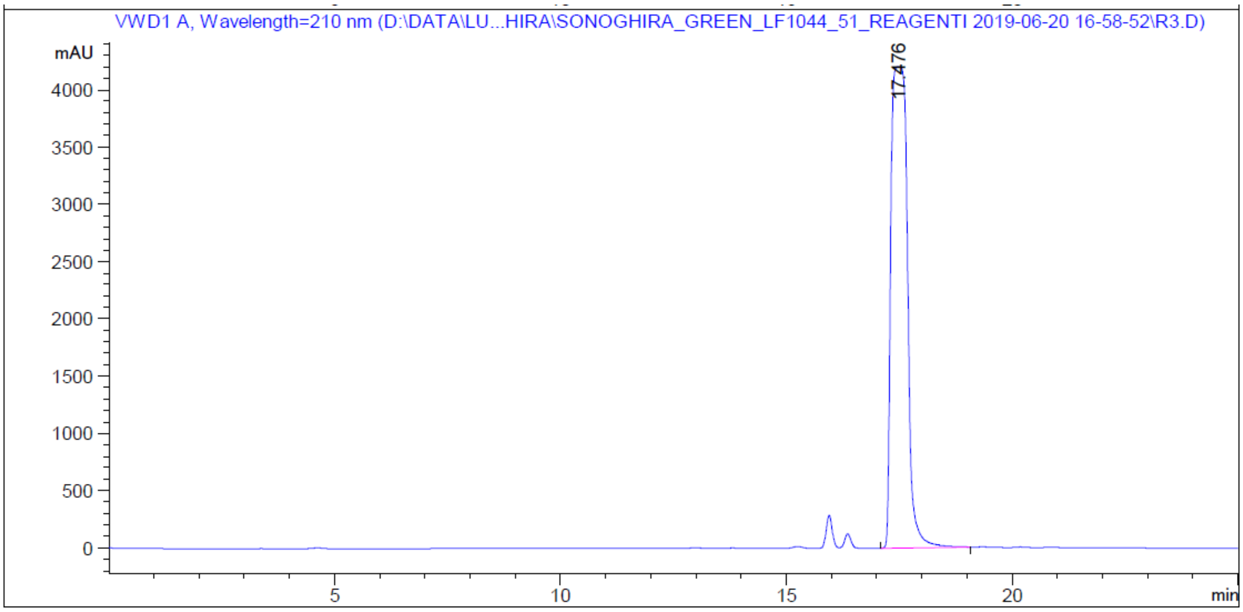

Figure S45: HPLC chromatogram of 3-methoxyiodobenzene **1d** at 210 nm

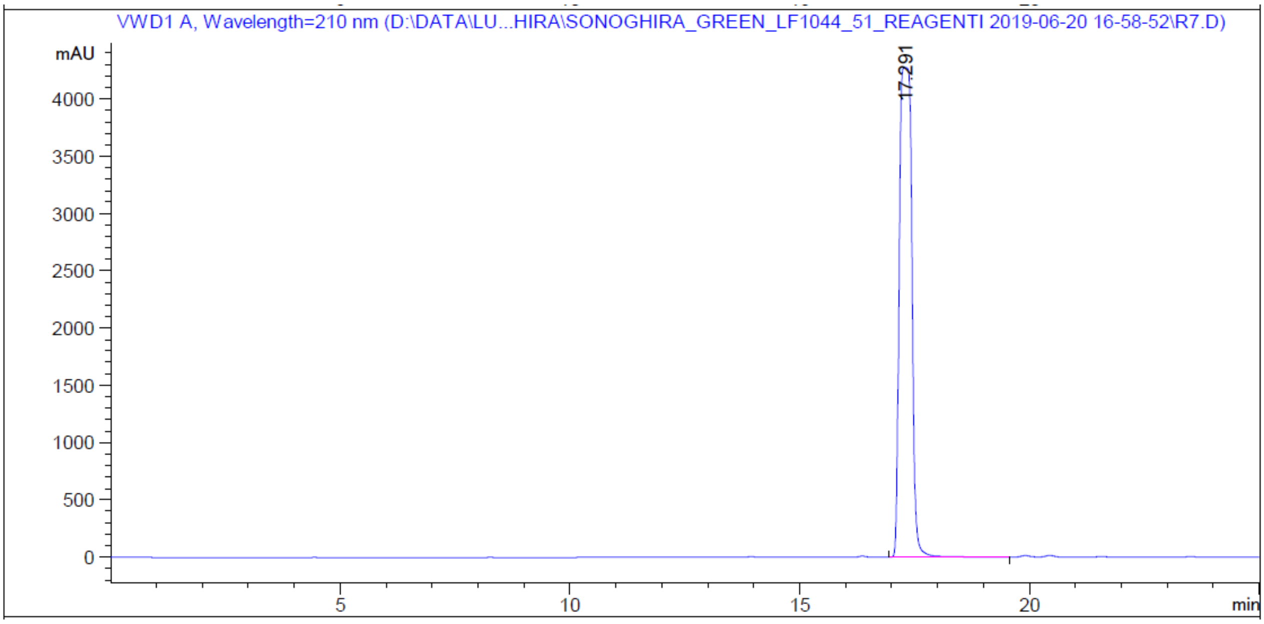

Figure S46: HPLC chromatogram of 4-methoxyiodobenzene **1e** at 210 nm

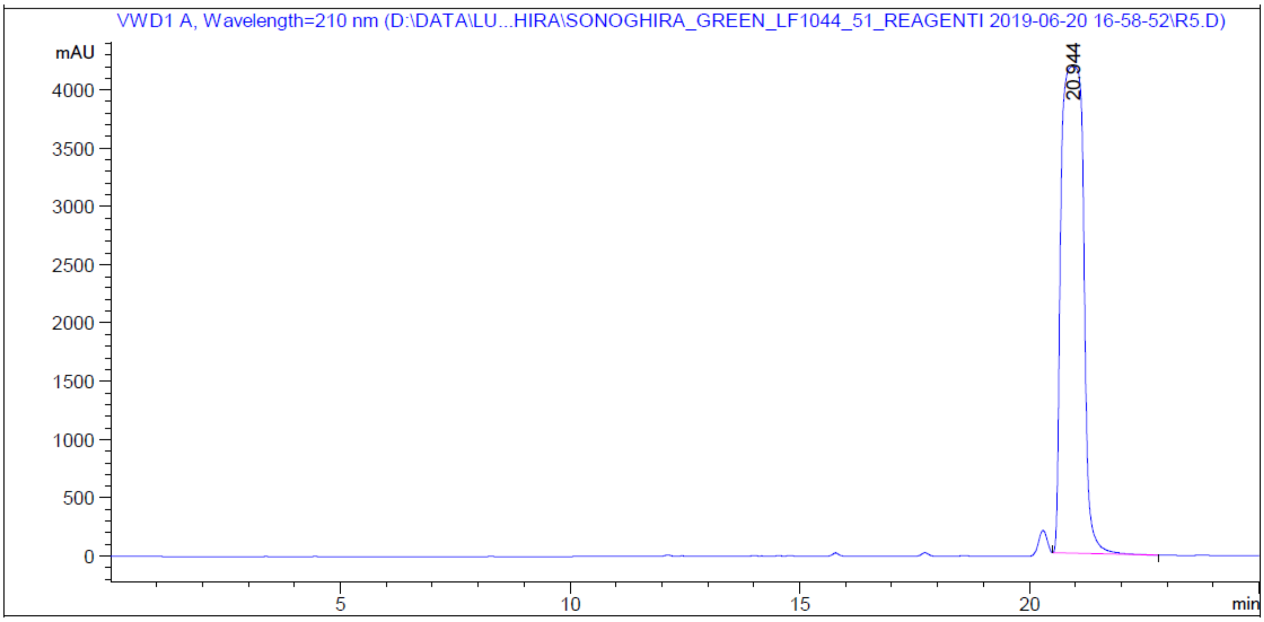

Figure S47: HPLC chromatogram of 3-chloroiodobenzene **1f** at 210 nm

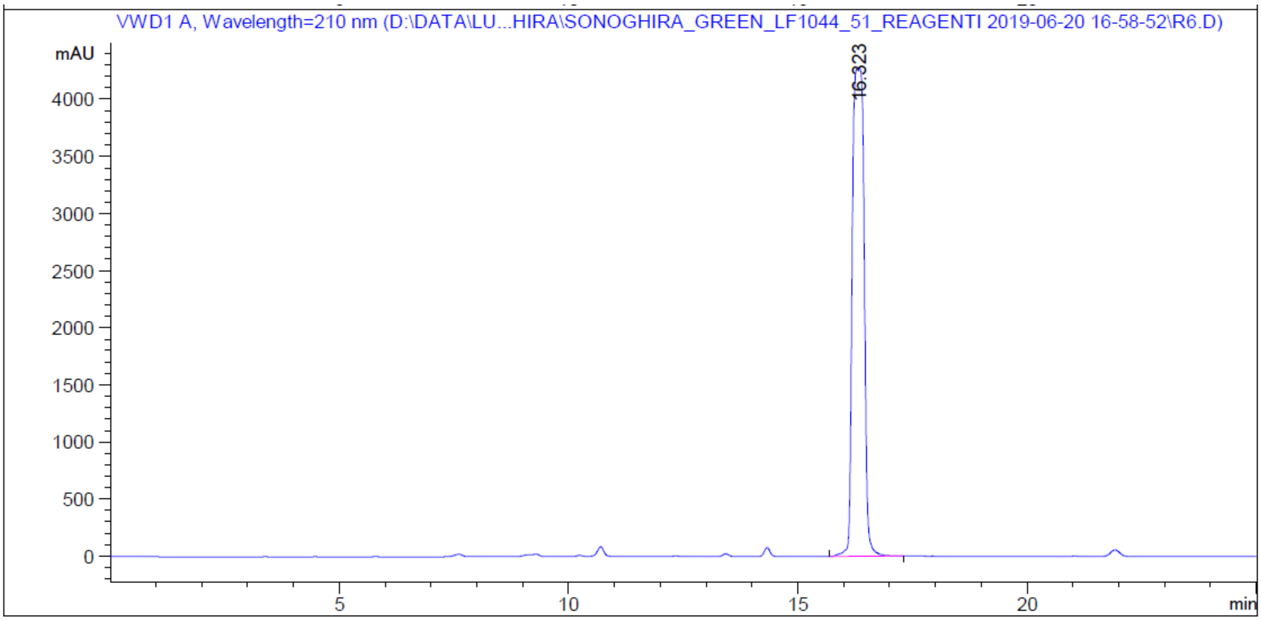

Figure S48: HPLC chromatogram of 2-iodothiophene **1g** at 210 nm

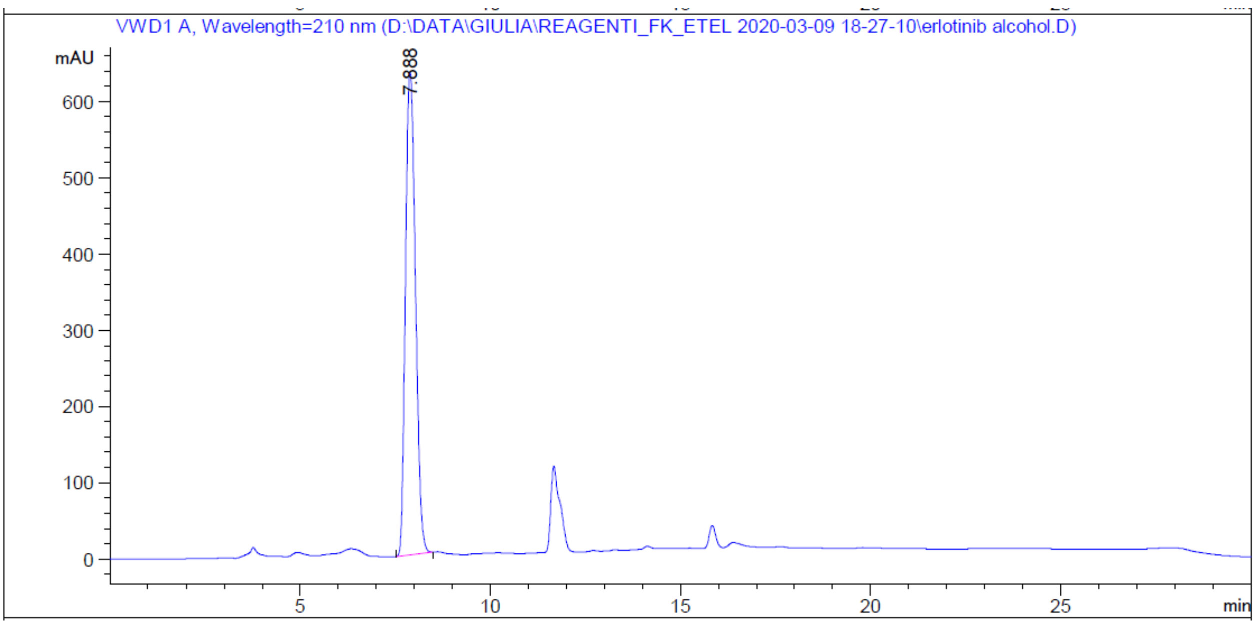

Figure S49: HPLC chromatogram of 2-methyl-3-butyn-2-ol **2h** at 210 nm

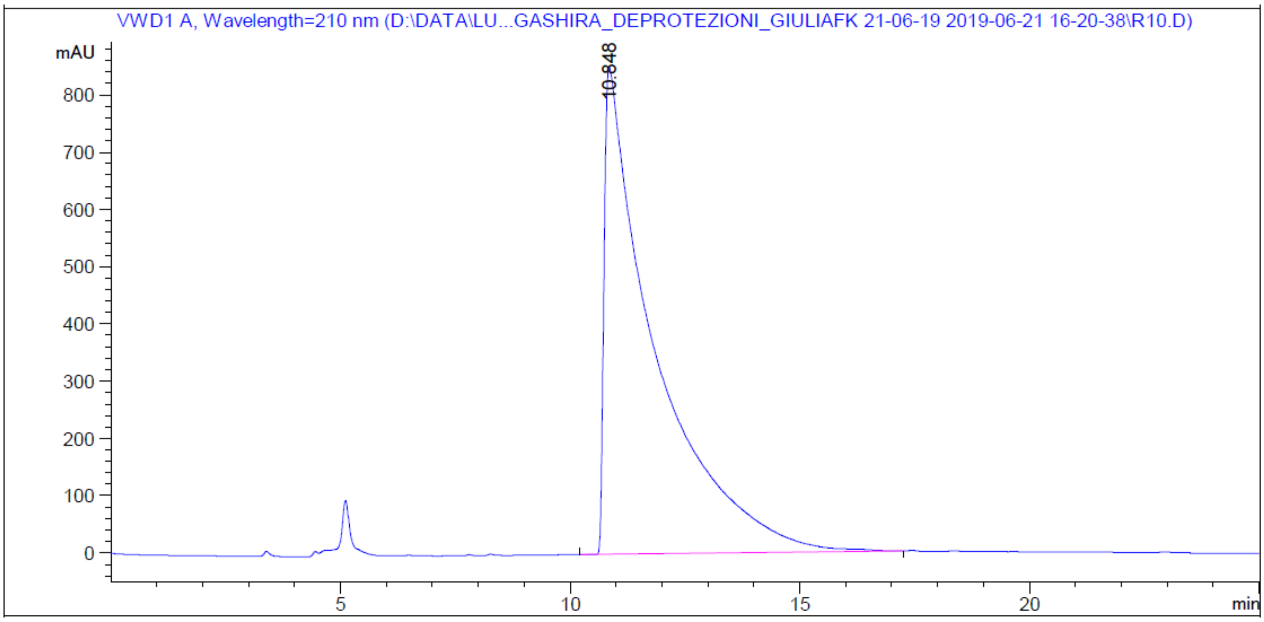

Figure S50: HPLC chromatogram of 3-dimethylamino-1-propyne **2i** at 210 nm

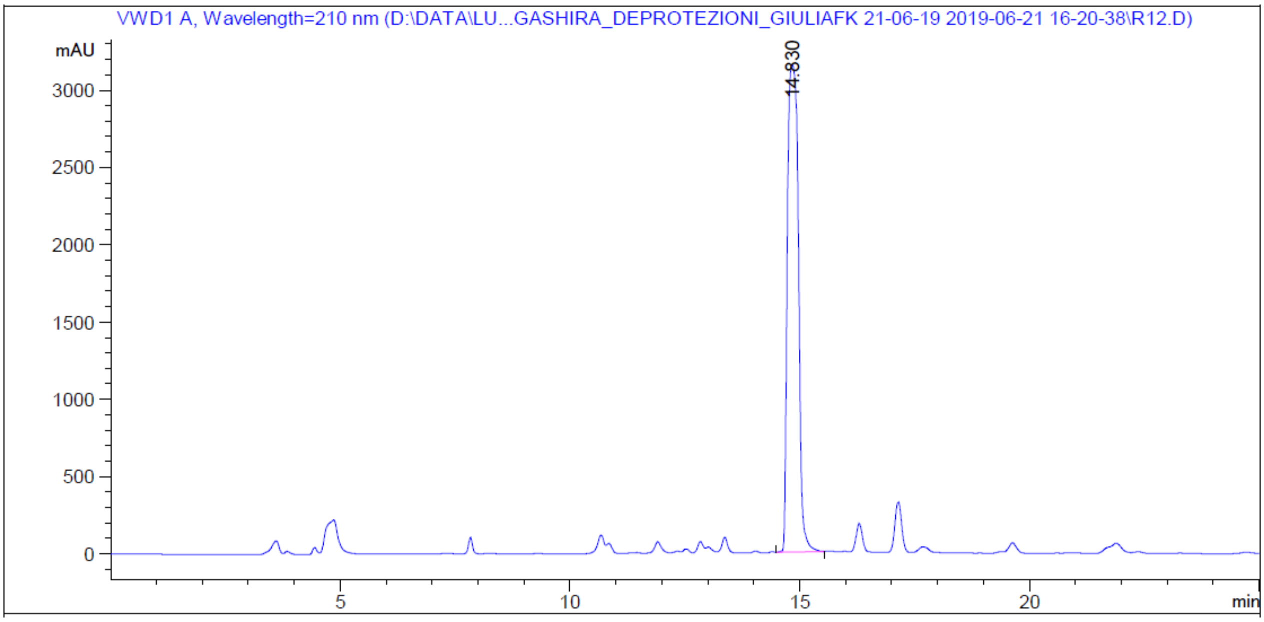

Figure S51: HPLC chromatogram of 3-phenyl-1-propyne **2j** at 210 nm

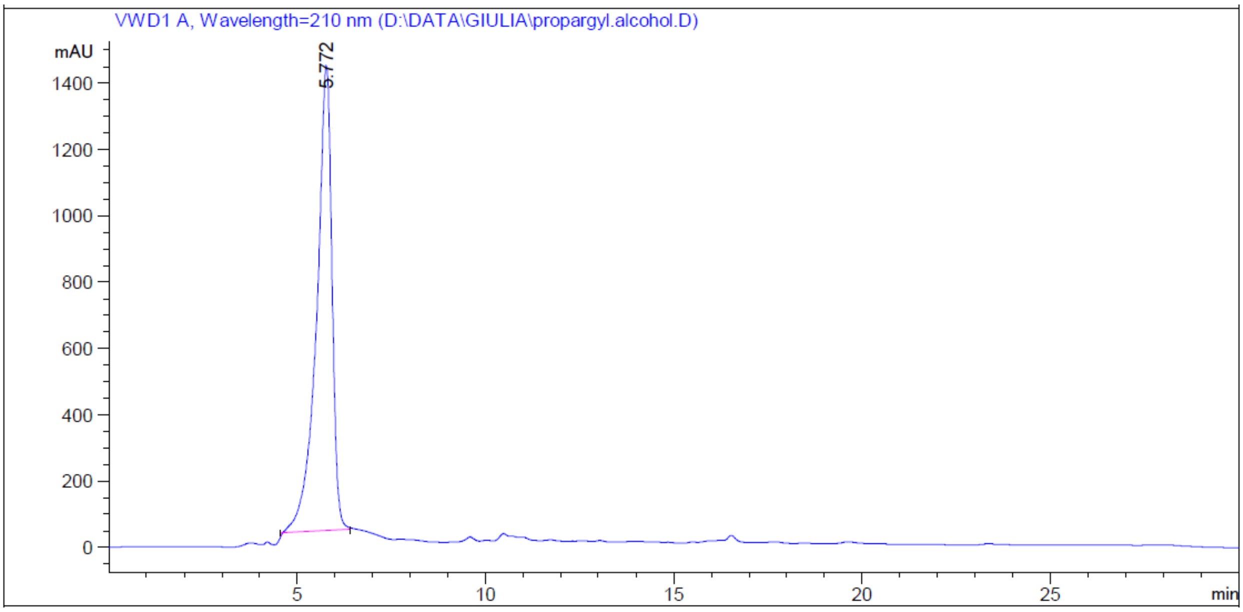

Figure S52: HPLC chromatogram of propargyl alcohol **2k** at 210 nm

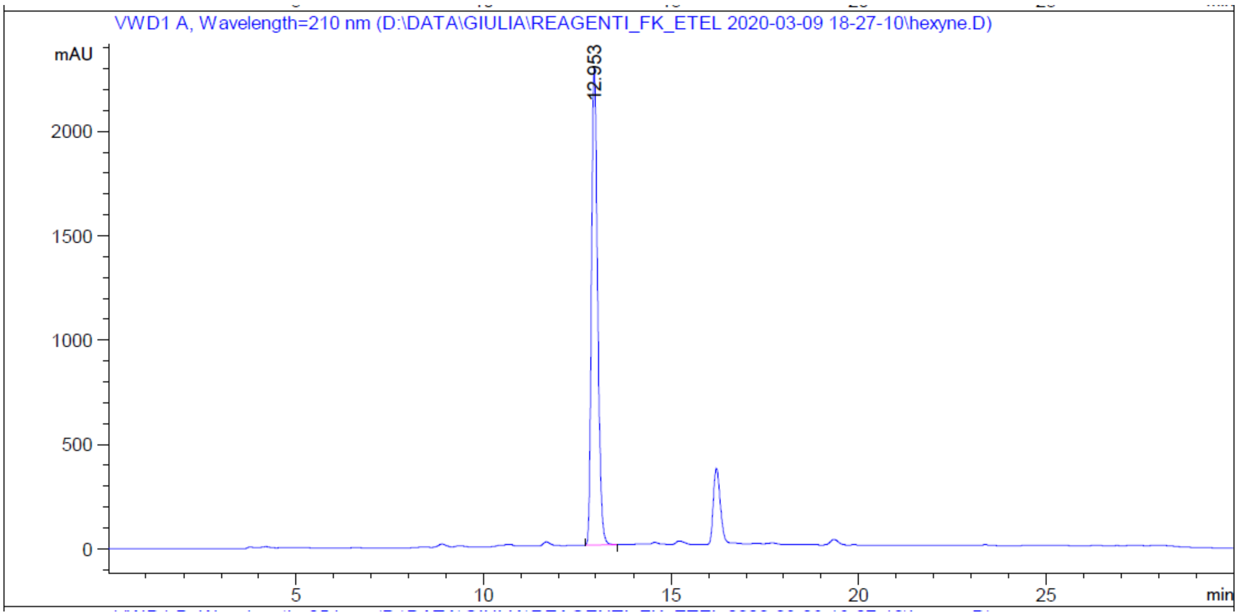

Figure S53: HPLC chromatogram of 1-hexyne **2l** at 210 nm

- *Table 3:*

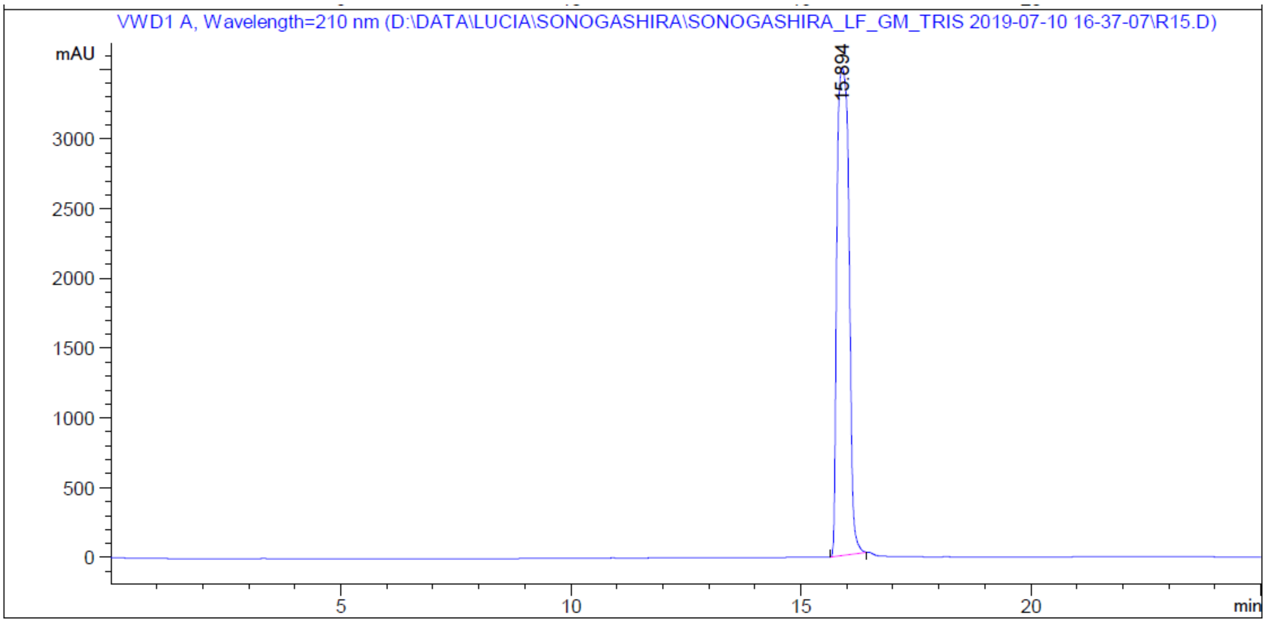

Figure S54: HPLC chromatogram of bromobenzene **4a** at 210 nm

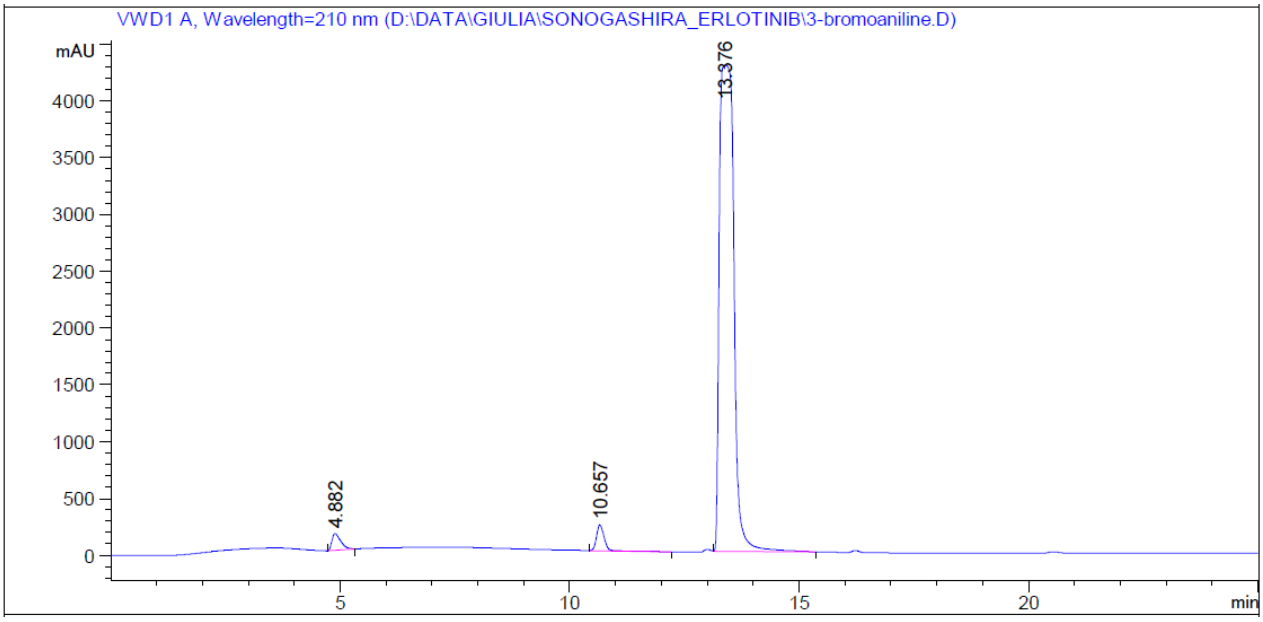

Figure S55: HPLC chromatogram of 3-bromoaniline **4b** at 210 nm

2.4 Relative response factor calculation:

Relative Response Factor between iodobenzene and diphenylacetylene or between bromobenzene and diphenylacetylene was calculated considering the peak absorption areas of an equimolar mixture of reagent and product at 210 nm (media of analysis at three different concentrations).

- *RRF between iodobenzene and diphenylacetylene*

Conc (M)	Iodobenzene Area (milliAu)	Diphenilacetylene Area (milliAu)	RRF	RRF Average value
**0,0005**	7315,2	22370,1	3,05803	**3,05496053**
**0,00025**	3682,1	11189,9	3,038999	
**0,000025**	365,5	1121,3	3,067852	

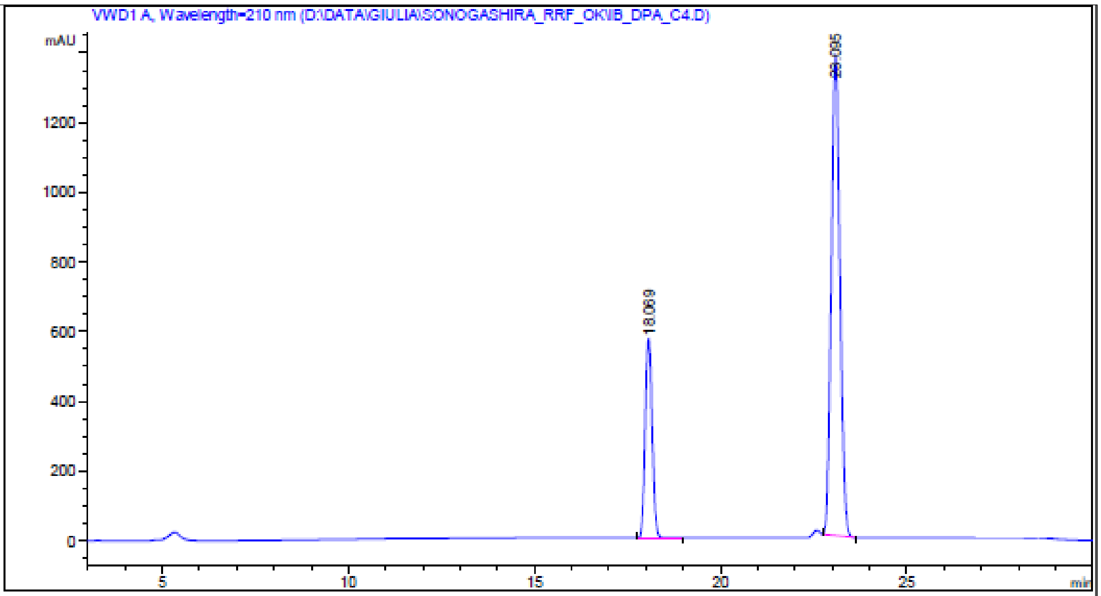


```
=====================================================================
                         Area Percent Report
=====================================================================

Sorted By             :      Signal
Multiplier            :      1.0000
Dilution              :      1.0000
Use Multiplier & Dilution Factor with ISTDs


Signal 1: VWD1 A, Wavelength=210 nm

Peak RetTime Type  Width     Area      Height     Area
  #   [min]        [min]   [mAU*s]     [mAU]        %
----|-------|----|-------|----------|----------|--------|
   1  18.069 BB    0.1994 7315.22217  571.14691  24.6425
   2  23.095 BB    0.2540 2.23701e4  1373.45581  75.3575

Totals :                  2.96854e4  1944.60272
```

Figure S56: HPLC-UV spectrum of equimolar mixture of iodobenzene and diphenylacetylene at 0.005 M concentration and integration peak values

- *RRF between bromobenzene and diphenylacetylene*

Conc (M)	Bromobenzene Area (milliAu)	Diphenylacetylene Area (milliAu)	RRF	RRF Average value
**0,0005**	8516,1	22536,4	2,646329	**2,67921768**
**0,00025**	4240,3	11310,3	2,667335	
**0,000025**	418,1	1138,9	2,723989	

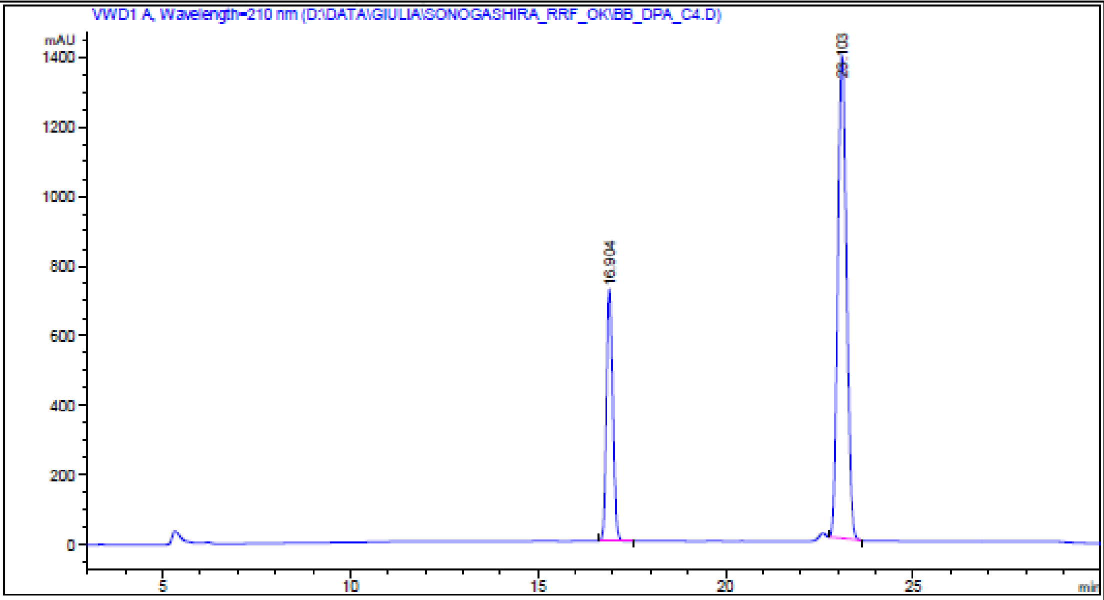


```
=====================================================================
                         Area Percent Report
=====================================================================

Sorted By             :      Signal
Multiplier            :      1.0000
Dilution              :      1.0000
Use Multiplier & Dilution Factor with ISTDs


Signal 1: VWD1 A, Wavelength=210 nm

Peak RetTime Type  Width     Area      Height     Area
  #   [min]        [min]   [mAU*s]     [mAU]       %
----|-------|----|-------|----------|----------|--------|
   1  16.904 BB    0.1842 8516.14844  718.93365  27.4250
   2  23.103 BB    0.2541 2.25364e4  1383.19104  72.5750

Totals :                  3.10526e4  2102.12469
```

Figure S57: HPLC-UV spectrum of equimolar mixture of bromobenzene and diphenylacetylene at 0.005 M concentration and integration peak values

References

[1] Raja, G. C.; Irudayanathan, F. M.; Kim, H.-S.; Kim, J.; Lee, S. *J. Org. Chem.*, **2016**, *81*, 5244-5249.

[2] Kakusawa, N.; Yamaguchi, K.; Kurita, J. *J. Organomet. Chem.*, **2005**, *690*, 2956-2966.

[3] Gallop, C. W. D.; Chen, M.-T.; Navarro, O. *Org. Lett.*, **2014**, *16*, 3724-3727.

[4] Chahdoura, F.; Pradel, C.; Gómez, M. *Adv. Synth. Catal.*, **2013**, *355*, 3648-3660.

[5] Li, X.; Liu, X.; Chen, H.; Wu, W.; Qi, C.; Jiang, H. *Angew. Chem. Int. Ed.*, **2014**, *53*, 14485-14489.

[6] Zhang, X.; Teo, W. T.; Chan, P. W. H. *Org. Lett* **2009**, *11*, 4990 – 4993.

[7] Caporale, A.; Tartaggia, S.; Castellin, A.; De Lucchi, O. *Beilstein J. Org. Chem.*, **2014**, *10*, 384 – 393.

[8] Cabrera-Lobera, N.; Quirós, M. T.; Brennessel, W. W.; Neidig, M. L.; Buñuel, E.; Cárdenas, D. J. *Org.Lett*, **2019**, *21*, 6552 – 6556.

[9] Rai, P.; Maji, K.; Maji, B. *Org. Lett.* **2019**, *21*, 3755 – 3759.

[10] Li, X.; Yang, F.; Wu, Y. *J. Org. Chem*. **2013**, *78*, 4543-4550.

[11] Zhang, J.; Zhang, J.; Hao, G.; Xin, W.; Yang, F.; Zhu, M.; Zhou, H. *J. Med. Chem*. **2019**, *62*, 6765-6784.
